# Profiling RNA at chromatin targets in situ by antibody-targeted tagmentation

**DOI:** 10.1038/s41592-022-01618-9

**Published:** 2022-10-03

**Authors:** Nadiya Khyzha, Steven Henikoff, Kami Ahmad

**Affiliations:** 1 Basic Sciences Division, Fred Hutchinson Cancer Center, Seattle, WA USA; 2 Howard Hughes Medical Institute, Chevy Chase, MD USA

**Keywords:** Chromatin analysis, Reverse transcription polymerase chain reaction, Gene expression profiling

## Abstract

Whereas techniques to map chromatin-bound proteins are well developed, mapping chromatin-associated RNAs remains a challenge. Here, we describe Reverse Transcribe and Tagment (RT&Tag), in which RNAs associated with a chromatin epitope are targeted by an antibody followed by a protein A-Tn5 transposome. Localized reverse transcription generates RNA/cDNA hybrids that are subsequently tagmented by Tn5 transposases for downstream sequencing. We demonstrate the utility of RT&Tag in *Drosophila* cells for capturing the noncoding RNA roX2 with the dosage compensation complex and maturing transcripts associated with silencing histone modifications. We also show that RT&Tag can detect N6-methyladenosine-modified mRNAs, and show that genes producing methylated transcripts are characterized by extensive promoter pausing of RNA polymerase II. The high efficiency of in situ antibody tethering and tagmentation makes RT&Tag especially suitable for rapid low-cost profiling of chromatin-associated RNAs.

## Abstract (web summary)

This work presents RT&Tag, which captures RNA molecules in proximity to a protein of interest in intact nuclei.

## Main

RNA expression levels are tightly regulated throughout their lifecycle to ensure proper biological function^1^. Factors influencing RNA posttranscriptionally include interaction with RNA-binding proteins (RBPs), location within the nucleus, and posttranscriptional modifications^1^. The most widely used strategy for assaying these factors is immunoprecipitation, whereby antibodies are used to pull down RNA associated with an epitope of interest from cell lysates^2^. The recovered RNA is then purified and used for downstream analysis such as Illumina sequencing^3,4^. Variations of the immunoprecipitation protocol have been developed to study different types of interactions between RNA and chromatin. Examples include RNA immunoprecipitation (RIP) and UV cross-linking and immunoprecipitation (CLIP) for detecting RNA–protein interactions. Chromatin-specific immunoprecipitation assays include profiling interacting RNAs on chromatin followed by deep sequencing (PIRCh-seq) and Chromatin RIP followed by high-throughput sequencing (ChRIP-seq), which crosslink RNA to chromatin and assay RNA–chromatin interactions using antibodies targeting histone posttranslational modifications^5,6^. Immunoprecipitation assays for N^6^-methyladenosine (m6A)-modified RNA include methylated RNA immunoprecipitation with next-generation sequencing (MeRIP-seq) and m6A-RIP-seq^7,8^. Unfortunately, these immunoprecipitation-based methods require large sample inputs and optimization of cross-linking conditions^2,9^. There is a need for sensitive in situ technologies that do not rely on cross-linking or immunoprecipitation to capture endogenous RNA interactions.

Cleavage under targets and tagmentation (CUT&Tag) is an enzyme-tethering strategy developed to profile the binding sites of chromatin proteins within intact nuclei^10^. CUT&Tag bypasses immunoprecipitation and instead uses antibodies to tether a protein A-Tn5 transposase fusion protein in situ. Tn5 undergoes a tagmentation reaction where genomic DNA is cleaved and tagged with sequencing adapters. These sequencing adapters are then used to generate Illumina sequencing libraries. In addition, Tn5 also contains an RNase H-like domain that can bind and tagment reverse transcribed RNA/cDNA hybrids^11,12^. This finding inspired us to develop reverse transcribe and tagment (RT&Tag)—a proximity labeling tool for capturing RNA interactions within intact nuclei. RT&Tag follows the framework of CUT&Tag but is adapted to capture signal from RNA instead of genomic DNA. Relative to RIP-based immunoprecipitation methods, RT&Tag requires fewer cells and a smaller number of sequencing reads, while capturing interactions within intact nuclei. In this work, we demonstrate the general utility of RT&Tag by applying it to a variety of RNA- and chromatin-dependent biological processes in *Drosophila* S2 nuclei. Specifically, we use RT&Tag to target the dosage compensation complex, the polycomb chromatin domains, and m6A RNA posttranscriptional modification. Surprisingly, we find that binding of the m6A writer, METTL3, is not sufficient for RNA methylation. Instead, we find that RNA polymerase II (RNAPolII) pausing is a strong predictor of m6A mark deposition. This finding illustrates the potential of RT&Tag to empower research in the fields of epigenetics and RNA biology.

## Results

### RT&Tag general workflow

**Fig. 1:**
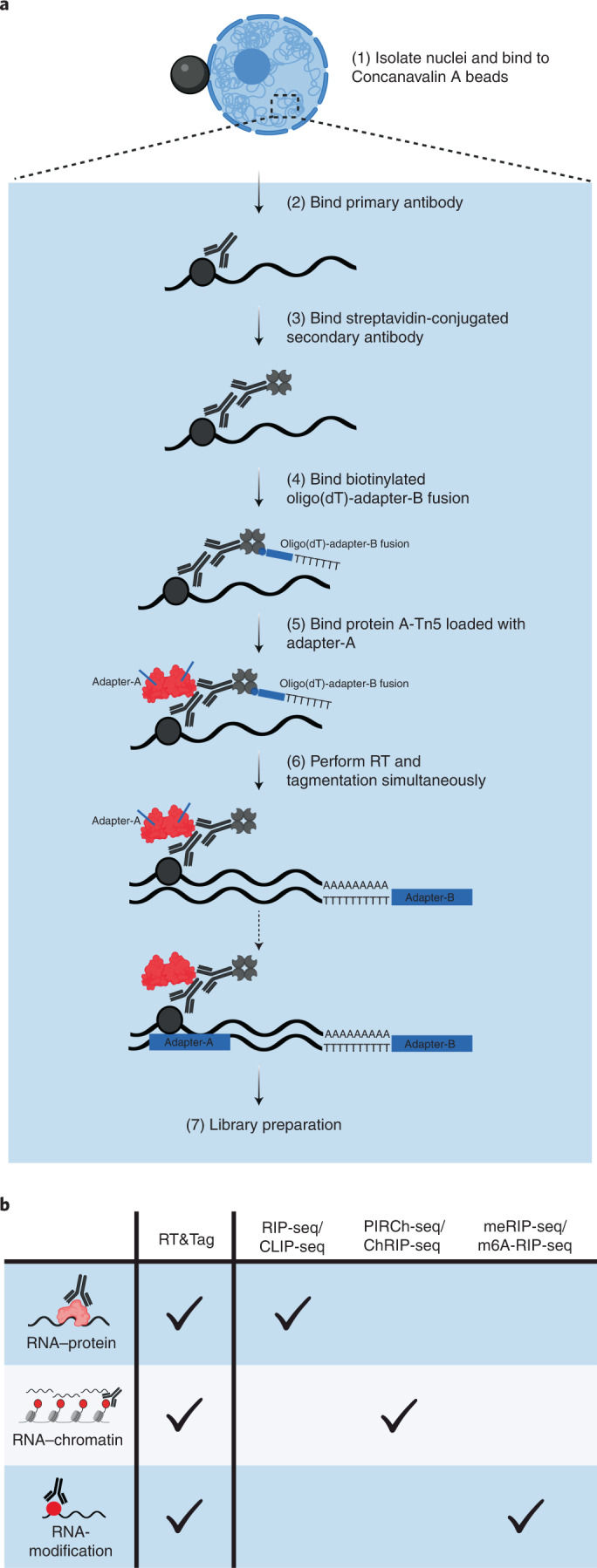
RT&Tag general workflow. **a**, Schematic outlining the steps of RT&Tag. **b**, Illustration showing applications of RT&Tag described in this work in contrast to immunoprecipitation-based techniques which require a separate method for targeting each type of interaction.

**Extended Data Fig. 1:**
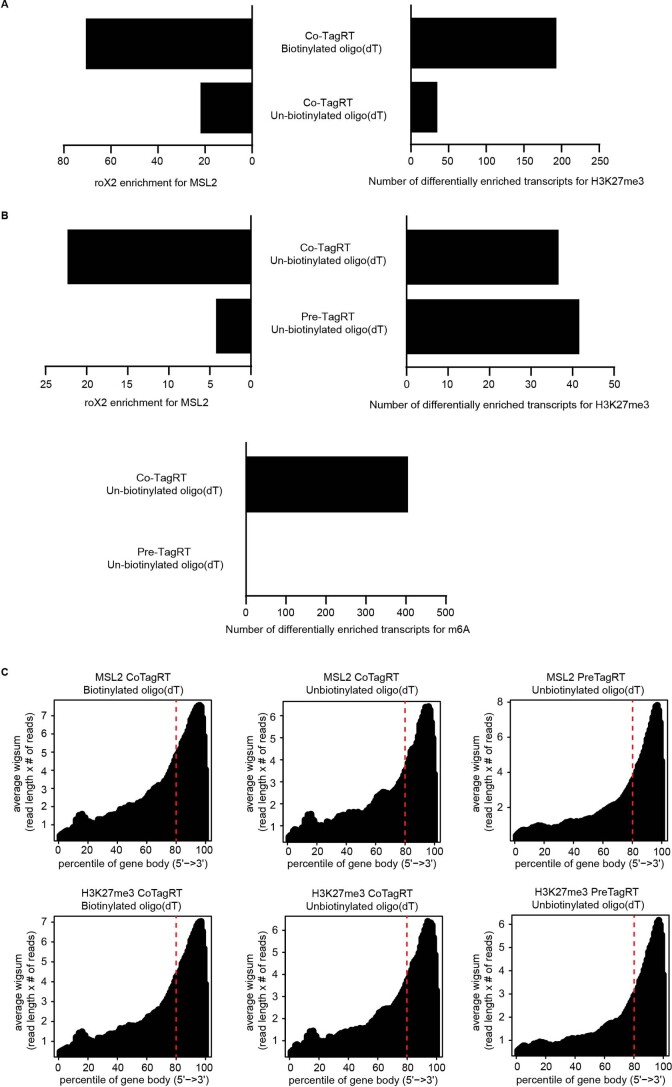
Optimization of RT&Tag. **a**) Performance comparison of RT&Tag using biotinylated or un-biotinylated oligo(dT)-adapter B fusion oligonucleotides based on the following metrics: roX*2* enrichment for MSL2 (left) and number of differentially enriched transcripts for K27me3 (right) based on 2 replicates. Both experiments were performed using reverse transcription performed at the same time as tagmentation (CoTagRT) approach. **b**) Performance comparison of RT&Tag if reverse transcription is performed prior to addition of pA-Tn5 (preTagRT) or if reverse transcription is performed at the same time as tagmentation (CoTagRT). Both experiments were performed using un-biotinylated oligo(dT)-adapter B fusion oligonucleotides. Performance of RT&Tag was assessed based on the following metrics: roX2 enrichment for MSL2 (top left), number of differentially enriched transcripts for K27me3 (top right) and number of differentially enriched transcripts for m6A (bottom) with pre-TagRT versus Co-TagRT based on 2 replicates. Differential enrichment was defined as >2-fold change for K27me3 or >1.5-fold change for m6A, <0.05 FDR. **c**) Density plots showing the distribution of aligned MSL2 (top) and H3K27me3 (bottom) RT&Tag reads (n = 2) scaled over Drosophila gene bodies for biotinylated oligo(dT) CoTagRT (left), unbiotinylated oligo(dT) CoTagRT (center), and unbiotinylated oligo(dT) preTagRT (right) RT&Tag variations. A clear bias towards the 3’ end of genes is observed under all conditions. Source data

**Extended Data Fig. 2:**
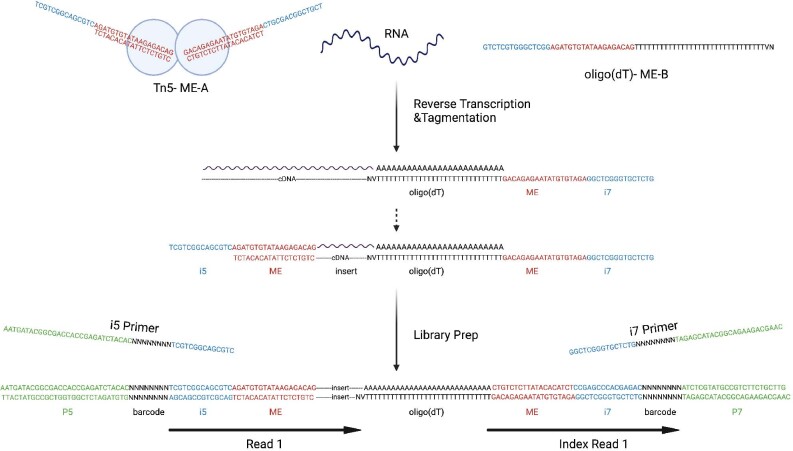
Construction of RT&Tag libraries. Schematic showing how RT&Tag libraries are generated. During reverse transcription the oligo(dT)-ME-B fusion oligonucleotide binds to the poly(A) tail of RNA. Anchored oligo(dT) is used to ensure binding at the start of the poly(A) tail. Through the process of reverse transcription, the ME-B sequence gets appended to the cDNA. The RNA/cDNA hybrid then gets tagmented with ME-A loaded Tn5. Sequencing libraries are then amplified using primers complementary to the i5 and i7 sequences. The libraries are sequenced using 50 base pair single-end sequencing with the read originating from the i5 side.

To create a method analogous to CUT&Tag for detecting localized RNAs, we capitalized on the ability of Tn5 to tagment RNA/DNA hybrid duplexes^11,12^. We first isolated nuclei and bound a factor-specific primary antibody. Next, we added a streptavidin-conjugated secondary antibody, which binds to the primary antibody. We then added biotinylated oligo(dT)-adapter primers and pA-Tn5 loaded with a second adapter, both of which bind to the secondary antibody (Fig. 1a). Using biotinylated oligo(dT)-adapter fusions increases the signal-to-noise ratio by selectively priming nearby RNA for reverse transcription (RT) (Extended Data Fig. 1a). Addition of reverse transcriptase then converts mature transcripts near the binding site to RNA/DNA hybrids, which are tagmented by the juxtaposed pA-Tn5. RT and tagmentation are then performed within one incubation step in a compatible buffer. With simultaneous RT and tagmentation, we were able to detect higher transcript enrichment than with sequential RT and tagmentation (Extended Data Fig. 1b). This may be attributed to RT altering RNA secondary structure, which could then disrupt RNA–protein interactions or mask epitope binding sites. Hence, the simultaneous RT and tagmentation approach may preserve endogenous RNA interactions until the time of tagmentation without sacrificing RT efficiency (Extended Data Fig. 1c). After RT and tagmentation, the pA-Tn5 is stripped off with SDS and the sequencing libraries are amplified using PCR. To generate sequencing libraries only from RNA instead of from genomic DNA, the i7 adapter sequence is appended to the 5′ end of the oligo(dT) sequence, ensuring its integration into all reverse transcribed transcripts (Extended Data Fig. 2). The i5 adapter is loaded into Tn5 and is integrated into RNA/cDNA hybrids via tagmentation. As such, only tagmented RNA/cDNA hybrids have both adapters necessary for library amplification, whereas genomic DNA lacks the i7 adapter. With the i7 adapter appended to the oligo(dT), the amplified libraries should detect signal from the 3′ end of the RNA. This means that only a small segment of the RNA needs to be effectively reverse transcribed to be detected by RT&Tag. Not having to reverse transcribe the entirety of the transcripts minimizes variation arising from RT such as interference with the processivity of the reverse transcriptase due to RNA secondary structure, protein binding and RNA length. To explore the capabilities of RT&Tag, we have applied it to address diverse problems in RNA–chromatin biology (Fig. 1b).

### RT&Tag captures the interaction between MSL2 and roX2

**Fig. 2:**
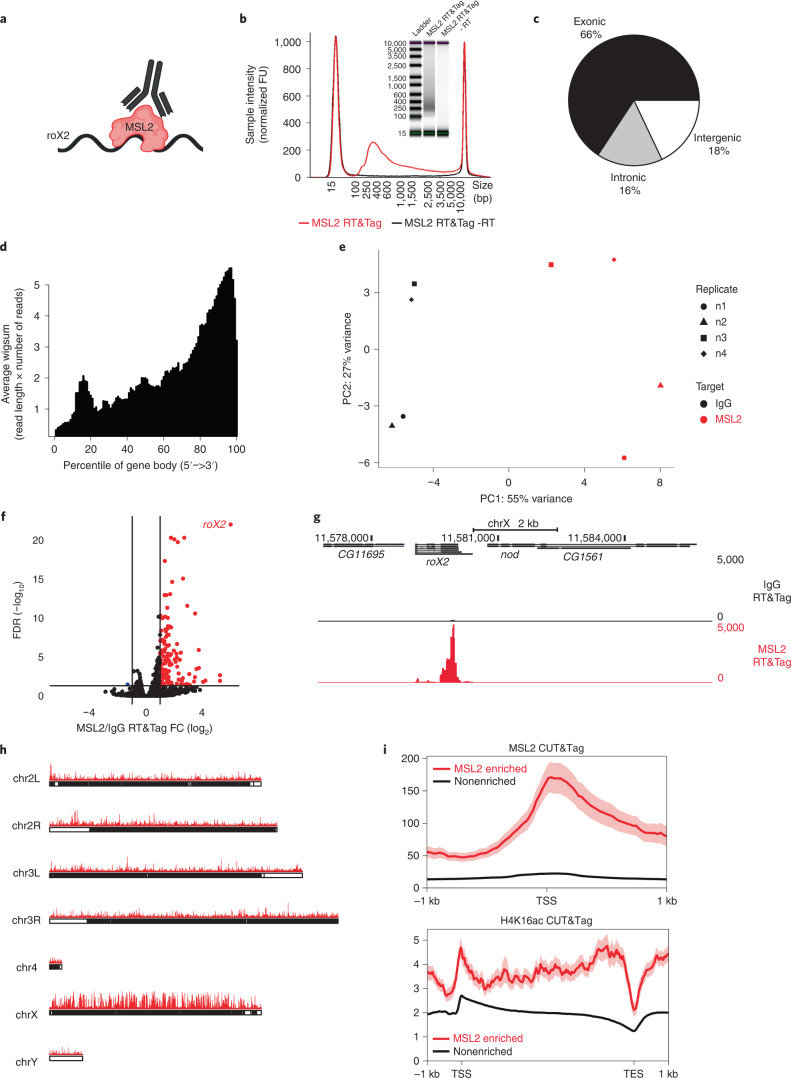
RT&Tag captures the interaction between MSL2 and roX2. **a**, Illustration showing RT&Tag being used to capture the interaction between MSL2 and roX2. **b**, Tapestation gel image and corresponding electropherogram showing size distribution of the MSL2 RT&Tag libraries after two rounds of 0.8× bead cleanup. This image is representative of two independent experiments. FU (fluorescence units). **c**, Pie chart showing the proportion of MSL2 RT&Tag reads (*n* = 4) aligning to regions classified as exonic, intronic or intergenic. **d**, Density plot showing the distribution of aligned MSL2 RT&Tag reads (*n* = 4) scaled over *Drosophila* gene bodies. **e**, PCA showing separation between IgG and MSL2 RT&Tag samples (*n* = 4) along the first principal component (PC1) and separation between replicates in the second principal component (PC2). The first two and last two replicates have been sequenced on two separate flow cells and hence a batch effect may be observed. **f**, Volcano plot showing transcripts differentially enriched for MSL2 over IgG RT&Tag (FC >2, FDR < 0.05, *n* = 4). Transcripts enriched for MSL2 are highlighted in red, nonenriched are in black and depleted are in blue. **g**, Genome browser track showing the distribution of MSL2 and IgG RT&Tag signal over the gene body of *roX2*. Combined reads from four replicates are shown. **h**, Karyoplots showing the bins (50 bp) where MSL2 RT&Tag signal is fourfold over IgG plotted (*n* = 4) over the *Drosophila* chromosomes. **i**, Profile plots showing the MSL2 (top) and H4K16ac (bottom) CUT&Tag signal around the TSS (top) and gene bodies (bottom) of MSL2 RT&Tag-enriched or nonenriched transcripts. Combined reads from two replicates for MSL2 CUT&Tag and one replicate for H4K16ac CUT&Tag are shown. Error bands indicate standard error. Source data

**Extended Data Fig. 3:**
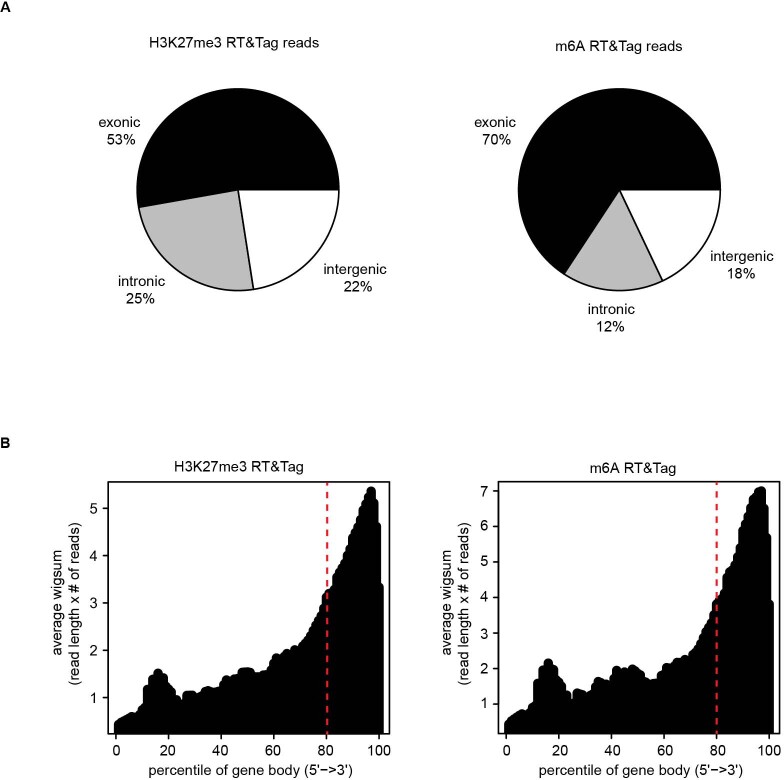
H3K27me3 and m6A RT&Tag signal. **a**) Pie chart showing the proportion of H3K27me3 (left, n = 5) and m6A (right, n = 3) RT&Tag reads aligning to regions classified as either exonic, intronic, or intergenic. **b**) Density plots showing the distribution of aligned H3K27me3 (left, n = 5) and m6A (right, n = 3) RT&Tag reads scaled over Drosophila gene bodies. Source data

As a proof of concept, we used antibodies to target the RNA-associated dosage compensation complex in the male *Drosophila* S2 cell line (Fig. 2a). The MSL complex coats the male X chromosome to upregulate gene expression by depositing the activation-associated H4K16ac mark^13^. The long noncoding RNA (lncRNA) roX2 is bound by MSL2—an interaction that we could detect using RT&Tag^13^. Using an anti-MSL2 antibody, we generated RT&Tag DNA sequencing libraries. Four features indicated that these libraries resulted from tagmentation of reverse transcribed RNA/DNA hybrids. As shown in Fig. 2b, no libraries were produced when reverse transcriptase was omitted. While CUT&Tag for chromatin targets produced a nucleosomal ladder, RT&Tag libraries had a broad size distribution ranging predominantly from 200 base pairs (bp) to 1,000 bp with no nucleosomal pattern. Furthermore, mapped RT&Tag reads were primarily of exonic origin (66%) with a small number of intronic (16%) and intergenic reads (18%) (Fig. 2c and Extended Data Fig. 3a). Finally, reads mostly fell at the 3′ ends of gene bodies consistent with priming from the poly-A tail of mature transcripts by the oligo-dT-adapter fusion (Fig. 2d and Extended Data Fig. 3b). Altogether, these findings demonstrate that the RT&Tag signal is exclusively from RNA.

**Extended Data Fig. 4:**
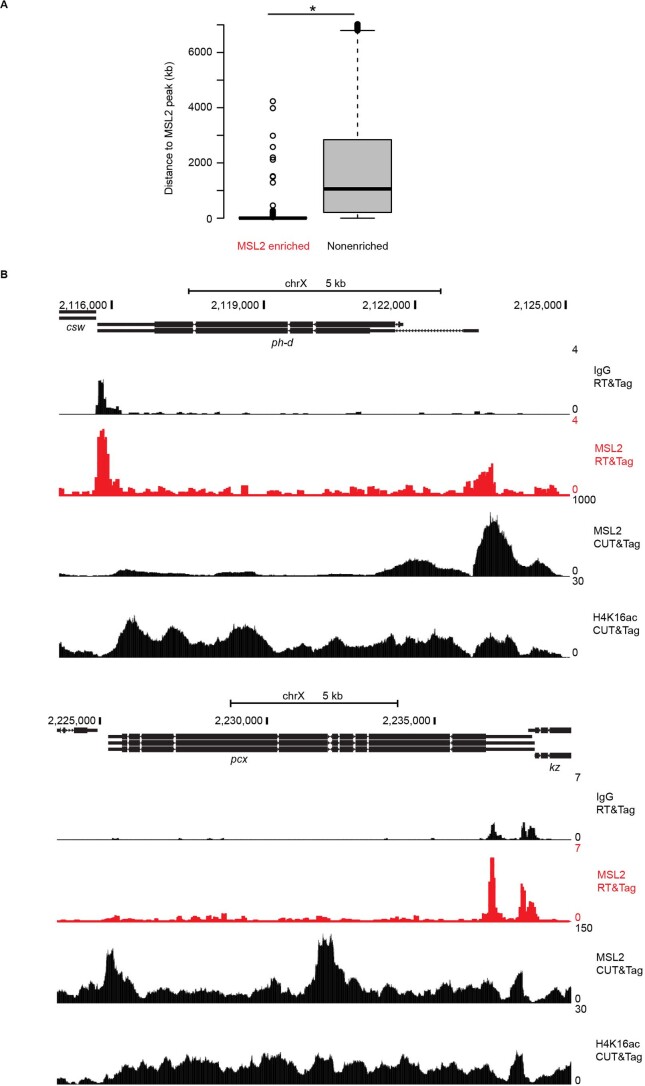
RT&Tag captures the interaction between MSL2 and transcripts within its vicinity. **a**) Boxplot showing the genomic distance from the gene body of MSL2 enriched or nonenriched transcripts to the nearest MSL2 peak. *p < 2.2 × 10^−16^, Welch Two Sample t-test (two-sided), n = 121 for MSL2 enriched transcripts, n = 13510 for nonenriched transcripts, n = 4 for MSL2 RT&Tag, n = 2 for MSL2 CUT&Tag. For the boxplots, the IQR is shown within the limits of the box, the center line represents the median, the whiskers show data that is within 1.5 times the IQR, and outliers are omitted. **b**) Genome browser tracks showing the distribution of IgG and MSL2 RT&Tag signal as well as MSL2 and H4K16ac CUT&Tag signal over the *ph-d* and *pcx* gene bodies. Combined reads from 2 replicates for MSL2 and 1 replicate for H4K16ac CUT&Tag are shown. Source data

The performance of MSL2 RT&Tag was then evaluated. Differences between MSL2 RT&Tag and the IgG background control were assessed using principal component analysis (PCA) (Fig. 2e). The first principal component captured a clear separation (55% variance) between IgG and MSL2 libraries. This separation was greater than that for the second principal component, which captured the variability between replicates (27% variance). Differential enrichment of MSL2-targeted transcripts over IgG (greater than twofold change (FC), < 0.05 false discovery rate (FDR)) identified 121 transcripts, of which roX2 showed very high enrichment and statistical significance (67 FC, <1 × 10^−22^ FDR; Fig. 2f and Supplementary Table 1). This enrichment of MSL2 RT&Tag signal over IgG is illustrated over the gene body of *roX2* using UCSC genome browser tracks, highlighting a clear 3′ bias in the distribution of reads (Fig. 2g). Apart from roX2, 120 transcripts were differentially enriched for MSL2. The MSL2 RT&Tag signal normalized for IgG showed a strong preference for the X chromosome (56.3% of greater than fourfold enriched bins; Fig. 2h). Given that MSL2 binds across the X chromosome, we asked whether MSL2 RT&Tag captured RNA that was transcribed proximal to these MSL2 binding sites. Hence, we mapped the MSL2 CUT&Tag signal at the transcriptional start sites (TSSs) of MSL2-enriched or nonenriched transcripts. Additionally, H4K16ac CUT&Tag signal was mapped over the gene bodies of MSL2-enriched or nonenriched transcripts. Higher MSL2 and H4K16ac CUT&Tag signal was observed for MSL2 RT&Tag-enriched than nonenriched transcripts, supporting our hypothesis (Fig. 2i). Furthermore, 75% of MSL2-enriched transcripts were within 13 kb of an MSL2 binding peak, which is much closer than for nonenriched transcripts (12,608 bp versus 2,841,851 bp, *P* < 2.2 × 10^−16^; Extended Data Fig. 4a). As an example, MSL2 and H4K16ac CUT&Tag signal can be seen over the gene bodies of MSL2 RT&Tag-enriched transcripts, *ph-d* and *pcx* (Extended Data Fig. 4b). Overall, these results show that RT&Tag recapitulates the well-known MSL2-roX2 interaction and captures interactions between MSL2 and transcripts found within its vicinity. roX2 is a unique outlier both in having the highest FC and the highest FDR (Fig. 2f), suggestive of a direct interaction with MSL2, while the weakly enriched or low FDR transcripts found throughout the X chromosome are likely proximity interactions.

**Extended Data Fig. 5:**
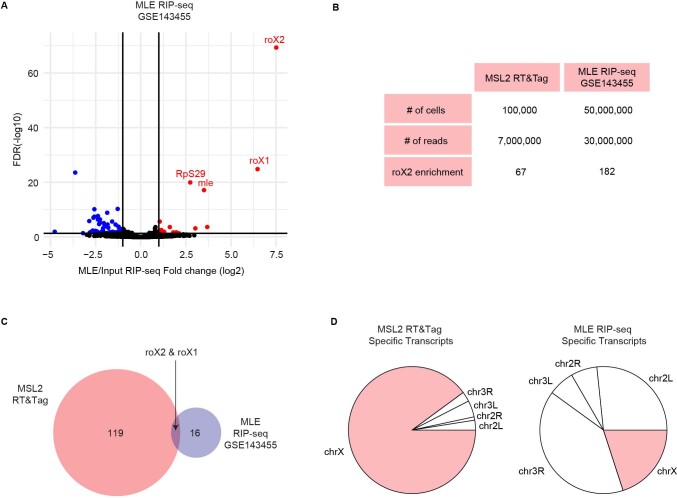
Performance comparison of MSL2 RT&Tag to RIP–seq. **a**) Volcano plot showing transcripts differentially enriched for MLE RIP–seq over input (fold change >2, FDR < 0.05, n = 3, GSE143455). Transcripts enriched for MLE are highlighted in red, nonenriched are in black, and depleted are in blue. **b**) Table comparing MSL2 RT&Tag and MLE RIP–seq in terms of number of cells, number of reads, and roX2 fold change enrichment for MSL2/MLE over control. **c**) Venn diagram showing the overlap between transcripts enriched for MSL2 RT&Tag and MLE RIP–seq with roX1 and roX2 being enriched in both. D) Pie charts showing the chromosomal distribution of transcripts uniquely enriched for MSL2 RT&Tag (left) and MLE RIP–seq (right). Source data

We then compared our MSL2 RT&Tag data with a published RIP-seq dataset, which targeted a subunit of the *Drosophila* MSL complex maleless (MLE). Like RT&Tag, MLE RIP-seq was able to identify the interaction between MLE and roX2 in S2 cells (Extended Data Fig. 5a). However, to achieve a comparable degree of enrichment for roX2, RIP-seq required 500 times the number of cells and 4 times as many sequencing reads as RT&Tag (Extended Data Fig. 5b). Apart from the roX RNAs, RT&Tag and RIP-seq picked up transcripts that were unique to each method (Extended Data Fig. 5c). Transcripts unique to RT&Tag were transcribed predominantly from the X chromosome unlike the transcripts unique to RIP-seq (Extended Data Fig. 5d). This comparison highlights the fundamental difference between RT&Tag and immunoprecipitation-based methods. Being a proximity labeling technique, RT&Tag can pick up transcripts near MSL complex binding sites, whereas RIP-seq captures binding interactions within cell lysates, some of which might not occur under endogenous conditions.

### RT&Tag captures transcripts within polycomb domains

**Fig. 3:**
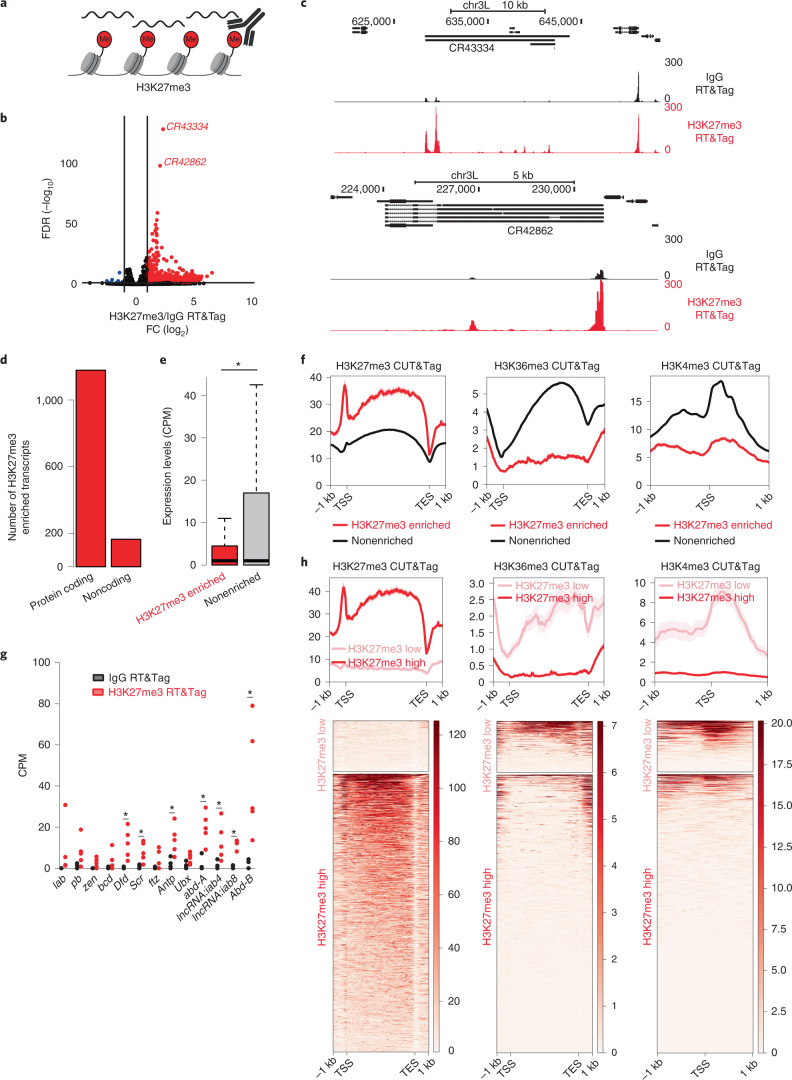
RT&Tag captures transcripts within polycomb domains. **a**, Illustration showing RT&Tag being used to capture transcripts within H3K27me3 demarcated polycomb domains. **b**, Volcano plot showing transcripts that are differentially enriched for H3K27me3 RT&Tag over IgG (FC >2, FDR < 0.05, *n* = 5). Genes enriched for H3K27me3 are highlighted in red, nonenriched are in black and depleted are in blue. The two most highly significant transcripts are labeled. **c**, Genome browser track showing the distribution of H3K27me3 and IgG RT&Tag signal over the gene bodies of *CR43334* and *CR42862*. Combined reads from five replicates are shown. **d**, Bar graph showing the number of H3K27me3-enriched transcripts that are protein coding or noncoding. **e**, Boxplot showing the RNA-seq expression levels (CPM) of H3K27me3-enriched or nonenriched transcripts. *P = 4.32 × 10^−10^, Welch two sample *t*-test (two-sided), *n* = 1,343 for H3K27me3-enriched, *n* = 14,403 for nonenriched, *n* = 2 independent RNA-seq experiments. For the boxplots, the interquartile range (IQR) is shown within the limits of the box, the center line represents the median, the whiskers show data that is within 1.5 times the IQR and outliers are omitted. **f**, Profile plots showing the H3K27me3 (left), H3K36me3 (middle) and H3K4me3 (right) CUT&Tag signal around the gene bodies or TSS of genes that were categorized as being enriched for H3K27me3 RT&Tag or nonenriched. Combined reads from two replicates for H3K27me3 and from one replicate for H3K36me3 and H3K4me3 are shown. Error bands indicate standard error. **g**, Graph showing the IgG and H3K27me3 RT&Tag signal (CPM) for the HOX cluster genes. *FDR < 0.05, *n* = 5 independent RT&Tag experiments. **h**, Profile plots and heatmaps showing the H3K27me3 (left), H3K36me3 (center) and H3K4me3 (right) signal over the gene bodies or TSS of H3K27me3 RT&Tag-enriched transcripts that have high or low levels of H3K27me3 CUT&Tag signal over their gene bodies. Heatmaps are plotted in order of decreasing CUT&Tag signal. Error bands indicate standard error. Source data

**Extended Data Fig. 6:**
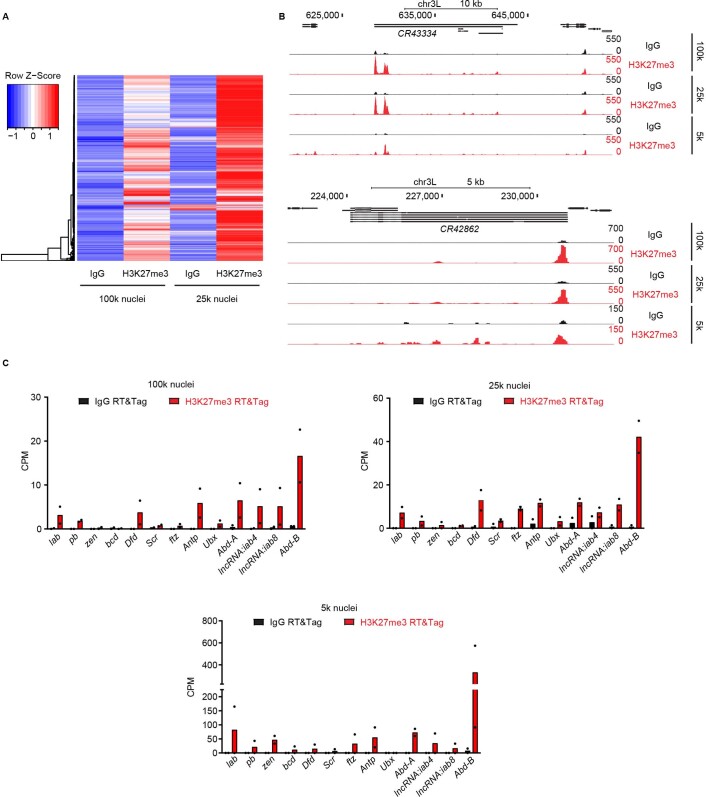
H3K27me3 RT&Tag performance with decreasing number of nuclei input. **a**) Heatmap showing the average IgG and H3K27me3 RT&Tag signal from two experiments performed using either 100,000 or 25,000 nuclei. Individual rows represent the 1342 transcripts identified as H3K27me3-enriched in Fig. 3b. The heatmap colors represent z-score scaling across rows. **b**) Genome browser tracks showing the distribution of IgG and H3K27me3 RT&Tag signal from 100,000, 25,000, or 5000 nuclei over the gene bodies of *CR43334* and *CR42862*. Combined reads from 2 replicates are shown. **c**) Boxplots showing the IgG and H3K27me3 RT&Tag signal (Counts per million, CPM) from 100,000, 25,000, or 5000 nuclei for the HOX cluster genes. *FDR < 0.05, n = 2 independent RT&Tag experiments. Source data

**Extended Data Fig. 7:**
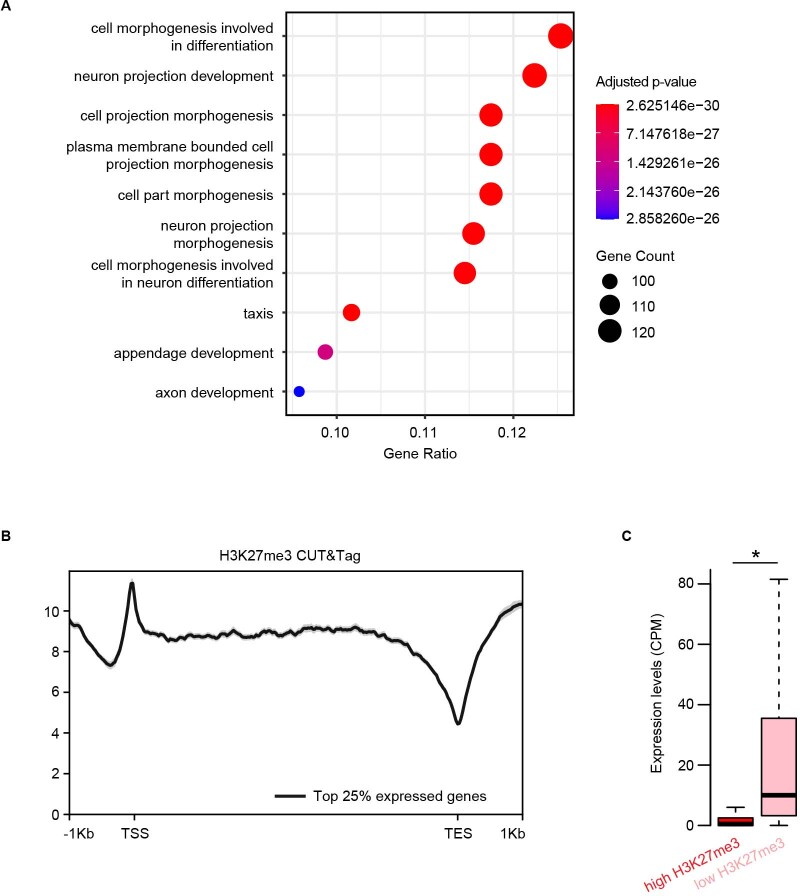
RT&Tag captures transcripts within polycomb domains. **a**) Dot plot showing the top 10 GO biological process terms associated with H3K27me3-enriched transcripts. The dot size corresponds to the gene count and the color represents statistical significance (hypergeometric test, benjamini hochberg p value adjustment). **b**) Profile plot showing the H3K27me3 CUT&Tag signal (combined reads from 2 replicates) over the gene bodies of the top 25% expressed genes. Error bars indicated the standard error. **c**) Boxplot showing the RNA-seq expression levels (Counts per million, CPM) of H3K27me3-RT&Tag enriched transcripts that had either high (>9 read counts) or low (<9 read counts) H3K27me3 CUT&Tag signal over their gene bodies. *p = 0.00488, Welch Two Sample t-test (two-sided), n = 1133 for high H3K27me3, n = 207 for low H3K27me3, n = 2 for RNA-seq. For the boxplots, the IQR is shown within the limits of the box, the center line represents the median, the whiskers show data that is within 1.5 times the IQR, and outliers are omitted. Source data

After validating RT&Tag using MSL2, we applied RT&Tag to identify RNA associated with chromatin domains (Fig. 3a). Polycomb domains are large regions of chromatin decorated with repressive histone H3K27me3 marks^14,15^. They make for an appealing target as studies in mammals have implicated RNA in their establishment and maintenance^15^. Targeting H3K27me3 with an antibody, RT&Tag identified 1,342 transcripts that are differentially enriched for H3K27me3 over IgG background (>2 FC, < 0.05 FDR; Fig. 3b and Supplementary Table 2). As examples, the H3K27me3-targeted RT&Tag signals are shown over the two most statistically significant hits, the lncRNAs *CR43334* and *CR42862* (Fig. 3c). We then assessed the performance of H3K27me3 RT&Tag with decreasing numbers of input nuclei. The H3K27me3 RT&Tag signal was highly reproducible using 100,000 and 25,000 nuclei (Extended Data Fig. 6a) and even 5,000 nuclei for *CR43334* and *CR42862* (Extended Data Fig. 6b). We then proceeded to characterize H3K27me3-enriched transcripts, and found them to be predominantly protein coding (1,178 out of 1,342) with low expression levels (mean 13.5 counts per million (CPM) versus 67.9 CPM for nonenriched genes, *P* = 4.32 × 10^−10^) (Fig. 3d,e). Additionally, H3K27me3 RT&Tag-enriched transcripts had more repressive H3K27me3 CUT&Tag signal and lower active H3K36me3 and H3K4me3 CUT&Tag signal at their TSS or over their gene bodies than nonenriched transcripts (Fig. 3f). In line with this, H3K27me3 RT&Tag-enriched transcripts were characterized by gene ontology (GO) terms for developmental biological processes, which are associated with Polycomb^16^ (Extended Data Fig. 7a). Altogether, these data suggest that H3K27me3 RT&Tag-enriched transcripts are from repressed genes within Polycomb domains. These include classic examples of Polycomb repressed genes such as the Hox genes^17^, which we find show strong enrichment for H3K27me3-targeted RT&Tag signal (Fig. 3g, Extended Data Fig. 6c).

We then assessed what proportion of H3K27me3-targeted RT&Tag transcripts were transcribed from regions decorated by H3K27me3 marks. First, we established the H3K27me3 CUT&Tag background level cut-off in S2 cells as the H3K27me3 CUT&Tag signal over the gene bodies for the top 25% expressed genes (>17 CPM) (Extended Data Fig. 7b). Using this cut-off, 84.5% (1,134 out of 1,342) of H3K27me3-RT&Tag-enriched transcripts were found to be from regions with substantial H3K27me3 CUT&Tag signal (Fig. 3h). These genes also show low levels of active H3K36me3 and H3K4me3 CUT&Tag signal. The remaining 208 H3K27me3-directed RT&Tag-enriched transcripts are from outside of H3K27me3 marked regions and show high H3K36me3 and H3K4me3 CUT&Tag signals. These 208 H3K27me3 RT&Tag-enriched genes are more highly expressed than those from H3K27me3 marked regions (mean 50.1 versus 6.8 CPM, *P* < 0.005; Extended Data Fig. 7c). Given that transcripts captured by RT&Tag must have poly(A) tails, our findings are consistent with the low production of new transcripts from silenced regions, and the subsequent capture of these transcripts near their sites of transcription^18,19^.

### RT&Tag captures transcripts enriched for the m6A modification

**Fig. 4:**
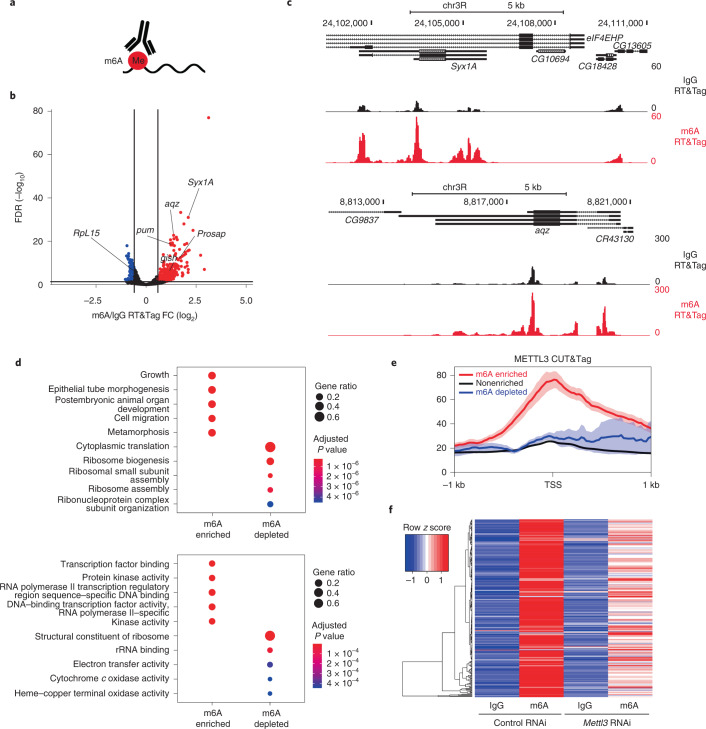
RT&Tag captures transcripts enriched for the m6A modification. **a**, Illustration showing RT&Tag being used to capture transcripts enriched for the m6A posttranscriptional modification. **b**, Volcano plot showing genes that are differentially enriched for m6A over IgG RT&Tag (FC >1.5, FDR < 0.05, *n* = 3). Genes enriched for m6A are highlighted in red, nonenriched are in black and m6A depleted are in blue. Genes previously shown to be enriched or depleted for m6A are labeled. **c**, Genome browser track showing the distribution of m6A and IgG RT&Tag reads over the gene body of *aqz* and *Syx1A*. Combined reads from three replicates are shown. **d**, Dot plot showing the top five GO biological process (top) and molecular function (bottom) terms associated with m6A-enriched and m6A-depleted transcripts. The dot size corresponds to the gene ratio (number of genes related to GO term per total number of m6A-enriched or m6A-depleted genes) and the color represents statistical significance (hypergeometric test, Benjamini–Hochberg *P* value adjustment). **e**, Profile plots showing the METTL3 CUT&Tag signal at the TSS of genes that are enriched, nonenriched or depleted for m6A. Combined reads from three CUT&Tag replicates are shown. Error bands indicate standard error. **f**, Heatmap showing IgG or m6A RT&Tag counts for m6A-enriched genes in S2 cells treated with either control or *Mettl3* RNAi (*n* = 2). The heatmap colors represent *z* score scaling across rows. Source data

**Extended Data Fig. 8:**
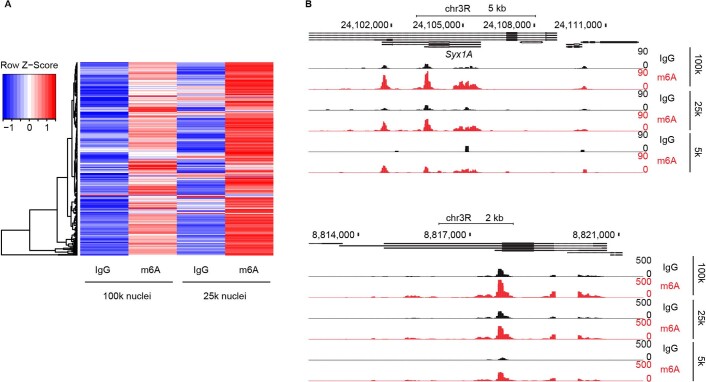
M6A RT&Tag performance with decreasing number of nuclei input. **a**) Heatmap showing the average IgG and m6A RT&Tag signal from two experiments performed using either 100,000 or 25,000 nuclei. Individual rows represent the 281 transcripts identified as m6A-enriched in Fig. 4b. The heatmap colors represent z-score scaling across rows. **b**) Genome browser tracks showing the distribution of IgG and m6A RT&Tag signal from 100,000, 25,000, or 5000 nuclei over the gene bodies of *aqz* and *Syx1A*. Combined reads from 2 replicates are shown. Source data

Having demonstrated that RT&Tag can detect RNAs in protein complexes and chromatin domains, we tested whether our method could be used for RNA modifications. m6A is the most abundant mRNA posttranscriptional modification and has been implicated in numerous aspects of RNA metabolism^20^. Commercial antibodies targeting m6A are available and have been used in RNA immunoprecipitation-based methods (MeRIP-seq and m6A-seq)^7,8^. Although these techniques are valuable for pinpointing the location of m6A modifications, they require large amounts of input material and suffer from low reproducibility^21^. We reasoned that RT&Tag could provide insights into whether a particular transcript is enriched or depleted for m6A relative to IgG control (Fig. 4a). Using RT&Tag, we identified 281 transcripts enriched for m6A (>1.5 FC, < 0.05 FDR) and 106 transcripts depleted for this modification (>1.5 FC, < 0.05 FDR; Fig. 4b and Supplementary Table 3). Of these, *aqz*, *Syx1A*, *gish*, *pum* and *Prosap* transcripts have been previously reported as enriched for m6A^22^ (Fig. 4b,c). Next, we assessed the performance of m6A RT&Tag with varying numbers of input nuclei. The m6A RT&Tag signal was highly reproducible using 100,000 and 25,000 nuclei (Extended Data Fig. 8a) and even 5,000 nuclei for *aqz* and *Syx1A* (Extended Data Fig. 8b). Transcripts enriched for m6A are associated with development and transcription factor binding GO terms, whereas transcripts depleted for m6A tend to be associated with housekeeping GO terms, especially translational components and processes (Fig. 4d).

**Extended Data Fig. 9:**
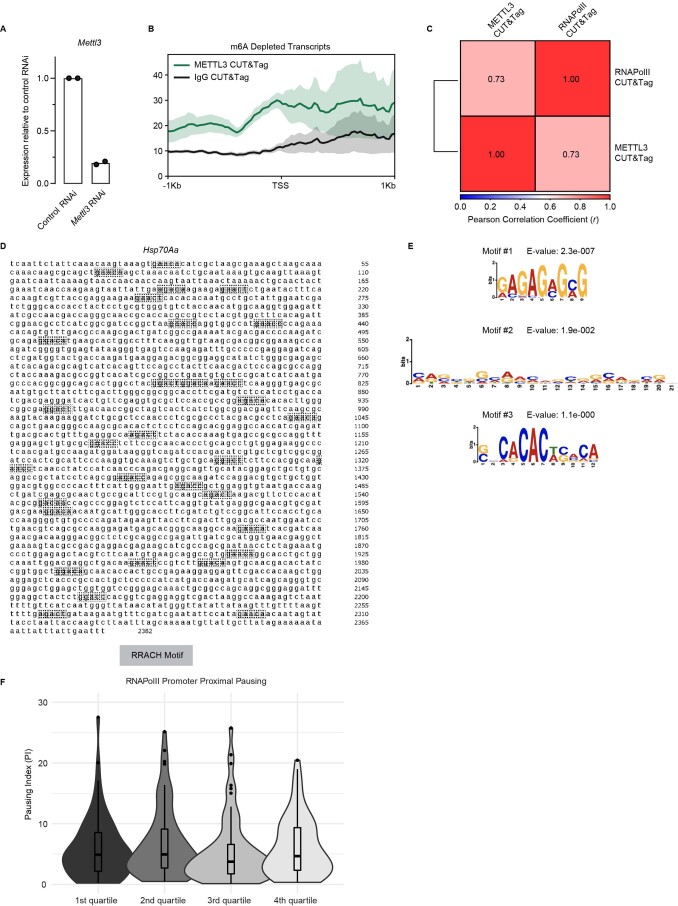
Genes of methylated transcripts are characterized by promoter proximally paused RNA Polymerase II. **a**) Bar plot showing *Mettl3* expression measured by real time PCR in control RNAi and *Mettl3* RNAi S2 cells. Data is plotted relative to control RNAi, n = 2. **b**) Profile plot showing IgG and METTL3 CUT&Tag signal (combined reads from 3 replicates) over the gene bodies of m6A depleted genes. Error bars indicate the standard error. **c**) Pearson correlation between RNAPolII and METTL3 CUT&Tag signal at the promoters of top 25% expressed genes. Combined reads from 2 CUT&Tag replicates were used. **d**) Sequence of *Hsp70Aa* with RRACH motifs highlighted in gray. **e**) MEME motif logos found to be enriched within the promoters of m6A enriched transcripts relative to those of m6A depleted transcripts using the differential enrichment mode setting. **f**) Violin plots displaying the promoter proximal pausing index (PI) m6A-enriched transcripts broken down into quartiles based on their RNA-seq expression levels. PI was calculated by dividing the promoter (+/− 250 bp around the TSS) RNAPolII CUT&Tag signal over the gene body RNAPolII CUT&Tag signal. Welch Two Sample t-test (two-sided), n = 71 for 1^st^ quartile, n = 70 for 2^nd^ quartile, n = 68 for 3^rd^ quartile, n = 72 for 4^th^ quartile, n = 2 for RNAPolII CUT&Tag. For the boxplots drawn within the violin, the IQR is shown within the limits of the box, the center line represents the median, the whiskers show data that is within 1.5 times the IQR, and outliers are omitted. Source data

The *Drosophila* homolog of the METTL3 methyltransferase binds to chromatin and catalyzes the m6A modification on nascent transcripts^23^. We observed high levels of METTL3 CUT&Tag signal at the TSSs of m6A-enriched genes, relative to nonenriched or m6A-depleted genes (Fig. 4e). To validate our list of m6A-enriched genes, we knocked down the gene encoding METTL3 (*Mettl3*, formerly called Inducer of meiosis in yeast or *Ime4*) levels by 80% using RNAi (Extended Data Fig. 9a). Doing so resulted in a modest decrease (>10%) for 81% of m6A-enriched transcripts (Fig. 4f). Altogether, these results show that m6A-enriched transcripts identified by RT&Tag are METTL3 methylation dependent.

### Promoters of m6A transcripts have paused RNAPolII

**Fig. 5:**
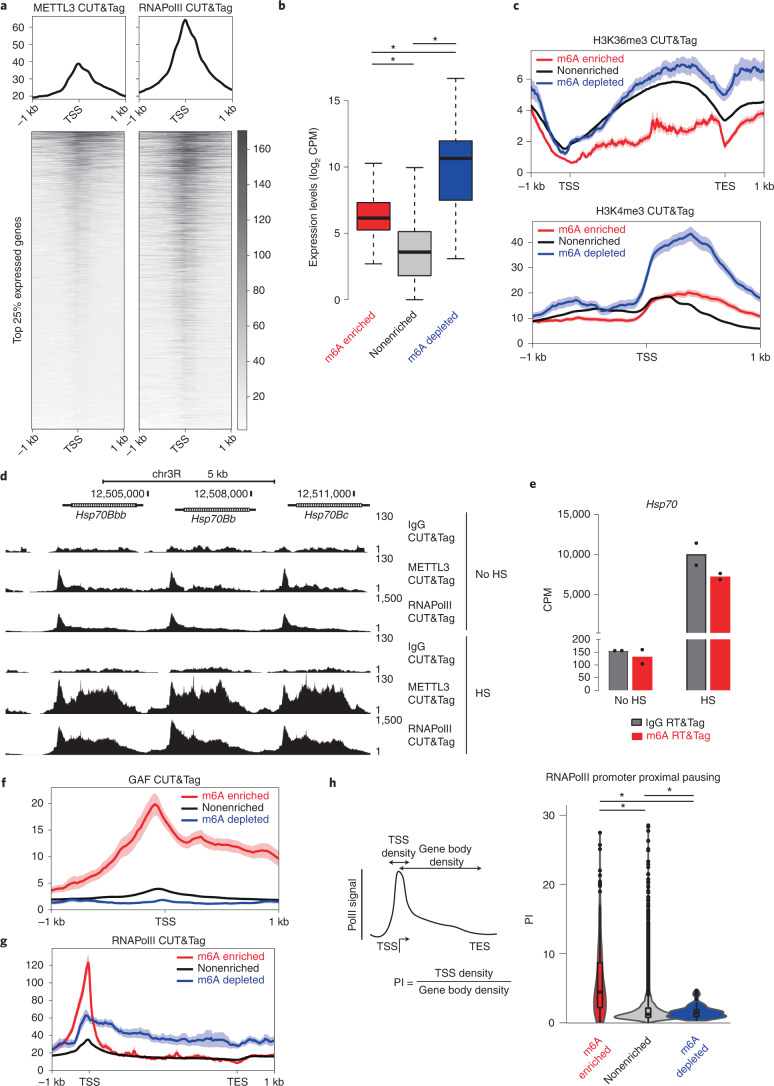
Promoters of m6A transcripts have paused RNAPolII. **a**, Profile plots and heatmaps showing METTL3 (left) and total RNAPolII (right) CUT&Tag signal at the TSS of the top 25% expressed genes. Combined reads from two CUT&Tag replicates are used. Heatmaps are plotted in the order of decreasing METTL3 signal. **b**, Boxplot showing the RNA-seq expression levels (CPM) of genes that are depleted, enriched or nonenriched for m6A on a log_2_ scale. *P = 0.001265 (m6A enriched versus depleted), *P = 2.979 × 10^−8^ (m6A enriched versus nonenriched) and *P = 0.0008931 (m6A depleted versus nonenriched), Welch two sample *t*-test (two-sided), *n* = 281 for m6A enriched, *n* = 106 for m6A depleted and *n* = 12,129 for nonenriched from *n* = 2 independent RNA-seq experiments. The IQR is shown within the limits of the box, the center line represents the median, the whiskers show data that is within 1.5 times the IQR and outliers are omitted. **c**, Profile plots showing the H3K36me3 (top) and H3K4me3 (bottom) CUT&Tag signal over the gene bodies or at the TSS of genes that are enriched, nonenriched or depleted for m6A. Reads from one CUT&Tag replicate are shown. Error bands indicate standard error. **d**, Genome browser tracks showing the IgG, METTL3 and RNAPolII CUT&Tag signal over the gene bodies of *Hsp70* genes with no heat shock (HS) or after 15 min of HS. Combined reads from two replicates are shown. **e**, Bar graph showing the IgG and m6A RT&Tag signal for *Hsp70* with no HS and after 15 min of HS, *n* = 2. **f**, Profile plot showing GAGA factor (GAF) CUT&Tag signal at the TSS of m6A-enriched, nonenriched and depleted transcripts. Reads from one CUT&Tag replicate are shown. **g**, Profile plot showing RNAPolII CUT&Tag signal over the gene bodies of m6A-enriched, nonenriched and depleted transcripts. Reads from two CUT&Tag replicates are shown. Error bands in **f** and **g** indicate standard error. **h**, Schematic showing how the promoter proximal PI was calculated (left). Violin plots displaying the PI of m6A-enriched, nonenriched and depleted transcripts (right). *P < 2.2 × 10^−16^ (m6A enriched versus depleted), *P* < 2.2 × 10^−16^ (m6A enriched versus nonenriched), *P* = 0.0009435 (m6A depleted versus nonenriched), Welch two sample *t*-test (two-sided), *n* = 281 for m6A enriched, *n* = 106 for m6A depleted and *n* = 12,129 for nonenriched, *n* = 2 for RNAPolII CUT&Tag. For the boxplots drawn within the violin, the IQR is shown within the limits of the box, the center line represents the median, the whiskers show data that is within 1.5 times the IQR, and outliers are omitted. Source data

Whereas the promoters of genes producing m6A-enriched transcripts are enriched for METTL3, we noticed that the METTL3 CUT&Tag signal at TSSs of m6A-depleted transcripts was still above IgG CUT&Tag signal (Extended Data Fig. 9b). In fact, METTL3 binding was widely observed amongst the top 25% expressed genes (>17 CPM) (Fig. 5a). Indeed, total RNAPolII and METTL3 binding are positively correlated (Fig. 5a and Extended Data Fig. 9c)^24,25^. Thus, we reasoned that METTL3 must be preferentially recruited to sites of active transcription. This leads to the expectation that highly expressed transcripts would be enriched for transcript methylation. However, m6A-enriched transcripts tend to be expressed at lower levels than m6A-depleted transcripts (154 CPM versus 3478 CPM, *P* = 0.001265; Fig. 5b). In line with expression level differences, genes producing m6A-enriched transcripts have lower levels of active H3K4me3 and H3K36me3 marks (Fig. 5c). Hence, the m6A methylation mark is not associated with high levels of transcription. We then asked whether increasing METTL3 levels at a gene would in turn result in more transcript methylation. Heat shock (HS) of Drosophila cells induces a large influx of RNAPolII into the bodies of HS protein (HSP) genes^26^, which we can observe by CUT&Tag (Fig. 5d). In addition to RNAPolII enrichment, we found that HS causes a dramatic increase in METTL3 (Fig. 5d). This increase is not limited to promoters, but now extends into the bodies of the *Hsp70* genes. However, induced *Hsp70* transcripts do not accumulate the m6A modification, despite the large influx of METTL3 and presence of RRACH motifs (the RNA sequence in which the m6A modification occurs) within the *Hsp70* transcripts (Fig. 5e and Extended Data Fig. 9d). Thus, METTL3 binding on its own does not reliably predict methylation status.

What other features might distinguish m6A-enriched and m6A-depleted transcripts? Motif analysis revealed GAGA motifs within the promoters of m6A-enriched transcripts (Extended Data Fig. 9e). GAGA factor (GAF) is a DNA-binding transcription factor that binds GAGA motifs and is associated with promoter proximal pausing of RNAPolII^27^. In line with GAGA motif enrichment, much higher GAF CUT&Tag signal is detected at the TSSs of m6A-enriched genes (Fig. 5f). For this reason, we looked at the distribution of total RNAPolII signal over gene bodies relative to the TSS. We observed m6A-enriched transcripts to have more RNAPolII signal at the TSS and less within gene bodies (Fig. 5g). We then calculated the RNAPolII promoter proximal pausing index (PI) as the ratio of RNAPolII signal at the promoter (±250 bp around the TSS) to signal over the gene body. Indeed, m6A-enriched transcripts had very high levels of PI relative to m6A-depleted transcripts (6.3 versus 1.9, *P* < 2.2 × 10^–16^) (Fig. 5h). This high level of PI was not related to the expression level of the m6A-enriched transcripts (Extended Data Fig. 9f). Altogether, our findings suggest that transcripts with a very high degree of polymerase pausing and high GAF binding at their promoters are predominantly enriched for the m6A posttranscriptional modification.

## Discussion

In this work we developed RT&Tag, a proximity labeling tool, that uses antibodies to tether Tn5 and tagment nearby RNA within intact nuclei. RT&Tag differs from immunoprecipitation-based methods, which capture RNA binding to factors within a cell lysate instead of endogenous proximity interactions. Furthermore, RT&Tag does not require cross-linking or RNA fragmentation, and the same RT&Tag protocol can be applied to RNA–protein interactions, RNA–chromatin interactions and RNA modifications. In contrast, immunoprecipitation techniques require separate protocols for each application.

A main advantage of RT&Tag over immunoprecipitation is its efficiency. RT&Tag requires fewer than ~100,000 cells, which is at least 50-fold fewer than the number needed for PIRCh-seq and ChRIP-seq (Table 1)^5,6^. RT&Tag can work with fewer sequencing reads as the RT&Tag reads are concentrated at the 3′ end of RNA^28^. Specifically, we have had success with 4–8 million reads per sample for RT&Tag, relative to PIRCh-seq where around 50 million reads were used (Table 1)^5^. Other enzyme-tethering based techniques are emerging as in situ alternatives to immunoprecipitation. For example, APEX sequencing (APEX-seq) and targets of RBPs identified by editing (TRIBE) tether RNA modifying enzymes by fusing them with other proteins^29–31^. However, these methods have yet to be used to identify RNA interactions occurring on chromatin. Additionally, the need to generate fusion proteins for each protein target makes these techniques laborious and low throughput, unlike RT&Tag, which can be easily applied to any epitope with an available antibody. Another advantage of RT&Tag is that RNA/cDNA hybrids are directly tagmented by Tn5 with sequencing adapters. This allows for seamless generation of Illumina sequencing libraries using a simple PCR reaction, without the need to purify RNA as in ChRIP-seq, APEX-seq and TRIBE. The lack of purification steps makes RT&Tag adaptable for automation as was done with AutoCUT&Tag^32^. Together with low cell number input and low sequencing depth, RT&Tag presents a high-throughput method to study RNA metabolism by targeting chromatin factors and posttranslational modifications.

**Table 1:** Comparison of RT&Tag with immunoprecipitation-based methods

		RNA–protein		RNA–chromatin		RNA modification	
	RT&Tag	RIP-seq	CLIP-seq	PIRCh-seq	ChRIP-seq	MeRIP-seq	m6A-RIP-seq
**Input material**	0.1 million	20 million	20 million	5 million	20 million	20 million	50 million
**Sequencing reads**	4–8 million	20 million	25 million	50 million	50 million	73 million	20 million
**Time**	1–2 days	3 days	4 days	3 days	3 days	2–3 days	2–3 days
**Cost**	$50	$150	$200	$225	$200	$300	$200

Using RT&Tag, we gained insight into the N6-methyladenosine (m6A) modification. m6A is the most prevalent mRNA posttranscriptional modification and has been implicated in splicing, mRNA decay and translation^20^. The m6A modification is catalyzed by the methyltransferase, METTL3 (ref. ^23^). How METTL3 discriminates which RNAs get methylated is unclear. We have observed widespread METTL3 binding at the promoters of expressed genes. However, we found that most of these genes were not enriched for m6A, suggesting that other factors must be involved. Instead, we found RNAPolII promoter pausing to be a strong predictor of m6A deposition. We were surprised that *Hsp70*, a gene known to exhibit RNAPolII pausing, was not identified as being m6A-enriched using RT&Tag. However, upon calculating the pausing index of *Hsp70*, we have found it to be on par with that of m6A nonenriched transcripts. This suggests that only genes exhibiting very high levels of RNAPolII pausing are enriched for m6A. RNAPolII dynamics, especially elongation speed, have previously been implicated in regulating cotranscriptional processes including splicing and alternative polyadenylation^33^. Furthermore, human MCF7 breast cancer cells expressing a slow elongation RNAPolII mutant have been reported to have increased m6A levels^34^. How RNAPolII promoter pausing contributes to m6A deposition is not known but may be due to the increased amount of time METTL3 is bound near the promoter. As such, METTL3 would have more contact time with the 5′ end of RNA, the region where m6A is predominantly found in *Drosophila*^35^. METTL3 itself has been found to promote productive RNAPolII elongation, which suggests that there may be two-way communication between m6A and RNAPolII processivity^25,36^. An alternative explanation for the discrepancy between METTL3 binding and m6A levels is that methylation may occur at all METTL3-bound transcripts but not be retained. Fat mass and obesity-associated protein (FTO) is a demethylase that is known to remove the m6A mark after transcription in mammals^20^. However, no FTO homolog has been identified in *Drosophila*^23^. Deposition of m6A at splice junctions and introns of nascent transcripts has been implicated in regulating splicing^37^. Thus, intronic m6A marks may be lost during splicing and not be captured by m6A RT&Tag, which specifically measures m6A levels in mature transcripts. Altogether, our findings suggest METTL3 binding does not correspond to the presence of m6A, and that additional factors are necessary for transcript methylation.

RT&Tag could have numerous applications given an available antibody. Although this work described only chromatin applications, RT&Tag is not necessarily limited to chromatin, and future studies might adapt RT&Tag for targets in the cytoplasm, such as RNA–protein interactions. Efforts to catalog RBP-bound transcripts are still in their infancy. Phase 3 of the ENCODE consortium profiled 150 RBPs using immunoprecipitation in HepG2 and K562 cell lines^38^. Given that the human genome contains over 1,500 RBP-encoding genes and mutations in RBPs are becoming implicated in genetic diseases, much work remains to be done to characterize their bound transcripts^39,40^. Similarly, cataloging sites of m6A modification on a large scale is yet to be done. METTL3 knockout experiments in mammals (humans and mice) have shown that m6A is required for cell differentiation and embryonic viability^41–44^. The commonly used MeRIP-seq and m6A-seq techniques require large amounts of RNA input, which makes them impractical for studying differentiating cells and development. RT&Tag can fill the need for high-throughput profiling of chromatin-bound, RBP-RNA interactions and m6A-enriched transcripts, especially when sample input is limiting such as with clinical samples or embryonic cells.

## Methods

### Cell culture and nuclei preparation

Drosophila S2 cells were obtained from Invitrogen (10831-014) and cultured in HyClone SFX-Insect cell culture medium (HyClone) supplemented with 18 mM l-glutamine (Sigma-Aldrich). S2 cells were maintained at the confluency of 2–10 million cells ml^–1^ at 25 °C. To induce the HS response, S2 cells were placed at 37 °C for 15 min. To prepare nuclei for CUT&Tag and RT&Tag, 4 million S2 cells were collected by centrifuging at 300*g* for 5 min followed by a wash with 1× PBS. Nuclei were then isolated by incubating with NE1 buffer (10 mM HEPES pH 7.9, 10 mM KCl, 0.1% Triton X-100, 20% glycerol, 0.5 mM spermidine, Roche Complete Protease Inhibitor Cocktail) for 10 min on ice. The nuclei were then centrifuged at 500*g* for 8 min and resuspended in wash buffer (20 mM HEPES pH 7.5, 150 mM NaCl, 0.5 mM spermidine, Roche Complete Protease Inhibitor Cocktail). The nuclei were either used fresh or were frozen in wash buffer with 10% DMSO and stored at −80 °C. For RT&Tag, the NE1 and wash buffers were supplemented with 1 U μl^–1^ of RNasin Ribonuclease Inhibitor (Promega).

### Antibodies

The following primary antibodies were used for RT&Tag and CUT&Tag experiments: rabbit anti-IgG (Abcam, catalog no. ab172730), rabbit anti-MSL2 (gift from M. Kuroda, Harvard Medical School), rabbit anti-H4K16ac (Abcam, catalog no. ab109463), rabbit anti-H3K27me3 (Cell Signaling Technology, catalog no. CST9733), rabbit anti-H3K36me3 (Thermo, catalog no. MA5-24687), rabbit anti-H3K4me3 (Thermo, catalog no. 711958), rabbit anti-m6A (Megabase, catalog no. AP60500), rabbit anti-METTL3 (Proteintech, catalog no. 15073-1-AP), mouse anti-unphosphorylated RNA polymerase II (Abcam ab817) and rabbit anti-GAF (gift from G. Cavalli, CNRS Montpellier France). The following secondary antibodies were used: guinea pig anti-rabbit (Antibodies Online, catalog no. ABIN101961) and rabbit anti-mouse (Abcam, catalog no. ab46450). Streptavidin-conjugated secondary antibodies were generated using the Streptavidin Conjugation Kit (Abcam, catalog no. ab102921) as per the manufacturer’s instructions.

### RT&Tag

The step-by-step protocol can be accessed at https://www.protocols.io/view/rt-amp-tag-bn36mgre. Single-loaded pA-Tn5 was assembled before starting RT&Tag. First, the Mosaic end- adapter A (ME-A) and its reverse (ME-Rev) oligonucleotides were annealed in annealing buffer (10 mM Tris pH 8, 50 mM NaCl, 1 mM EDTA) by heating them at 95 °C for 5 min and slowly allowing them to cool to room temperature (Supplementary Table 4). Afterwards, 16 µl of 100 µM annealed ME-A were mixed with 100 µl of 5.5 µM pA-Tn5 for 1 h at room temperature and stored at −20 °C for future use. S2 nuclei were isolated and bound to paramagnetic Concanavalin A (ConA) beads (Bangs Laboratories). To do so, ConA beads were first activated via two washes with binding buffer (10 mM HEPES pH 7.9, 10 mM KCl, 1 mM CaCl_2_, 1 mM MnCl_2_). Afterwards, 100,000 S2 nuclei were bound to 5 μl of ConA beads for 10 min at room temperature. The ConA bound nuclei were then incubated with primary antibody diluted 1:100 in antibody buffer (20 mM HEPES pH 7.5, 150 mM NaCl, 0.5 mM spermidine, Roche Complete Protease Inhibitor Cocktail, 2 mM EDTA, 0.1% BSA and 1 U μl^–1^ RNasin ribonuclease inhibitor) at 4 °C overnight. Afterwards, nuclei were incubated with streptavidin-conjugated secondary antibody diluted 1:100 in wash buffer (20 mM HEPES pH 7.5, 150 mM NaCl, 0.5 mM spermidine, Roche Complete Protease Inhibitor Cocktail) for 45 min at room temperature. Two rounds of washes with wash buffer were then performed and nuclei were incubated with 0.2 mM biotinylated oligo(dT)-ME-B in wash buffer for 20 min at RT. Two rounds of washes with wash buffer were then performed and nuclei were incubated with ME-A loaded pA-Tn5 diluted 1:200 in 300 wash buffer (20 mM HEPES pH 7.5, 300 mM NaCl, 0.5 mM spermidine, Roche Complete Protease Inhibitor Cocktail, and 1 U μl^–1^ RNasin ribonuclease inhibitor) for 1 h at room temperature. ConA bound nuclei were then washed three times with 300 wash buffer. Simultaneous RT and tagmentation were then performed by resuspending nuclei in MgCl_2_ containing RT (1× Maxima RT buffer contains 50 mM Tris-HCl pH 8.3, 75 mM KCl, 3 mM MgCl_2_, 10 mM DTT along with, 0.5 mM dNTPs, 10 U μl^–1^ of Maxima H minus reverse transcriptase, and 1 U μl^–1^ of RNasin ribonuclease inhibitor) for 2 h at 37 °C. The nuclei were then washed with 10 mM TAPS and pA-Tn5 was stripped off by resuspending nuclei in 5 μl stripping buffer (10 mM TAPS with 0.1% SDS) and incubating for 1 h at 58 °C. Libraries were then generated using PCR. The nuclei suspension was mixed with 15 μl 0.67% Triton X-100, 2 μl 10 mM i7 primer, 2 μl 10 mM i5 primer and 25 μl 2× NEBNext Master Mix (NEB). The following PCR conditions were used: (1) 58 °C for 5 min, (2) 72 °C for 5 min, (3) 98 °C for 30 s, (4) 98 °C for 10 s, (5) 60 °C for 15 s, (6) repeat steps (4)–(5) 13 times, (7) 72 °C for 2 min, (8) hold at 4 °C. Sequencing libraries were then purified using 0.8× HighPrep PCR Cleanup System (MagBio) beads as per the manufacturer’s instructions. Libraries were then resuspended in 21 μl 10 mM Tris- HCl pH 8. Library concentrations were quantified using the High Sensitivity D5000 TapeStation system (Agilent).

### CUT&Tag

CUT&Tag was carried out as described previously (https://www.protocols.io/view/cut-amp-tag-direct-with-cutac-x54v9mkmzg3e/v3)^10^. Briefly, S2 nuclei were bound to ConA beads at the ratio of 100,000 nuclei per 5 μl beads for 10 min at room temperature. Nuclei were then incubated with primary antibody (1:100) at 4 °C overnight followed by secondary antibody (1:100) for 45 min at room temperature the next day. Excess antibody was removed via two rounds of washes, and the nuclei were incubated with loaded pA-Tn5 (1:200) for 1 h at RT. Nuclei were washed three times to remove excess pA-Tn5 and then MgCl_2_ was added to perform tagmentation for 1 h at 37 °C. The reaction was then stopped by doing a wash with 10 mM TAPS and stripping off pA-Tn5 by resuspending nuclei in 0.1% SDS buffer and incubating for 1 h at 58 °C. The SDS was then neutralized with Triton X-100 and libraries were amplified with NEBNext Master Mix (NEB) using 12 rounds of amplification. Sequencing libraries were then purified using 1.2× ratio of HighPrep PCR Cleanup System (MagBio) as per manufacturer’s instructions. Libraries were then resuspended in 21 μl 10 mM Tris-HCl pH 8. Library concentrations were quantified using the D1000 TapeStation system (Agilent).

### RNA interference

PCR templates for in vitro transcription (IVT) were amplified from S2 cell cDNA or pGFP5(S65T) plasmid using Phusion Hot Start Flex DNA Polymerase (NEB) and primers listed in Supplementary Table 5. PCR products were purified using NucleoSpin Gel and PCR Clean-Up Kit (Clontech). IVT was performed to generate dsRNA using the T7 High Yield RNA Synthesis Kit (NEB). Template DNA was removed using Turbo DNAse (Ambion) and dsRNA was purified using the NucleoSpin RNA Clean up XS kit (Clontech). To perform RNA interference (RNAi), S2 cells were seeded at a density of 1 million cells ml^–1^ of serum-free medium. As control RNAi, a total of 30 μg green fluorescent protein (GFP) dsRNA was added to cells. For *Mettl3* RNAi, 15 μg *Mettl3* dsRNA number 1 plus 15 μg *Mettl3* dsRNA number 2 were added. After 6 h, medium was replaced with serum containing medium. Treatment with dsRNA was repeated after 48 and 96 h. Cells were collected after 120 h.

### RT-qPCR

Total RNA was extracted from S2 cells using the RNeasy Plus Mini Kit (Qiagen) according to the manufacturer’s instructions. cDNA was synthesized using the Maxima H Minus Reverse Transcriptase (Thermo Scientific). Real time PCR was performed with the Maxima SYBR Green qPCR Master Mix (Thermo Scientific) using the ABI QuantStudio5 Real Time PCR Systems instrument. Primers used are listed in Supplementary Table 6. Gene expression levels were quantified using the delta delta Ct method using ribosomal protein L32 (RPL32) gene for normalization.

### RNA-sequencing

Total RNA from S2 cells was isolated using the RNeasy Plus Mini Kit (Qiagen). Maxima H Minus Reverse Transcriptase (Thermo Fisher Scientific) was used as per the manufacturer’s instructions for first-strand synthesis. RT was primed using the oligo(dT)- ME-B fusion oligonucleotide. Tagmentation was then performed using 100 ng RNA-cDNA hybrids, ME-A loaded pA-Tn5 and tagmentation buffer (20 mM HEPES pH 7.5, 150 mM NaCl, 10 mM MgCl_2_) for 1 h at 37 °C. Tagmented RNA-cDNA hybrids were purified using 1× ratio of HighPrep PCR Cleanup System (MagBio) as per the manufacturer’s instructions. Sequencing libraries were then amplified using NEBNext Master Mix (NEB) using 12 cycles. Libraries were then purified using 0.8× ratio of HighPrep PCR Cleanup System (MagBio) as per the manufacturer’s instructions. Libraries were then resuspended in 21 μl 10 mM Tris-HCl pH 8 and quantified using the D5000 TapeStation system (Agilent).

### Sequencing and data preprocessing

For RT&Tag and RNA-sequencing, single-end 50 base pair (bp) sequencing was performed on the Illumina HiSeq. The sequencing reads were aligned using HISAT2 (v.2.1.0) to the UCSC dm6 genome with the options:–max-intronlen 5000–rna-strandness F^45^. The aligned reads were then quantified using Subread (v.2.0.0) featureCounts with the Ensembl dm6 gene annotation file using the following options: -s 1 -t exon -g gene_id^46^. HISAT2 alignment statistics, PCR duplication rate (Samtools v.1.11 markdup)^47^ and number of detected transcripts are included in Supplementary Table 7. Differential expression and PCA were performed using DESeq2 (v.1.32.0)^48^. The genomic origin of RT&Tag reads was determined using QualiMap (v.2.2.2) RNA-Seq QC^49^. IgG normalized MSL2 RT&Tag signal was visualized over the *Drosophila* chromosomes using karyoploteR (v.1.18.0)^50^. GO term enrichment analysis for H3K27me3 and m6A-enriched or m6A-depleted transcripts was performed using clusterProfiler (v.4.0.5)^51^ and org.Dm.eg.db (v.3.13.0)^52^. The distribution of RT&Tag reads across the gene bodies of *Drosophila* genes was calculated using RSeQC (v.2.6.4)^53^. For CUT&Tag, paired-end 25 bp sequencing was performed on the Illumina HiSeq and data were analyzed as described (https://www.protocols.io/view/cut-amp-tag-data-processing-and-analysis-tutorial-e6nvw93x7gmk/v1)^10^ using Bowtie2 (v.2.4.2)^54^. MSL2 and H3K27me3 peaks were called using SEACR (v.1.3) using the norm setting^55^. Profile plots, heatmaps and correlation matrices were generated using deepTools (v.3.5.1)^56^. RRACH motifs were identified using the FIMO tool from the MEME (v.5.3.3) suit^57^. Motif enrichment within the promoters of m6A-enriched versus m6A-depleted transcripts was performed using the MEME tool from the MEME (v.5.3.3) suit using the differential enrichment mode^58^. Genome browser screenshots were obtained from the University of California Santa Cruz (UCSC) Genome Browser. Graphs were plotted using R Studio (v.4.1.1) (https://www.r-project.org) using base graphics or using packages including gplots (v.3.1.3)^59^, ggplot2 (v.3.3.6) (https://ggplot2.tidyverse.org), ggrepel (v.0.9.1)^60^, VennDiagram (v.1.7.3)^61^, viridis (v.0.6.2)^62^ and hrbrthemes (v.0.8.0)^63^. Other R packages used for analysis included tidyverse (v.1.3.1)^64^, GenomicRanges (v.1.44.0)^65^ and rtracklayer (v.1.52.1)^66^. Art schematics in Fig. 1a-b, Fig. 2a, Fig. 3a, and Fig. 4a were created with BioRender.com.

### Reporting summary

Further information on research design is available in the Nature Research Reporting Summary linked to this article.

## Online content

Any methods, additional references, Nature Research reporting summaries, source data, extended data, supplementary information, acknowledgements, peer review information; details of author contributions and competing interests; and statements of data and code availability are available at 10.1038/s41592-022-01618-9.

## Supplementary information

Reporting Summary

Peer Review File

Supplementary Tables 1–7 Table 1. MSL2 RT&Tag differentially enriched transcripts; Table 2. H3K27me3 RT&Tag differentially enriched transcripts; Table 3. M6A RT&Tag differentially enriched transcripts; Table 4. RT&Tag oligonucleotides; Table 5. RNAi construct primers; Table 6. Real time-PCR primers; Table 7. RT&Tag data quality statistics.

## Data Availability

All primary sequencing data have been deposited as single-end or paired-end fastq files in the Gene Expression Omnibus under accession code GSE195654. The dm6 genome from UCSC (https://hgdownload.soe.ucsc.edu/goldenPath/dm6/bigZips/) was used for genome alignment and Drosophila_melanogaster.BDGP6.28.47.gtf file (http://ftp.ensembl.org/pub/release-102/gtf/drosophila_melanogaster/) was used for generating transcript count tables. Source data are provided with this paper.

## Source data

Source Data Fig. 2 Statistical source data.
Source Data Fig. 3 Statistical source data.
Source Data Fig. 4 Statistical source data.
Source Data Fig. 5 Statistical source data.
Source Data Extended Data Fig. 1 Statistical source data.
Source Data Extended Data Fig. 3 Statistical source data.
Source Data Extended Data Fig. 4 Statistical source data.
Source Data Extended Data Fig. 5 Statistical source data.
Source Data Extended Data Fig. 6 Statistical source data.
Source Data Extended Data Fig. 7 Statistical source data.
Source Data Extended Data Fig. 8 Statistical source data.
Source Data Extended Data Fig. 9 Statistical source data.

## Extended data

are available for this paper at 10.1038/s41592-022-01618-9.

## Code availability

Custom code for identifying and analyzing RT&Tag-enriched transcripts is available at https://github.com/nadiyakhyzha/RTTag_Analysis.
