# Supplementary material for: Profiling RNA at chromatin targets in situ by antibody-targeted tagmentation
Source: Nat Methods. 2022 Oct 3;19(11):1383–92. doi: 10.1038/s41592-022-01618-9 (PMC9636022; doi:10.1038/s41592-022-01618-9)
Supplement: Supplementary file 1 — Reporting Summary [file 41592_2022_1618_MOESM1_ESM.pdf]

nature research

Corresponding author(s): Steven Henikoff and Kami Ahmad

Last updated by author(s): Jul 22, 2022

Reporting Summary

Nature Research wishes to improve the reproducibility of the work that we publish. This form provides structure for consistency and transparency in reporting. For further information on Nature Research policies, see our Editorial Policies and the Editorial Policy Checklist.

Statistics

For all statistical analyses, confirm that the following items are present in the figure legend, table legend, main text, or Methods section.

n/a	Confirmed	
☐	☒	The exact sample size (*n*) for each experimental group/condition, given as a discrete number and unit of measurement
☒	☐	A statement on whether measurements were taken from distinct samples or whether the same sample was measured repeatedly
☐	☒	The statistical test(s) used AND whether they are one- or two-sided<br>*Only common tests should be described solely by name; describe more complex techniques in the Methods section.*
☒	☐	A description of all covariates tested
☒	☐	A description of any assumptions or corrections, such as tests of normality and adjustment for multiple comparisons
☐	☒	A full description of the statistical parameters including central tendency (e.g. means) or other basic estimates (e.g. regression coefficient) AND variation (e.g. standard deviation) or associated estimates of uncertainty (e.g. confidence intervals)
☐	☒	For null hypothesis testing, the test statistic (e.g. F, t, r) with confidence intervals, effect sizes, degrees of freedom and P value noted<br>*Give P values as exact values whenever suitable.*
☒	☐	For Bayesian analysis, information on the choice of priors and Markov chain Monte Carlo settings
☒	☐	For hierarchical and complex designs, identification of the appropriate level for tests and full reporting of outcomes
☐	☒	Estimates of effect sizes (e.g. Cohen's *d*, Pearson's *r*), indicating how they were calculated

Our web collection on statistics for biologists contains articles on many of the points above.

Software and code

Policy information about availability of computer code

Data collection	The size distributions and molar concentration of libraries were determined using an Agilent 4200 TapeStation. Up to 96 barcoded RT&Tag and CUT&Tag libraries were pooled at approximately equimolar concentration for sequencing. Single-end 50 bp sequencing (RT&Tag) and paired-end 25×25 bp sequencing (CUT&Tag) on the Illumina HiSeq 2500 platform was performed by the Fred Hutchinson Cancer Research Center Genomics Shared Resources. This yielded 3-5 million reads per antibody. Single-end reads were aligned using HISAT2 version 2.1.0 to UCSC dm6 with options: "--max-intronlen 5000 --rna-strandness F. Paired-end reads were aligned using Bowtie2 version 2.4.2 to UCSC dm6 with options: --end-to-end --very-sensitive --no-mixed --no-discordant -q --phred33 -I 10 -X 700.
Data analysis	FastQC (0.11.7); Trim_Galore (0.6.5); HISAT2 (2.1.0); Bowtie2 (2.4.2); Subread featureCounts (2.0.0); RSeQC (2.6.4); MEME (5.3.3); Samtools (1.11); SEACR (1.3); Bedtools (2.30.0); Deeptools (3.5.1); Qualimap (2.2.2); R version 4.1.1, R libraries used: rtracklayer (1.52.1), DESeq2 (1.32.0), ggplot2 (3.3.6), ggrepel (0.9.1), GenomicRanges (1.44.0), karyoploteR (1.18.0), gplots (3.1.3), VennDiagram (1.7.3), viridis (0.6.2), tidyverse (1.3.1), hrbrthemes (0.8.0), clusterProfiler (4.0.5), org.Dm.eg.db (3.13.0); Custom Code is uploaded to GitHub which can be accessed with the following link: https://github.com/nadiyakhyzha/RTTag_Analysis.

For manuscripts utilizing custom algorithms or software that are central to the research but not yet described in published literature, software must be made available to editors and reviewers. We strongly encourage code deposition in a community repository (e.g. GitHub). See the Nature Research guidelines for submitting code & software for further information.

Data

Policy information about availability of data

All manuscripts must include a data availability statement. This statement should provide the following information, where applicable:

- Accession codes, unique identifiers, or web links for publicly available datasets
- A list of figures that have associated raw data
- A description of any restrictions on data availability

All primary sequencing data are deposited as single-end (RT&Tag) or paired-end (CUT&Tag) fastq files in Gene Expression Omnibus GSE195654. The dm6 genome from UCSC (https://hgdownload.soe.ucsc.edu/goldenPath/dm6/bigZips/) was used for genome alignment and Drosophila_melanogaster.BDGP6.28.47.gtf file (http://ftp.ensembl.org/pub/release-102/gtf/drosophila_melanogaster/) was used for generating transcript count tables.

Field-specific reporting

Please select the one below that is the best fit for your research. If you are not sure, read the appropriate sections before making your selection.

☒ Life sciences ☐ Behavioural & social sciences ☐ Ecological, evolutionary & environmental sciences

For a reference copy of the document with all sections, see nature.com/documents/nr-reporting-summary-flat.pdf

Life sciences study design

All studies must disclose on these points even when the disclosure is negative.

Sample size	No sample size calculations were performed. At least 2 replicates were performed for all RNA and chromatin profiling experiments in the S2 Drosophila cell line, as is currently acceptable in the field.
Data exclusions	No data was excluded.
Replication	At least 2 replicates were profiled. All attempts at replication were successful.
Randomization	n/a. The data and analysis for this study is objective and not prone to influence by the researchers bias.
Blinding	n/a. The data and analysis for this study is objective and not prone to influence by the researchers bias.

Reporting for specific materials, systems and methods

We require information from authors about some types of materials, experimental systems and methods used in many studies. Here, indicate whether each material, system or method listed is relevant to your study. If you are not sure if a list item applies to your research, read the appropriate section before selecting a response.

Materials & experimental systems

n/a	Involved in the study	
☐	☒	Antibodies
☐	☒	Eukaryotic cell lines
☒	☐	Palaeontology and archaeology
☒	☐	Animals and other organisms
☒	☐	Human research participants
☒	☐	Clinical data
☒	☐	Dual use research of concern

Methods

n/a	Involved in the study	
☐	☒	ChIP-seq
☒	☐	Flow cytometry
☒	☐	MRI-based neuroimaging

Antibodies

Antibodies used

rabbit anti-IgG (1:100 Abcam ab172730)
rabbit anti-MSL2 (1:100 gift from Mitzi Kuroda, Harvard Medical School)
rabbit anti-H4K16ac (1:100 Abcam ab109463)
rabbit anti-H3K27me3 (1:100 Cell Signaling Technology CST9733)
rabbit anti-H3K36me3 (1:100 Thermo MA5-24687)
rabbit anti-H3K4me3 (1:100 Thermo 711958)
rabbit anti-m6A (1:100 Megabase AP60500)
rabbit anti-METTL3 (1:100 Proteintech 15073-1-AP)
mouse anti-unphosphorylated RNA polymerase II (1:100 Abcam ab817)
rabbit anti-GAF (1:100 gift from Giovanni Cavalli, CNRS Montpellier France)
Guinea Pig anti-Rabbit (1:100 Antibodies Online ABIN101961)

	Rabbit anti-Mouse (1:100 Abcam ab46450)<br>Streptavidin Conjugated Secondary Antibody was generated using the Rabbit anti-Mouse antibody (1:100 Abcam ab46450) using the Streptavidin Conjugation Kit (Abcam ab102921) as per manufacturer's instructions
Validation	All antibodies are commercially available or were from previously published reports, and have been verified by Western blotting or by peptide ELISA described on the manufacturer's specification sheets. All antibodies used in this study are confirmed to recognize the human protein as stated on the manufactures website.

Eukaryotic cell lines

Policy information about cell lines

Cell line source(s)	Drosophila Schneider S2 cells were obtained from Invitrogen (10831-014)
Authentication	No authentication performed as part of this study.
Mycoplasma contamination	All cell lines were confirmed as mycoplasma negative on a tri-monthly basis.
Commonly misidentified lines<br>(See ICLAC register)	No commonly misidentified lines were used in this study.

ChIP-seq

Data deposition

☒ Confirm that both raw and final processed data have been deposited in a public database such as GEO.

☒ Confirm that you have deposited or provided access to graph files (e.g. BED files) for the called peaks.

Data access links<br>*May remain private before publication.*	https://www.ncbi.nlm.nih.gov/geo/query/acc.cgi?acc=GSE195654<br><br>MSL2 peak bed file is deposited in https://github.com/nadiyakhyzha/RTTag_Analysis
Files in database submission	S2_RT&Tag_IgG_n1<br>S2_RT&Tag_MSL2_n1<br>S2_RT&Tag_H3K27me3_n1<br>S2_RT&Tag_IgG_n2<br>S2_RT&Tag_MSL2_n2<br>S2_RT&Tag_H3K27me3_n2<br>S2_RT&Tag_IgG_n3<br>S2_RT&Tag_MSL2_n3<br>S2_RT&Tag_H3K27me3_n3<br>S2_RT&Tag_IgG_n4<br>S2_RT&Tag_MSL2_n4<br>S2_RT&Tag_H3K27me3_n4<br>S2_RT&Tag_IgG_n5<br>S2_RT&Tag_H3K27me3_n5<br>S2_RT&Tag_m6A_IgGcontrol_n1<br>S2_RT&Tag_m6A_n1<br>S2_RT&Tag_m6A_n2<br>S2_RT&Tag_m6A_n3<br>S2_RT&Tag_controlRNAi_IgG_n1<br>S2_RT&Tag_controlRNAi_m6A_n1<br>S2_RT&Tag_METTL3RNAi_IgG_n1<br>S2_RT&Tag_METTL3RNAi_m6A_n1<br>S2_RT&Tag_controlRNAi_IgG_n2<br>S2_RT&Tag_controlRNAi_m6A_n2<br>S2_RT&Tag_METTL3RNAi_IgG_n2<br>S2_RT&Tag_METTL3RNAi_m6A_n2<br>S2_RT&Tag_noHS_IgG_n1<br>S2_RT&Tag_noHS_m6A_n1<br>S2_RT&Tag_HS_IgG_n1<br>S2_RT&Tag_HS_m6A_n1<br>S2_RT&Tag_noHS_IgG_n2<br>S2_RT&Tag_noHS_m6A_n2<br>S2_RT&Tag_HS_IgG_n2<br>S2_RT&Tag_HS_m6A_n2<br>S2_WC_RNAseq_n1<br>S2_WC_RNAseq_n2<br>S2_CUT&Tag_MSL2_n1<br>S2_CUT&Tag_MSL2_n2<br>S2_CUT&Tag_H4K16ac<br>S2_CUT&Tag_H3K27me3_n1<br>S2_CUT&Tag_H3K27me3_n2

S2_CUT&Tag_H3K4me3
S2_CUT&Tag_H3K36me3
S2_CUT&Tag_IgG_n3
S2_CUT&Tag_IgG_n1
S2_CUT&Tag_IgG_n2
S2_CUT&Tag_METTL3_n3
S2_CUT&Tag_METTL3_n1
S2_CUT&Tag_METTL3_n2
S2_CUT&Tag_RNAPolII_n1
S2_CUT&Tag_RNAPolII_n2
S2_CUT&Tag_IgG_HS_n1
S2_CUT&Tag_IgG_HS_n2
S2_CUT&Tag_METTL3_HS_n1
S2_CUT&Tag_METTL3_HS_n2
S2_CUT&Tag_RNAPolII_HS_n1
S2_CUT&Tag_RNAPolII_HS_n2
S2_CUT&Tag_GAF
S2_RT&Tag_IgG_coTagRT_n1
S2_RT&Tag_H3K27me3_coTagRT_n1
S2_RT&Tag_MSL2_coTagRT_n1
S2_RT&Tag_m6A_IgGcontrol_coTagRT_n1
S2_RT&Tag_m6A_coTagRT_n1
S2_RT&Tag_IgG_coTagRT_n2
S2_RT&Tag_H3K27me3_coTagRT_n2
S2_RT&Tag_MSL2_coTagRT_n2
S2_RT&Tag_m6A_IgGcontrol_coTagRT_n2
S2_RT&Tag_m6A_coTagRT_n2
S2_RT&Tag_IgG_preTagRT_n1
S2_RT&Tag_H3K27me3_preTagRT_n1
S2_RT&Tag_MSL2_preTagRT_n1
S2_RT&Tag_m6A_IgGcontrol_preTagRT_n1
S2_RT&Tag_m6A_preTagRT_n1
S2_RT&Tag_IgG_preTagRT_n2
S2_RT&Tag_H3K27me3_preTagRT_n2
S2_RT&Tag_MSL2_preTagRT_n2
S2_RT&Tag_m6A_IgGcontrol_preTagRT_n2
S2_RT&Tag_m6A_preTagRT_n2
S2_RT&Tag_IgG_100knuclei_n1
S2_RT&Tag_H3K27me3_100knuclei_n1
S2_RT&Tag_m6A_100knuclei_n1
S2_RT&Tag_IgG_25knuclei_n1
S2_RT&Tag_H3K27me3_25knuclei_n1
S2_RT&Tag_m6A_25knuclei_n1
S2_RT&Tag_IgG_5knuclei_n1
S2_RT&Tag_H3K27me3_5knuclei_n1
S2_RT&Tag_m6A_5knuclei_n1
S2_RT&Tag_IgG_100knuclei_n2
S2_RT&Tag_H3K27me3_100knuclei_n2
S2_RT&Tag_m6A_100knuclei_n2
S2_RT&Tag_IgG_25knuclei_n2
S2_RT&Tag_H3K27me3_25knuclei_n2
S2_RT&Tag_m6A_25knuclei_n2
S2_RT&Tag_IgG_5knuclei_n2
S2_RT&Tag_H3K27me3_5knuclei_n2
S2_RT&Tag_m6A_5knuclei_n2

Genome browser session
(e.g. UCSC)

http://genome.ucsc.edu/s/nkhyzha/Figure_2_Supplementary_3
http://genome.ucsc.edu/s/nkhyzha/Figure_3
http://genome.ucsc.edu/s/nkhyzha/Figure_4
http://genome.ucsc.edu/s/nkhyzha/Figure_5
http://genome.ucsc.edu/s/nkhyzha/Supplementary_Figure_6
http://genome.ucsc.edu/s/nkhyzha/Supplementary_Figure_8

Methodology

Replicates

At least 2 replicates were performed.

Sequencing depth

RT&Tag experiments were single-end sequenced and CUT&Tag experiments were paired-end sequenced. Sequencing depths were 3-10 million reads.

Antibodies

rabbit anti-IgG (1:100 Abcam ab172730)
rabbit anti-MSL2 (1:100 gift from Mitzi Kuroda, Harvard Medical School)
rabbit anti-H4K16ac (1:100 Abcam ab109463)
rabbit anti-H3K27me3 (1:100 Cell Signaling Technology CST9733)

rabbit anti-H3K36me3 (1:100 Thermo MA5-24687)
rabbit anti-H3K4me3 (1:100 Thermo 711958)
rabbit anti-m6A (1:100 Megabase AP60500)
rabbit anti-METTL3 (1:100 Proteintech 15073-1-AP)
mouse anti-unphosphorylated RNA polymerase II (1:100 Abcam ab817)
rabbit anti-GAF (1:100 gift from Giovanni Cavalli, CNRS Montpellier France)
Guinea Pig anti-Rabbit (1:100 Antibodies Online ABIN101961)
Rabbit anti-Mouse (1:100 Abcam ab46450)
Streptavidin Conjugated Secondary Antibody was generated using the Rabbit anti-Mouse antibody (1:100 Abcam ab46450) using the Streptavidin Conjugation Kit (Abcam ab102921) as per manufacturer's instructions

Peak calling parameters

Peaks were called using SEACR by normalizing to IgG control.

Data quality

Quality of raw fastq files were assessed using FastQC (0.11.7). Alignment rates were assessed used HISAT2 (2.1.0). Read duplication rates were assessed using Samtools (1.11). Distribution of reads across gene bodies was assessed using RSeQC (2.6.4) and distribution of genes aligning to exons or introns was assessed using Qualimap (2.2.2).

Software

FastQC (0.11.7); Trim_Galore (0.6.5); HISAT2 (2.1.0); Bowtie2 (2.4.2); Subread featureCounts (2.0.0); RSeQC (2.6.4); MEME (5.3.3); Samtools (1.11); SEACR (1.3); Bedtools (2.30.0); Deeptools (3.5.1); Qualimap (2.2.2); R version 4.1.1, R libraries used: rtracklayer (1.52.1), DESeq2 (1.32.0), ggplot2 (3.3.6), ggrepel (0.9.1), GenomicRanges (1.44.0), karyoploteR (1.18.0), gplots (3.1.3), VennDiagram (1.7.3), viridis (0.6.2), tidyverse (1.3.1), hrbrthemes (0.8.0), clusterProfiler (4.0.5), org.Dm.eg.db (3.13.0); Custom Code is uploaded to GitHub which can be accessed with the following link: https://github.com/nadiyakhyzha/RTTag_Analysis.
