# Supplementary material for: Profiling RNA at chromatin targets in situ by antibody-targeted tagmentation
Source: Nat Methods. 2022 Oct 3;19(11):1383–92. doi: 10.1038/s41592-022-01618-9 (PMC9636022; doi:10.1038/s41592-022-01618-9)
Supplement: Supplementary file 2 — Peer Review File [file 41592_2022_1618_MOESM2_ESM.pdf]

nature portfolio

Peer Review Information

Journal: Nature Methods
Manuscript Title: Profiling RNA at chromatin targets in situ by antibody-targeted tagmentation
Corresponding author names: Steven Henikoff, Kami Ahmad

Reviewer Comments & Decisions:

Decision Letter, initial version:

Subject: Decision on Nature Methods submission NMETH-A48191
Message:

14th Mar 2022

Dear Steve,

Your Article, "Profiling RNA at chromatin targets in situ by antibody-targeted tagmentation", has now been seen by 3 reviewers. As you will see from their comments below, although the reviewers find your work of considerable potential interest, they have raised a number of concerns. We are interested in the possibility of publishing your paper in Nature Methods, but would like to consider your response to these concerns before we reach a final decision on publication.

We therefore invite you to revise your manuscript to address these concerns. The revision should include, but not limited to, benchmarking comparison with existing tools as well as the demonstration of RT&Tag's unique capability in contrast to existing methods.

We are committed to providing a fair and constructive peer-review process. Do not hesitate to contact us if there are specific requests from the reviewers that you believe are technically impossible or unlikely to yield a meaningful outcome.

When revising your paper:

* include a point-by-point response to the reviewers and to any editorial suggestions

* please underline/highlight any additions to the text or areas with other significant changes to facilitate review of the revised manuscript

* address the points listed described below to conform to our open science requirements

* ensure it complies with our general format requirements as set out in our guide to authors at www.nature.com/naturemethods

Open Access This file is licensed under a Creative Commons Attribution 4.0 International License, which permits use, sharing, adaptation, distribution and reproduction in any medium or format, as long as you give appropriate credit to the original author(s) and the source, provide a link to the Creative Commons license, and indicate if changes were made. In the cases where the authors are anonymous, such as is the case for the reports of anonymous peer reviewers, author attribution should be to 'Anonymous Referee' followed by a clear attribution to the source work. The images or other third party material in this file are included in the article's Creative Commons license, unless indicated otherwise in a credit line to the material. If material is not included in the article's Creative Commons license and your intended use is not permitted by statutory regulation or exceeds the permitted use, you will need to obtain permission directly from the copyright holder. To view a copy of this license, visit http://creativecommons.org/licenses/by/4.0/.

* resubmit all the necessary files electronically by using the link below to access your home page

[REDACTED]

Note: This URL links to your confidential home page and associated information about manuscripts you may have submitted, or that you are reviewing for us. If you wish to forward this email to co-authors, please delete the link to your homepage.

We hope to receive your revised paper within 8 weeks. If you cannot send it within this time, please let us know. In this event, we will still be happy to reconsider your paper at a later date so long as nothing similar has been accepted for publication at Nature Methods or published elsewhere.

OPEN SCIENCE REQUIREMENTS

REPORTING SUMMARY AND EDITORIAL POLICY CHECKLISTS
When revising your manuscript, please update your reporting summary and editorial policy checklists.

Reporting summary: https://www.nature.com/documents/nr-reporting-summary.zip
Editorial policy checklist: https://www.nature.com/documents/nr-editorial-policy-checklist.zip

If your paper includes custom software, we also ask you to complete a supplemental reporting summary.

Software supplement: https://www.nature.com/documents/nr-software-policy.pdf

Please submit these with your revised manuscript. They will be available to reviewers to aid in their evaluation if the paper is re-reviewed. If you have any questions about the checklist, please see http://www.nature.com/authors/policies/availability.html or contact me.

Please note that these forms are dynamic 'smart pdfs' and must therefore be downloaded and completed in Adobe Reader. We will then flatten them for ease of use by the reviewers. If you would like to reference the guidance text as you complete the template, please access these flattened versions at http://www.nature.com/authors/policies/availability.html.

Open Access This file is licensed under a Creative Commons Attribution 4.0 International License, which permits use, sharing, adaptation, distribution and reproduction in any medium or format, as long as you give appropriate credit to the original author(s) and the source, provide a link to the Creative Commons license, and indicate if changes were made. In the cases where the authors are anonymous, such as is the case for the reports of anonymous peer reviewers, author attribution should be to 'Anonymous Referee' followed by a clear attribution to the source work. The images or other third party material in this file are included in the article's Creative Commons license, unless indicated otherwise in a credit line to the material. If material is not included in the article's Creative Commons license and your intended use is not permitted by statutory regulation or exceeds the permitted use, you will need to obtain permission directly from the copyright holder. To view a copy of this license, visit http://creativecommons.org/licenses/by/4.0/.

IMAGE INTEGRITY
When submitting the revised version of your manuscript, please pay close attention to our Digital Image Integrity Guidelines and to the following points below:

-- that unprocessed scans are clearly labelled and match the gels and western blots presented in figures.
-- that control panels for gels and western blots are appropriately described as loading on sample processing controls
-- all images in the paper are checked for duplication of panels and for splicing of gel lanes.

Finally, please ensure that you retain unprocessed data and metadata files after publication, ideally archiving data in perpetuity, as these may be requested during the peer review and production process or after publication if any issues arise.

We strongly encourage you to deposit all new data associated with the paper in a persistent repository where they can be freely and enduringly accessed. We recommend submitting the data to discipline-specific and community-recognized repositories; a list of repositories is provided here: http://www.nature.com/sdata/policies/repositories

All novel DNA and RNA sequencing data, protein sequences, genetic polymorphisms, linked genotype and phenotype data, gene expression data, macromolecular structures, and proteomics data must be deposited in a publicly accessible database, and accession codes and associated hyperlinks must be provided in the "Data Availability" section.

Refer to our data policies here: https://www.nature.com/nature-research/editorial-policies/reporting-standards#availability-of-data

To further increase transparency, we encourage you to provide, in tabular form, the data underlying the graphical representations used in your figures. This is in addition to our data-deposition policy for specific types of experiments and large datasets. For readers, the source data will be made accessible directly from the figure legend. Spreadsheets can be submitted in .xls, .xlsx or .csv formats. Only one (1) file per figure is permitted: thus if there is a multi-paneled figure the source data for each panel should be clearly labeled in the csv/Excel file; alternately the data for a figure can be included in multiple, clearly labeled sheets in an Excel file. File sizes of up to 30 MB are permitted. When submitting source data files with your manuscript please select the Source Data file type and use the Title field in the File Description tab to indicate which figure the source data pertains to.

Open Access This file is licensed under a Creative Commons Attribution 4.0 International License, which permits use, sharing, adaptation, distribution and reproduction in any medium or format, as long as you give appropriate credit to the original author(s) and the source, provide a link to the Creative Commons license, and indicate if changes were made. In the cases where the authors are anonymous, such as is the case for the reports of anonymous peer reviewers, author attribution should be to 'Anonymous Referee' followed by a clear attribution to the source work. The images or other third party material in this file are included in the article's Creative Commons license, unless indicated otherwise in a credit line to the material. If material is not included in the article's Creative Commons license and your intended use is not permitted by statutory regulation or exceeds the permitted use, you will need to obtain permission directly from the copyright holder. To view a copy of this license, visit http://creativecommons.org/licenses/by/4.0/.

Please include a “Data availability” subsection in the Online Methods. This section should inform readers about the availability of the data used to support the conclusions of your study, including accession codes to public repositories, references to source data that may be published alongside the paper, unique identifiers such as URLs to data repository entries, or data set DOIs, and any other statement about data availability. At a minimum, you should include the following statement: “The data that support the findings of this study are available from the corresponding author upon request”, describing which data is available upon request and mentioning any restrictions on availability. If DOIs are provided, please include these in the Reference list (authors, title, publisher (repository name), identifier, year). For more guidance on how to write this section please see: http://www.nature.com/authors/policies/data/data-availability-statements-data-citations.pdf

CODE AVAILABILITY
Please include a “Code Availability” subsection in the Online Methods which details how your custom code is made available. Only in rare cases (where code is not central to the main conclusions of the paper) is the statement “available upon request” allowed (and reasons should be specified).

We request that you deposit code in a DOI-minting repository such as Zenodo, Gigantum or Code Ocean and cite the DOI in the Reference list. We also request that you use code versioning and provide a license.

For more information on our code sharing policy and requirements, please see: https://www.nature.com/nature-research/editorial-policies/reporting-standards#availability-of-computer-code

SUPPLEMENTARY PROTOCOL
To help facilitate reproducibility and uptake of your method, we ask you to prepare a step-by-step Supplementary Protocol for the method described in this paper. We encourage authors to share their step-by-step experimental protocols on a protocol sharing platform of their choice and report the protocol DOI in the reference list. Nature Research's Protocol Exchange is a free-to-use and open resource for protocols; protocols deposited in Protocol Exchange are citable and can be linked from the published article. More details can found at www.nature.com/protocolexchange/about.

Open Access This file is licensed under a Creative Commons Attribution 4.0 International License, which permits use, sharing, adaptation, distribution and reproduction in any medium or format, as long as you give appropriate credit to the original author(s) and the source, provide a link to the Creative Commons license, and indicate if changes were made. In the cases where the authors are anonymous, such as is the case for the reports of anonymous peer reviewers, author attribution should be to 'Anonymous Referee' followed by a clear attribution to the source work. The images or other third party material in this file are included in the article’s Creative Commons license, unless indicated otherwise in a credit line to the material. If material is not included in the article’s Creative Commons license and your intended use is not permitted by statutory regulation or exceeds the permitted use, you will need to obtain permission directly from the copyright holder. To view a copy of this license, visit http://creativecommons.org/licenses/by/4.0/.

nature portfolio

ORCID
Nature Methods is committed to improving transparency in authorship. As part of our efforts in this direction, we are now requesting that all authors identified as 'corresponding author' on published papers create and link their Open Researcher and Contributor Identifier (ORCID) with their account on the Manuscript Tracking System (MTS), prior to acceptance. This applies to primary research papers only. ORCID helps the scientific community achieve unambiguous attribution of all scholarly contributions. You can create and link your ORCID from the home page of the MTS by clicking on 'Modify my Springer Nature account'. For more information please visit please visit www.springernature.com/orcid.

Please do not hesitate to contact me if you have any questions or would like to discuss these revisions further. We look forward to seeing the revised manuscript and thank you for the opportunity to consider your work.

Best regards,
Lei

Lei Tang, Ph.D.
Senior Editor
Nature Methods

Reviewers' Comments:

Reviewer #1:
Remarks to the Author:
In this manuscript, Khyzha et al. reports a new method RT&Tag for profiling chromatin- and RBPs-associated RNAs as well as m6A modified transcripts via a strategy of in situ antibody tethering and tagmentation based on Protein A-Tn5. The authors showcase their approach by first profiling MSL2 coated on X chr in Drosophila cells, and measuring transcripts within chromatin domains (H3K27me3) and with m6A modification. Integrated analyses of separately obtained RT&Tag and CUT&Tag data confirm the relationship between transcripts identified by RT&Tag of different classes (enriched/non-enriched) and histone modifications (or protein binding). Furthermore, the authors calculate Pause

Open Access This file is licensed under a Creative Commons Attribution 4.0 International License, which permits use, sharing, adaptation, distribution and reproduction in any medium or format, as long as you give appropriate credit to the original author(s) and the source, provide a link to the Creative Commons license, and indicate if changes were made. In the cases where the authors are anonymous, such as is the case for the reports of anonymous peer reviewers, author attribution should be to 'Anonymous Referee' followed by a clear attribution to the source work. The images or other third party material in this file are included in the article's Creative Commons license, unless indicated otherwise in a credit line to the material. If material is not included in the article's Creative Commons license and your intended use is not permitted by statutory regulation or exceeds the permitted use, you will need to obtain permission directly from the copyright holder. To view a copy of this license, visit http://creativecommons.org/licenses/by/4.0/.

Index to gain insight into the mechanism of transcript m6A. Overall, this method is a potentially interesting addition to the Protein A-Tn5 application.

However, as the method currently stands, RT&Tag requires at least 100,000 cells as input, which largely dampens the enthusiasms for the technical advance and potential applicability. There are many published relevant technologies, such as CLIP and its variants (iCLIP, eCLIP, and irCLIP), with high sensitivity in profiling RBPs bound RNAs. A recent method, LACE-seq (Su et al., Nat Cell Biol, 2021) is able to measure RBPs-RNA interaction in single cells. Therefore, pairwise comparisons with these technologies as golden standard are essential, but lacking here. Importantly, simultaneously profiling transcripts and chromatin binding regions of histone marks is a plus or major step forward to understanding the regulatory kinship between these two modalities, whilst RT&Tag is designed to eliminate the information of the latter. Further, the application of RT&Tag in measuring H3K27me3 associated transcripts did not seem to be an appropriate example since H3K27me3-enriched regions are in general silenced. Hence, this inevitably yielded a very limited insight into gene regulation. Together, new biological findings were rarely obtained from these three applications, requiring a large amount of input materials with very noisy data quality. Thus, a major advance is lacking in the current format.

Specific comments:
1. Data quality. Volcano plots in Fig. 2D, Fig. 3B, Fig. 4B show marginal differences between IgG and antibody enriched groups, suggesting highly noisy data. Track view in Fig. 3C shows similar patterns between IgG and RT&Tag (antibody group), again indicating low signal to noise. The same is also applied to Fig. 4C.
2. To demonstrate the reliability and robustness of RT&Tag, particularly for the application in small samples, RT&Tag using varying cell numbers (100K, 10k, 1k, 100, or even single cells) should be performed and analyzed in parallel with other similar techniques, such LACE-seq on RBPs associated transcripts.
3. The authors performed several analyses in Fig. 1 and Supplementary Fig. 1 to assess the data quality of RT&Tag. However, global evaluation of RT&Tag is lacking. The information including mapping rate, total UMIs, detected genes can be presented. Also, it is not clear whether RT&Tag outperforms PIRCh-seq and ChRIP-seq, and MeRIP-seq. It would be helpful to count the genes overlapped with those from these conventional
4. Since protein-bound RNAs and transcripts within the vicinity of the protein in space are both detected by RT&Tag, is there any way to distinguish them if only interested in protein-bound RNAs?
5. In Fig. 3h, it is unclear how the rows within different groups in the heatmap are sorted, which needs to be clarified in the figure legend.

Open Access This file is licensed under a Creative Commons Attribution 4.0 International License, which permits use, sharing, adaptation, distribution and reproduction in any medium or format, as long as you give appropriate credit to the original author(s) and the source, provide a link to the Creative Commons license, and indicate if changes were made. In the cases where the authors are anonymous, such as is the case for the reports of anonymous peer reviewers, author attribution should be to 'Anonymous Referee' followed by a clear attribution to the source work. The images or other third party material in this file are included in the article's Creative Commons license, unless indicated otherwise in a credit line to the material. If material is not included in the article's Creative Commons license and your intended use is not permitted by statutory regulation or exceeds the permitted use, you will need to obtain permission directly from the copyright holder. To view a copy of this license, visit http://creativecommons.org/licenses/by/4.0/.

6. It has been reported that bivalent domains (H3K4me3 and H3K27me3) tend to coincide with TF genes expressed at low levels in mouse ES cells (PMID: 16630819). The authors find that low production of new transcripts is from silenced regions, which is not surprising to me. What biological processes are the 208 H3K27me3 RT&Tag-enriched transcripts associated with?
7. The authors performed RNAPolII CUT&Tag using antibody of unphosphorylated RNA polymerase II that functions at early pre-initiating stage during transcription (PMID: 17079683), and the signals at gene body are hardly detected using the antibody in theory. Thus, the analysis of pause index (TSS density/gene body density) is not convincing to me.
8. The important link between RNAPolII pausing and the m6A RNA modification has been reported (PMID: 34297910), which cuts down the novelty of this manuscript.
9. To really establish the regulatory relationship between m6A and RNAPolII, the authors need to simultaneously profile both molecular information in the same single cells. This may represent a major advance to the broad field of epigenetics.

Reviewer #2:
Remarks to the Author:
"Profiling RNA at chromatin targets in situ by antibody-targeted tagmentation."

Khyzha et al. developed a high throughput technology to capture and amplify RNA molecules within their native chromatin or regulatory protein complexes. The authors successfully quantified RNA transcripts localized at Polycomb-targeted chromatin modification and RNA enriched with methylation modification (m6A). The key feature of this method is the elimination of crosslinking, use of Tn5 transposition at targeted RNA/cDNA heteroduplexes, and targeted amplification of localized RNA molecules from a small number of intact nuclei. This methodology allows gaining insight into RNA-guided chromatin regulation and epitranscriptomic studies. The scalability of RT&Tag in capturing RNA from its native chromatin site is an improvement compared to other RNA pulldown protocols. In addition to providing a new method, the authors generated some biological insight by finding a correlation between m6A methylation and RNAPII pausing on nascent transcripts. However, a number of shortcomings needs to be addressed.

Specific Comments:

Open Access This file is licensed under a Creative Commons Attribution 4.0 International License, which permits use, sharing, adaptation, distribution and reproduction in any medium or format, as long as you give appropriate credit to the original author(s) and the source, provide a link to the Creative Commons license, and indicate if changes were made. In the cases where the authors are anonymous, such as is the case for the reports of anonymous peer reviewers, author attribution should be to 'Anonymous Referee' followed by a clear attribution to the source work. The images or other third party material in this file are included in the article's Creative Commons license, unless indicated otherwise in a credit line to the material. If material is not included in the article's Creative Commons license and your intended use is not permitted by statutory regulation or exceeds the permitted use, you will need to obtain permission directly from the copyright holder. To view a copy of this license, visit http://creativecommons.org/licenses/by/4.0/.

• The authors should compare their RT&Tag method to others, regarding cell number, the cost of reagents, and the sequencing depth, in order for readers to appreciate the extent of the advance.

• What is the effect of potential variation in reverse transcription for targeted RNA amplification?

• What is the spatial resolution of this method? For example, it would be interesting to quantify and compare the spread of captured RNA at regulatory regions with extended modification (e. g., H3K27me3) to compact and punctuated states.

• While the correlation between m6A methylation and RNAPII pausing on nascent transcripts is interesting, the authors have not provided evidence of function or causality. Does m6A cause pausing or does pausing cause m6A? Or is the correlation a red herring?

Comments on figures:

Figure 2D: It might help to generate a separate figure for each experiment: the H3K27me, me6A, and MSL2 associate transcripts and compare the classification of RNA molecules between the methodologies.

Supplementary Figure 1: The authors provide RNA read counts obtained from the simultaneous and sequential reverse transcription experiments. The experiment is informative, but the authors did not discuss the results. Additionally, they could also compare the length of RNA molecules between different conditions.

Supplementary Figure 1A & B: The 'performance comparison' in control conditions (Co-Tag-RT and Biotinylated oligo(dT)) for H3K4me27 RT&Tag is vastly different (~200 in A, compared to ~40 in B). Did they execute the control experiments similarly?

Figure 2C: Please classify captured RNA molecules from all three experiments: the H3K27me, me6A, and MSL2 associated transcripts.

Figure 3F: Please include H3K4me1 associated RT&Tag signal and overlapping regions with H3K27me, which recognize bivalent regulatory regions at distal promoter and enhancer sites.

Open Access This file is licensed under a Creative Commons Attribution 4.0 International License, which permits use, sharing, adaptation, distribution and reproduction in any medium or format, as long as you give appropriate credit to the original author(s) and the source, provide a link to the Creative Commons license, and indicate if changes were made. In the cases where the authors are anonymous, such as is the case for the reports of anonymous peer reviewers, author attribution should be to 'Anonymous Referee' followed by a clear attribution to the source work. The images or other third party material in this file are included in the article's Creative Commons license, unless indicated otherwise in a credit line to the material. If material is not included in the article's Creative Commons license and your intended use is not permitted by statutory regulation or exceeds the permitted use, you will need to obtain permission directly from the copyright holder. To view a copy of this license, visit http://creativecommons.org/licenses/by/4.0/.

Supplementary Figure 3: Since the MSL4 CUT&Tag signal seems highly variable (scale is 0 to 150 compared to 0 to 1000) and also H4K16ac seem comparably similar in both browser traces, I suggest providing quartile comparisons of RNA reads at MSL4 'enriched' and 'nonenriched' sites and removing H4K16Ac track, entirely.

Figure 5H: Since genes with lower steady-state expression tend to exhibit 'longer' RNAPII pausing, it makes sense to calculate the pausing index at m6A genes for two separate groups of highly expressed and lowly expressed genes Please see:

Reviewer #3:
Remarks to the Author:
In this manuscript, Henikoff and colleagues present a new method called RT&TAG. This approach extends their widely used CUT&TAG technology (which maps DNA sites in proximity to specific proteins) to enable mapping of RNAs that are in proximity to a specific protein of interest in the nucleus. They show that this approach is able to recover the well-characterized dosage compensation ncRNA (roX2) when exploring the MSL complex along with other X-linked genes. They similarly show two other proof-of-concept demonstrations related to m6A methylation and polycomb associated RNAs.

Overall, the authors provide a compelling technical demonstration that the RT&TAG approach is accurate and works well in defining RNAs that are in proximity to their targeted proteins. I think this is likely to be a valuable method for the RNA and chromatin communities and am strongly in favor of its publication in Nature Methods. However, I do have concerns related to how several key points are framed and the interpretation of RT&TAG results that I believe are critical for the authors to address in the writing of the manuscript prior to publication.

I think it is important for the authors to more clearly explain what exactly they are measuring and, equally importantly, what they are NOT measuring. The gold-standard for measuring RNA-protein interactions is UV-based CLIP. This approach uses highly stringent crosslinking (UV which only forms covalent crosslinks between direct RNA and protein contacts that occur within "zero distance) along with highly stringent purification methods (high-salt washes and separation and purification through a denaturing SDS-PAGE gel) to map direct RNA-protein contacts. I think it is important for the authors to clearly point out that RT&TAG is NOT limited to detection of direct RNA-protein interactions. As currently written (especially when comparing the ~150 ENCODE proteins mapped by CLIP in the

Open Access This file is licensed under a Creative Commons Attribution 4.0 International License, which permits use, sharing, adaptation, distribution and reproduction in any medium or format, as long as you give appropriate credit to the original author(s) and the source, provide a link to the Creative Commons license, and indicate if changes were made. In the cases where the authors are anonymous, such as is the case for the reports of anonymous peer reviewers, author attribution should be to 'Anonymous Referee' followed by a clear attribution to the source work. The images or other third party material in this file are included in the article's Creative Commons license, unless indicated otherwise in a credit line to the material. If material is not included in the article's Creative Commons license and your intended use is not permitted by statutory regulation or exceeds the permitted use, you will need to obtain permission directly from the copyright holder. To view a copy of this license, visit http://creativecommons.org/licenses/by/4.0/.

discussion) one could read this paper as claiming that it is a replacement for these approaches which it is clearly not.

Similarly, the authors should clearly note that what they are measuring are RNAs that are in proximity to a protein of interest not necessarily those that directly or even indirectly bind or associate with the protein. This is a distinction that leads to a major source of confusion in the RNA-protein field and therefore is critical to explicitly discuss. The distinction is that nascent RNAs are in proximity to MSL or polycomb but do not necessarily bind to them either directly or indirectly. To make this more concrete, imagine a protein that is associated with transcribed DNA (say H3K36me3), you would presumably detect RNAs with it using RT&TAG because of its proximity to nascent transcripts but this does NOT imply that H3K36me3 binds to RNA. This is by no means limited to RT&TAG (RIP-Seq also has this issue) but this distinction is important and often confusing.

Finally, the authors argue that this approach specifically maps chromatin associated RNPs. It is possible that I missed this key technical point, but from my understanding of the method there is nothing that limits the associations detected to those that occur on chromatin. If I understand correctly, any RNA that is in proximity to their protein in the nucleus would be tagged whether on chromatin or nucleoplasm. If my understanding is correct, this is certainly not a problem for the method but the authors should adjust their wording to avoid implying chromatin specific mapping. If I am misunderstanding the method, the authors should clarify their description in section 1 to more explicitly explain why this approach is limited to chromatin and not nucleoplasmic associations.

Author Rebuttal to Initial comments

Reviewer #1:
Remarks to the Author:
In this manuscript, Khyzha et al. reports a new method RT&Tag for profiling chromatin- and RBPs-associated RNAs as well as m6A modified transcripts via a strategy of in situ antibody tethering and tagmentation based on Protein A-Tn5. The authors showcase their approach by first profiling MSL2 coated on X chr in Drosophila cells, and measuring transcripts within chromatin domains (H3K27me3) and with m6A modification. Integrated analyses of separately obtained RT&Tag and CUT&Tag data confirm the relationship between transcripts identified by RT&Tag of different classes (enriched/non-enriched) and histone modifications (or protein binding). Furthermore, the authors calculate Pause Index to gain insight into the mechanism of transcript m6A. Overall, this method is a potentially interesting addition to the Protein A-Tn5 application.

We thank Reviewer 1 for this positive overall assessment of our method.

Open Access This file is licensed under a Creative Commons Attribution 4.0 International License, which permits use, sharing, adaptation, distribution and reproduction in any medium or format, as long as you give appropriate credit to the original author(s) and the source, provide a link to the Creative Commons license, and indicate if changes were made. In the cases where the authors are anonymous, such as is the case for the reports of anonymous peer reviewers, author attribution should be to 'Anonymous Referee' followed by a clear attribution to the source work. The images or other third party material in this file are included in the article's Creative Commons license, unless indicated otherwise in a credit line to the material. If material is not included in the article's Creative Commons license and your intended use is not permitted by statutory regulation or exceeds the permitted use, you will need to obtain permission directly from the copyright holder. To view a copy of this license, visit http://creativecommons.org/licenses/by/4.0/.

However, as the method currently stands, RT&Tag requires at least 100,000 cells as input, which largely dampens the enthusiasms for the technical advance and potential applicability.

We performed RT&Tag with 100,000 cells for convenience, but to address this reviewer's concerns we have tested varying cell numbers for RT&Tag. We find that we can reproduce the enrichment for H3K27me3 and m6A using RT&Tag with fewer cells (new Supplementary Figures 6 and 8). This could be invaluable for applications where cell number is limiting. However, the number of significant hits decreases with fewer cells (see response Figure A) so additional replicates may be needed when working with very low cell numbers.

	H3K27me3	m6A
100k nuclei	244	86
25k nuclei	55	13
5k nuclei	1	0

Figure A. Number of differentially enriched transcripts identified using H3K27me3 (fold change >2, FDR <0.05, n=2) and m6A RT&Tag (fold change >1.5, FDR <0.05, n=2) using varying cell numbers.

There are many published relevant technologies, such as CLIP and its variants (iCLIP, eCLIP, and irCLIP), with high sensitivity in profiling RBPs bound RNAs. A recent method, LACE-seq (Su et al., Nat Cell Biol, 2021) is able to measure RBPs-RNA interaction in single cells.

Published work describing irCLIP used 100,000 or 20,000 cells[1], a similar range to what we use for RT&Tag. LACE-seq uses linear amplification during library preparation to increase the sensitivity of detecting RBP-RNA interactions[2]. However, LACE-seq (and CLIP) are used specifically to profile RNA-protein interactions, which makes them fundamentally different from RT&Tag, which was designed to profile chromatin-associated transcripts (such as lncRNAs) that may be less abundant than RNA-associated factors profiled by CLIP and related methods. RT&Tag uses antibodies to tether Tn5 where it tagments RNA within its vicinity. Thus, RT&Tag is not limited to assaying RNA-protein interactions, and can be applied to a wide range of RNA-chromatin interactions, localizations, and RNA modifications. To highlight this distinction, we have now modified our schematic in Figure 1B. We have also modified the discussion (pg7, lines 270-276) and the introduction (pg2, lines 35-46) to help emphasize this point.

Open Access This file is licensed under a Creative Commons Attribution 4.0 International License, which permits use, sharing, adaptation, distribution and reproduction in any medium or format, as long as you give appropriate credit to the original author(s) and the source, provide a link to the Creative Commons license, and indicate if changes were made. In the cases where the authors are anonymous, such as is the case for the reports of anonymous peer reviewers, author attribution should be to 'Anonymous Referee' followed by a clear attribution to the source work. The images or other third party material in this file are included in the article's Creative Commons license, unless indicated otherwise in a credit line to the material. If material is not included in the article's Creative Commons license and your intended use is not permitted by statutory regulation or exceeds the permitted use, you will need to obtain permission directly from the copyright holder. To view a copy of this license, visit http://creativecommons.org/licenses/by/4.0/.

Therefore, pairwise comparisons with these technologies as golden standard are essential, but lacking here.

We have now included a table comparing RT&Tag to immunoprecipitation-based techniques with regards to cell input, sequencing requirements, and cost (Table 1). RT&Tag compares favorably to other methods in terms of the amount of sequencing required and ease of use. Additionally, we have compared our MSL2 RT&Tag data to a published MLE RIP-seq dataset. This dataset was appropriate as it focused on targeting the same complex in the same cell line. We have now included this comparison in Supplementary Figure 5 and have included a paragraph (pg 4, lines 147-159) describing the findings of this comparison. We show that not only does RT&Tag require fewer cells and less sequencing, the significant hits from RT&Tag are predominantly from the X chromosome, the expected location of the MSL2 complex binding in male cells (see response Figure B).

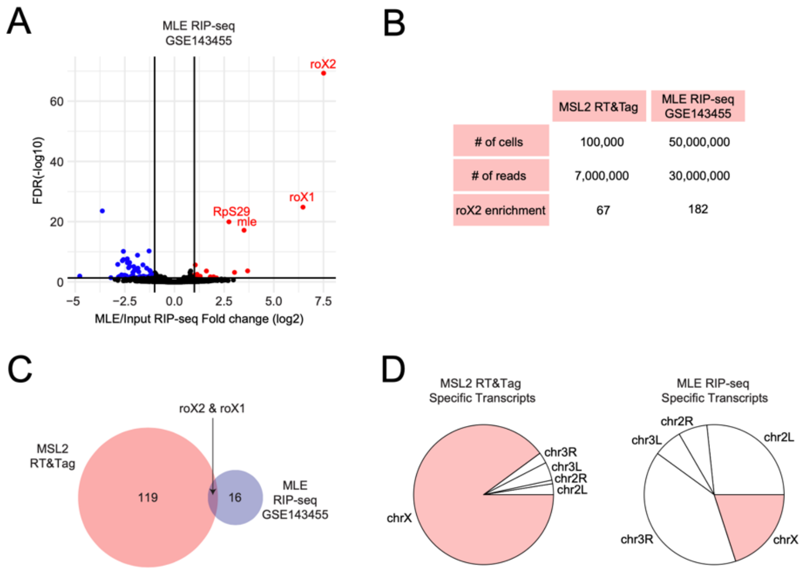

Figure B. A) Volcano plot showing transcripts differentially enriched for MLE RIP-seq over input (fold change >2, FDR <0.05, n=3, GSE143455). Transcripts enriched for MLE are highlighted in red, nonenriched are in black, and depleted are in blue. B) Table comparing MSL2 RT&Tag and MLE RIP-seq in terms of number of cells, number of reads, and roX2 fold change enrichment for MSL2/MLE over control. C) Venn diagram showing the overlap between transcripts enriched for MSL2 RT&Tag and MLE RIP-seq with roX1 and roX2 being enriched in both. D) Pie charts showing the chromosomal distribution of transcripts uniquely enriched for MSL2 RT&Tag (left) and MLE RIP-seq (right).

Open Access This file is licensed under a Creative Commons Attribution 4.0 International License, which permits use, sharing, adaptation, distribution and reproduction in any medium or format, as long as you give appropriate credit to the original author(s) and the source, provide a link to the Creative Commons license, and indicate if changes were made. In the cases where the authors are anonymous, such as is the case for the reports of anonymous peer reviewers, author attribution should be to 'Anonymous Referee' followed by a clear attribution to the source work. The images or other third party material in this file are included in the article's Creative Commons license, unless indicated otherwise in a credit line to the material. If material is not included in the article's Creative Commons license and your intended use is not permitted by statutory regulation or exceeds the permitted use, you will need to obtain permission directly from the copyright holder. To view a copy of this license, visit http://creativecommons.org/licenses/by/4.0/.

Importantly, simultaneously profiling transcripts and chromatin binding regions of histone marks is a plus or major step forward to understanding the regulatory kinship between these two modalities, whilst RT&Tag is designed to eliminate the information of the latter.

We profile the chromatin distribution of binding sites by CUT&Tag, a popular chromatin profiling method for small samples and single cells that our lab previously introduced[3], and we compare the CUT&Tag binding sites to the RNAs identified by RT&Tag. However, we envision modifications to the RT&Tag experimental framework that could allow for simultaneous CUT&Tag/RT&Tag profiling in the future, which would be especially useful if RT&Tag is adapted for single-cell assays. For example, profiling of chromatin using CUT&Tag paired with RNA-seq has previously been done in single cells using paired-Tag[4]. One could use a similar sequential approach as paired-Tag to tagment chromatin with one set of adaptors. Subsequently, reverse transcription and tagmentation could be performed with Tn5 loaded with a different adaptor. Hence, we do not see this point as a limitation of RT&Tag.

Further, the application of RT&Tag in measuring H3K27me3 associated transcripts did not seem to be an appropriate example since H3K27me3-enriched regions are in general silenced. Hence, this inevitably yielded a very limited insight into gene regulation.

We have chosen to identify H3K27me3 associated transcripts based on the numerous reports in mammalian systems highlighting the role of RNA in regulating H3K27me3 marked domains[5]. Our work has uncovered about 15% of H3K27me3 associated transcripts (208 transcripts) to be transcribed from areas outside of Polycomb domains that could be followed up on with functional studies in the future. The intention of our work was to present how a histone modification can be used with our RT&Tag method, which we think is appropriate for a Methods article.

Together, new biological findings were rarely obtained from these three applications, requiring a large amount of input materials with very noisy data quality. Thus, a major advance is lacking in the current format.

The benchmarking that we now provide in Table 1 and in Supplementary Figure 5 makes the point that RT&Tag uses a smaller amount of input and generates better data quality from less sequencing than other RNA profiling methods, thus we believe this will be of interest to readers of *Nature Methods*.

Specific comments:
1. Data quality. Volcano plots in Fig. 2D, Fig. 3B, Fig. 4B show marginal differences between IgG and antibody enriched groups, suggesting highly noisy data. Track view in Fig. 3C shows similar patterns between IgG and RT&Tag (antibody group), again indicating low signal to noise. The same is also applied to Fig. 4C.

Open Access This file is licensed under a Creative Commons Attribution 4.0 International License, which permits use, sharing, adaptation, distribution and reproduction in any medium or format, as long as you give appropriate credit to the original author(s) and the source, provide a link to the Creative Commons license, and indicate if changes were made. In the cases where the authors are anonymous, such as is the case for the reports of anonymous peer reviewers, author attribution should be to 'Anonymous Referee' followed by a clear attribution to the source work. The images or other third party material in this file are included in the article's Creative Commons license, unless indicated otherwise in a credit line to the material. If material is not included in the article's Creative Commons license and your intended use is not permitted by statutory regulation or exceeds the permitted use, you will need to obtain permission directly from the copyright holder. To view a copy of this license, visit http://creativecommons.org/licenses/by/4.0/.

The volcano plots mark statistically significant transcripts in red. Using RT&Tag, we identify 120, 1342, and 281 transcripts enriched for MSL2, H3K27me3, and m6A, respectively. We now point out that the number of transcripts recovered by RT&Tag is comparable to what has been detected with other techniques in Supplementary Figure 5B and in response Figures C and D. We include in Supplementary Figure 5 an analysis showing that MSL2 RT&Tag detects the roX2-MSL2 interaction to a similar degree as with RIP-seq but using 50-fold fewer cells. Likewise, H3K27me3 RT&Tag detects more noncoding RNAs associated with H3K27me3 in S2 cells relative to what has been detected by H3K27me3 PIRCh-seq in mESC cells (164 vs 70, see response Figure C)[6]. The high statistical significance of known interacting RNAs and the numbers of recovered transcripts demonstrates that RT&Tag has good signal to noise.

2. To demonstrate the reliability and robustness of RT&Tag, particularly for the application in small samples, RT&Tag using varying cell numbers (100K, 10k, 1k, 100, or even single cells) should be performed and analyzed in parallel with other similar techniques, such LACE-seq on RBPs associated transcripts.

We have now performed RT&Tag using varying cell numbers (100k, 25k, and 5k) for H3K27me3 and m6A and included this data in Supplementary Figures 6 and 8. Specifically, we show that we are able to reproduce the enrichment of RT&Tag signal for most transcripts with 25k and for some transcripts with 5k cells. LACE-seq uses linear amplification by in vitro transcription to amplify signal in individual cells, and it is likely that single-cell RT&Tag would require a similar innovation. However, we emphasize that RT&Tag has other advantages: LACE-seq is limited to investigating RNA-binding proteins, while RT&Tag can be used for protein-associated RNAs, chromatin-associated RNAs, and modified RNAs. Alternative methods designed for chromatin-associated RNAs, such as PIRCh-seq, ChRIP-seq and MeRIP-seq, require much larger cell inputs.

3. The authors performed several analyses in Fig. 1 and Supplementary Fig. 1 to assess the data quality of RT&Tag. However, global evaluation of RT&Tag is lacking. The information including mapping rate, total UMIs, detected genes can be presented. Also, it is not clear whether RT&Tag outperforms PIRCh-seq and ChRIP-seq, and MeRIP-seq. It would be helpful to count the genes overlapped with those from these conventional

We have now performed global evaluation of RT&Tag including mapping rate, PCR duplication rate and number of detected genes. This information is now included in Supplementary Table 7.

We also compared our results to those published using PIRCh-seq and MeRIP-seq. The only available PIRCh-seq dataset is from the original PIRCh-seq manuscript which was done in mouse[6] and ours in Drosophila, so a direct comparison is not feasible. Nevertheless, we point out that H3K27me3 RT&Tag

Open Access This file is licensed under a Creative Commons Attribution 4.0 International License, which permits use, sharing, adaptation, distribution and reproduction in any medium or format, as long as you give appropriate credit to the original author(s) and the source, provide a link to the Creative Commons license, and indicate if changes were made. In the cases where the authors are anonymous, such as is the case for the reports of anonymous peer reviewers, author attribution should be to 'Anonymous Referee' followed by a clear attribution to the source work. The images or other third party material in this file are included in the article's Creative Commons license, unless indicated otherwise in a credit line to the material. If material is not included in the article's Creative Commons license and your intended use is not permitted by statutory regulation or exceeds the permitted use, you will need to obtain permission directly from the copyright holder. To view a copy of this license, visit http://creativecommons.org/licenses/by/4.0/.

detects more noncoding RNAs associated with H3K27me3 than does PIRCh-seq (164 vs <70, see response Figure C), with many fewer cells and sequencing reads.

	H3K27me3 RT&Tag	H3K27me3 PIRCh-seq GSE119006
# of cells	100,000	5,000,000
# of reads	7,000,000	50,000,000
ncRNAs enrichment	164	<70

Figure C. Table showing the number of cells and sequencing reads used for H3K27me3 RT&Tag and PIRCh-seq and the number of ncRNAs detected as enriched with each technique (fold change >2, FDR<0.05 for RT&Tag; fold change >1, p<0.05 for PIRCh-seq).

We also compared our m6A RT&Tag to the one published MeRIP-seq datasets for Drosophila S2 cells[7]. In this publication, m6A peaks were called using m6A IP alignment files and the coverage (counts per million) for each of these m6A peaks was calculated using m6A IP and input samples. Using a >1.3 fold change cutoff, 1120 m6A peaks were identified, which correspond to 812 genes. However, we find that the vast majority of these m6A peaks do not reach statistical significance, leaving only 17 peaks with an FDR <0.05 (see response Figure D). This number is far fewer than what we've been able to identify with m6A RT&Tag (281 vs 17). Fewer than half (7 out of 17) of these MeRIP-seq peaks overlapped with transcripts identified by m6A RT&Tag.

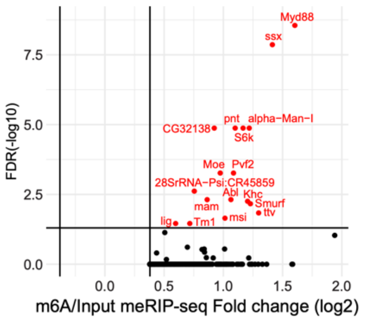

Figure D. Volcano plot showing transcripts with m6A peaks identified as enriched over input with MeRIP-seq (fold change >1.3, FDR <0.05).

Open Access This file is licensed under a Creative Commons Attribution 4.0 International License, which permits use, sharing, adaptation, distribution and reproduction in any medium or format, as long as you give appropriate credit to the original author(s) and the source, provide a link to the Creative Commons license, and indicate if changes were made. In the cases where the authors are anonymous, such as is the case for the reports of anonymous peer reviewers, author attribution should be to 'Anonymous Referee' followed by a clear attribution to the source work. The images or other third party material in this file are included in the article's Creative Commons license, unless indicated otherwise in a credit line to the material. If material is not included in the article's Creative Commons license and your intended use is not permitted by statutory regulation or exceeds the permitted use, you will need to obtain permission directly from the copyright holder. To view a copy of this license, visit http://creativecommons.org/licenses/by/4.0/.

4. Since protein-bound RNAs and transcripts within the vicinity of the protein in space are both detected by RT&Tag, is there any way to distinguish them if only interested in protein-bound RNAs?

The reviewer is correct in pointing out that RT&Tag, unlike precipitation-based methods, is a proximity labeling-based technique and can detect transcripts that are not only protein-bound but also within the vicinity of the target protein. This point is now discussed in the section pertaining to the comparison between MSL2 RT&Tag and MLE RIP-seq (pg 4, lines 147-159). Although there isn't a concrete way to distinguish between the two, proximity interactions should in theory be transient and result in weaker enrichment. The volcano plot in Fig. 2 shows roX2, which is known to directly bind to MSL2, to be a unique outlier both in fold-change and FDR. Meanwhile, the proximal transcripts found on the X-chromosome near MSL2 binding sites exhibit either low fold-change or low FDR. This point is now included in the main text (pg 4, lines 143-146).

5. In Fig. 3h, it is unclear how the rows within different groups in the heatmap are sorted, which needs to be clarified in the figure legend.

We have included clarification regarding the heatmap row sorting in the figure legend (pg 19 lines 704-706)

6. It has been reported that bivalent domains (H3K4me3 and H3K27me3) tend to coincide with TF genes expressed at low levels in mouse ES cells (PMID: 16630819). The authors find that low production of new transcripts is from silenced regions, which is not surprising to me. What biological processes are the 208 H3K27me3 RT&Tag-enriched transcripts associated with?

We have performed GO enrichment analysis for the H3K27me3 RT&Tag-enriched transcripts and indeed found them to be associated with developmental biological processes. The GO analysis is now included in Supplementary Figure 4A (see response Figure E) and is referenced in the main text (pg 5, lines 178-180). It is important, however, to make the distinction that although bivalent domains have been identified in mammalian systems, minimal overlap between H3K4me3 and H3K27me3 has been observed in *Drosophila*[8-10] (Figure 3H). The transcripts shown in Figure 3G do not come from bivalent genes, but from canonical Polycomb-silenced genes.

Open Access This file is licensed under a Creative Commons Attribution 4.0 International License, which permits use, sharing, adaptation, distribution and reproduction in any medium or format, as long as you give appropriate credit to the original author(s) and the source, provide a link to the Creative Commons license, and indicate if changes were made. In the cases where the authors are anonymous, such as is the case for the reports of anonymous peer reviewers, author attribution should be to 'Anonymous Referee' followed by a clear attribution to the source work. The images or other third party material in this file are included in the article's Creative Commons license, unless indicated otherwise in a credit line to the material. If material is not included in the article's Creative Commons license and your intended use is not permitted by statutory regulation or exceeds the permitted use, you will need to obtain permission directly from the copyright holder. To view a copy of this license, visit http://creativecommons.org/licenses/by/4.0/.

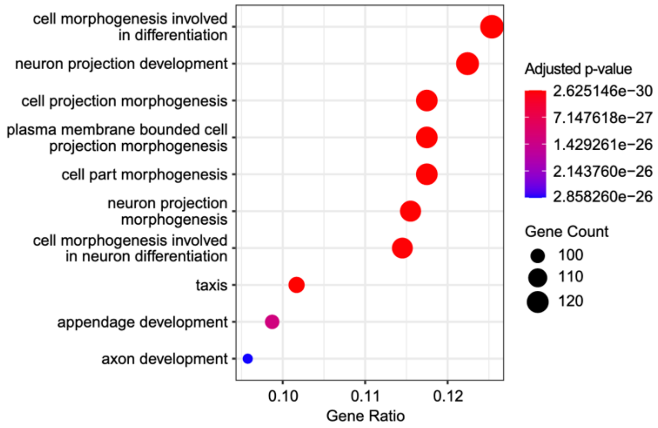

Figure E. Dot plot showing the top 10 GO biological process terms associated with H3K27me3-enriched transcripts. The dot size corresponds to the gene count and the color represents statistical significance.

7. The authors performed RNAPolII CUT&Tag using antibody of unphosphorylated RNA polymerase II that functions at early pre-initiating stage during transcription (PMID: 17079683), and the signals at gene body are hardly detected using the antibody in theory. Thus, the analysis of pause index (TSS density/gene body density) is not convincing to me.

The 8WG16 antibody we used here has previously been used by Julia Zeitlinger's lab when they first defined the pausing index[11]. Additionally, in S2 cells, we found the 8WG16 antibody to detect signal within both the TSS and the gene bodies most similarly to the S2P elongating RNA PolII targeting antibody (see response Figure F).

Open Access This file is licensed under a Creative Commons Attribution 4.0 International License, which permits use, sharing, adaptation, distribution and reproduction in any medium or format, as long as you give appropriate credit to the original author(s) and the source, provide a link to the Creative Commons license, and indicate if changes were made. In the cases where the authors are anonymous, such as is the case for the reports of anonymous peer reviewers, author attribution should be to 'Anonymous Referee' followed by a clear attribution to the source work. The images or other third party material in this file are included in the article's Creative Commons license, unless indicated otherwise in a credit line to the material. If material is not included in the article's Creative Commons license and your intended use is not permitted by statutory regulation or exceeds the permitted use, you will need to obtain permission directly from the copyright holder. To view a copy of this license, visit http://creativecommons.org/licenses/by/4.0/.

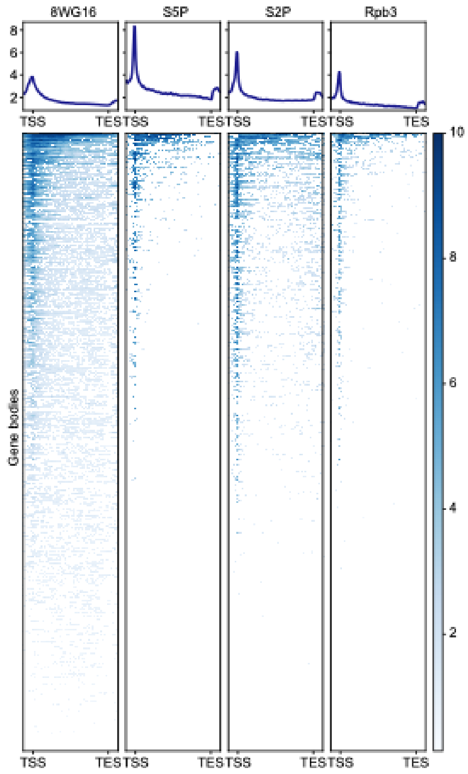

Figure F. Heatmap showing signal for unphosphorylated RNAPolII (8WG16), initiating RNAPolII (S5P), elongating RNAPolII (S2P), and total RNAPolII (Rpb3) over all gene bodies in Drosophila. Heatmaps are plotted in the order of decreasing RNAPolII signal (across all four types of RNAPolII).

8. The important link between RNAPolII pausing and the m6A RNA modification has been reported (PMID: 34297910), which cuts down the novelty of this manuscript.

We disagree. For a methods paper, we think that it is important to show that results obtained using the method are consistent with previous work, as pure novelty would lead to the suspicion that one is detecting an artifact. References ([12]) and ([13], which appeared when our paper was in review) show that the m6A writer, METTL3, modulates RNAPolII dynamics as knockdown of METTL3 resulted in decreased RNA PolII elongation. We confirmed and extended the findings of [12,13] by showing that transcripts containing the m6A modification are associated with promoter GAF binding and paused RNA polymerase II.

Open Access This file is licensed under a Creative Commons Attribution 4.0 International License, which permits use, sharing, adaptation, distribution and reproduction in any medium or format, as long as you give appropriate credit to the original author(s) and the source, provide a link to the Creative Commons license, and indicate if changes were made. In the cases where the authors are anonymous, such as is the case for the reports of anonymous peer reviewers, author attribution should be to 'Anonymous Referee' followed by a clear attribution to the source work. The images or other third party material in this file are included in the article's Creative Commons license, unless indicated otherwise in a credit line to the material. If material is not included in the article's Creative Commons license and your intended use is not permitted by statutory regulation or exceeds the permitted use, you will need to obtain permission directly from the copyright holder. To view a copy of this license, visit http://creativecommons.org/licenses/by/4.0/.

9. To really establish the regulatory relationship between m6A and RNAPolII, the authors need to simultaneously profile both molecular information in the same single cells. This may represent a major advance to the broad field of epigenetics.

Being able to simultaneously profile both m6A and RNAPolII within the same single cell would indeed be a major advance. However, this idea is outside the scope of this current manuscript which is devoted to describing a novel method and showing that it outperforms currently popular immunoprecipitation-based methods. Developing a practical single-cell multiomics method that combines RT&Tag with CUT&Tag will require future technological advances given the sparseness of chromatin-associated RNAs and the fact that single-cell RNA-seq is limited to abundant RNAs.

Reviewer #2:
Remarks to the Author:
“Profiling RNA at chromatin targets in situ by antibody-targeted tagmentation."

Khyzha et al. developed a high throughput technology to capture and amplify RNA molecules within their native chromatin or regulatory protein complexes. The authors successfully quantified RNA transcripts localized at Polycomb-targeted chromatin modification and RNA enriched with methylation modification (m6A). The key feature of this method is the elimination of crosslinking, use of Tn5 transposition at targeted RNA/cDNA heteroduplexes, and targeted amplification of localized RNA molecules from a small number of intact nuclei. This methodology allows gaining insight into RNA-guided chromatin regulation and epitranscriptomic studies. The scalability of RT&Tag in capturing RNA from its native chromatin site is an improvement compared to other RNA pulldown protocols. In addition to providing a new method, the authors generated some biological insight by finding a correlation between m6A methylation and RNAPII pausing on nascent transcripts. However, a number of shortcomings needs to be addressed.

We thank Reviewer 2 for this positive overall assessment of our method.

Specific Comments:

• The authors should compare their RT&Tag method to others, regarding cell number, the cost of reagents, and the sequencing depth, in order for readers to appreciate the extent of the advance.

We thank the reviewer for this suggestion. We now present a table (new Table 1) described in the Discussion (pg 7, line 279) comparing RT&Tag to immunoprecipitation-based methods with regard to cell number, experimental details, costs, and sequencing depth (see response Table A).

Open Access This file is licensed under a Creative Commons Attribution 4.0 International License, which permits use, sharing, adaptation, distribution and reproduction in any medium or format, as long as you give appropriate credit to the original author(s) and the source, provide a link to the Creative Commons license, and indicate if changes were made. In the cases where the authors are anonymous, such as is the case for the reports of anonymous peer reviewers, author attribution should be to 'Anonymous Referee' followed by a clear attribution to the source work. The images or other third party material in this file are included in the article’s Creative Commons license, unless indicated otherwise in a credit line to the material. If material is not included in the article’s Creative Commons license and your intended use is not permitted by statutory regulation or exceeds the permitted use, you will need to obtain permission directly from the copyright holder. To view a copy of this license, visit http://creativecommons.org/licenses/by/4.0/.

Table A. <u>Comparison of RT&Tag to immunoprecipitation-based methods.</u>

		RNA-protein		RNA-chromatin		RNA modification	
	RT&Tag	RIP-seq	CLIP-seq	PIRCh-seq	ChRIP-seq	MeRIP-seq	m6A-RIP-seq
Input Material	0.1 million	20 million	20 million	5 million	20 million	20 million	50 million
Sequencing reads	4-8 million	20 million	25 million	50 million	50 million	73 million	20 million
Time	1-2 Days	3 Days	4 Days	3 Days	3 Days	2-3 Days	2-3 Days
Cost	$50	$150	$200	$225	$200	$300	$200

• What is the effect of potential variation in reverse transcription for targeted RNA amplification?

The reviewer brings up an important point regarding variation in reverse transcription which we now discuss in the results section (pg 3, lines 91-96). To keep the nuclei samples intact, reverse transcription in RT&Tag is performed at 37˚C. The downside is that RNA with strong secondary structure may be more difficult to reverse transcribe than at higher temperatures (50˚C). Proteins bound to RNA may also interfere with the processivity of the reverse transcriptase and shorter transcripts are easier to reverse transcribe than longer ones. However, detecting signal predominantly from the 3' end of RNA minimizes the variation arising from the mentioned factors as there is no need to reverse transcribe the full RNA transcript.

• What is the spatial resolution of this method? For example, it would be interesting to quantify and compare the spread of captured RNA at regulatory regions with extended modification (e. g., H3K27me3) to compact and punctuated states.

We thank the reviewer for this interesting question. We have now measured the genomic distance between chromatin regions and the gene bodies of their enriched transcripts. This analysis was performed for compact/punctuated sites (MSL2 binding peaks) and extended modifications (H3K27me3 peaks). In both cases, 50% of the enriched transcripts are directly overlapping an MSL2 or H3K27me3 peak, and 75% of enriched transcripts are within 13,000 bp or 20,000 bp from an MSL2 or H3K27me3 peak, respectively (see response Figure G).

Open Access This file is licensed under a Creative Commons Attribution 4.0 International License, which permits use, sharing, adaptation, distribution and reproduction in any medium or format, as long as you give appropriate credit to the original author(s) and the source, provide a link to the Creative Commons license, and indicate if changes were made. In the cases where the authors are anonymous, such as is the case for the reports of anonymous peer reviewers, author attribution should be to 'Anonymous Referee' followed by a clear attribution to the source work. The images or other third party material in this file are included in the article's Creative Commons license, unless indicated otherwise in a credit line to the material. If material is not included in the article's Creative Commons license and your intended use is not permitted by statutory regulation or exceeds the permitted use, you will need to obtain permission directly from the copyright holder. To view a copy of this license, visit http://creativecommons.org/licenses/by/4.0/.

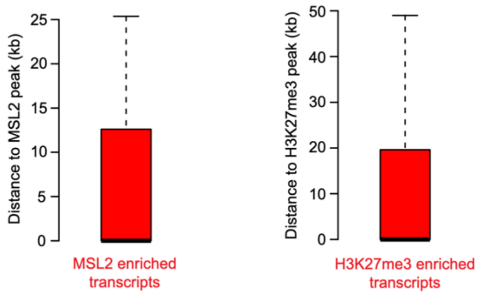

Figure G. Distance from the gene body of an MSL2 (left) or H3K27me3 (right) enriched transcripts to an MSL2 or H3K27me3 peak.

As a control, we then measured the distance between MSL2 or H3K27me3 peaks and their enriched or nonenriched transcripts. As expected, we found the distance from MSL2 or H3K27me3 peaks to enriched transcripts to be much shorter than to nonenriched transcripts (see response Figure H). This difference was much more pronounced for MSL2 peaks likely because MSL2 binding sites are sparse relative to H3K27me3 and predominantly localized to the X-chromosome (see response Figure H).

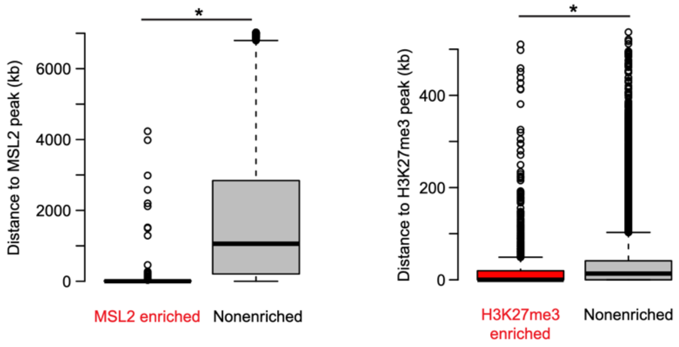

Figure H. Boxplot showing the genomic distance from the gene body of MSL2 (left) or H3K27me3 (right) enriched or nonenriched transcripts to the nearest MSL2 or H3K27me3 peak. *p<0.05.

We have now included the data pertaining to MSL2 in Supplementary Figure 4 and reference it in the text (pg 4, lines 137-139).

• While the correlation between m6A methylation and RNAPII pausing on nascent transcripts is interesting,

Open Access This file is licensed under a Creative Commons Attribution 4.0 International License, which permits use, sharing, adaptation, distribution and reproduction in any medium or format, as long as you give appropriate credit to the original author(s) and the source, provide a link to the Creative Commons license, and indicate if changes were made. In the cases where the authors are anonymous, such as is the case for the reports of anonymous peer reviewers, author attribution should be to 'Anonymous Referee' followed by a clear attribution to the source work. The images or other third party material in this file are included in the article's Creative Commons license, unless indicated otherwise in a credit line to the material. If material is not included in the article's Creative Commons license and your intended use is not permitted by statutory regulation or exceeds the permitted use, you will need to obtain permission directly from the copyright holder. To view a copy of this license, visit http://creativecommons.org/licenses/by/4.0/.

the authors have not provided evidence of function or causality. Does m6A cause pausing or does pausing cause m6A? Or is the correlation a red herring?
The reviewer poses an interesting question which we now expand upon in the discussion (pg 8, lines 312-314). Based on current literature, there is evidence to support both that pausing causes m6A and that m6A causes pausing. The Agami group showed that slowing down RNA PolII using the topoisomerase I inhibitor camptothecin or the slow elongation C4/R749H RNA PolII mutant, resulted in elevated m6A levels[14]. On the other hand, the Junion group showed that knockdown of METTL3 in Drosophila decreases RNA PolII elongation[12]. In line with this, the Shen group showed last month that METTL3 is necessary for productive RNA PolII elongation by preventing integrator binding[13]. Hence, we now speculate that there may be two-way communication between m6A and RNA PolII processivity.

Comments on figures:

Figure 2D: It might help to generate a separate figure for each experiment: the H3K27me, me6A, and MSL2 associate transcripts and compare the classification of RNA molecules between the methodologies.

We have re-done the analysis in Figure 2D for H3K27me3 and m6A RT&Tag signal and included it in a new Supplementary Figure (now Sup Figure 3B) which is referenced in the text (pg 3, line 117). As expected, we've observed a strong 3' bias in RT&Tag signal for all three groups with most of the signal falling within the last 20% of the gene length (see response Figure I).

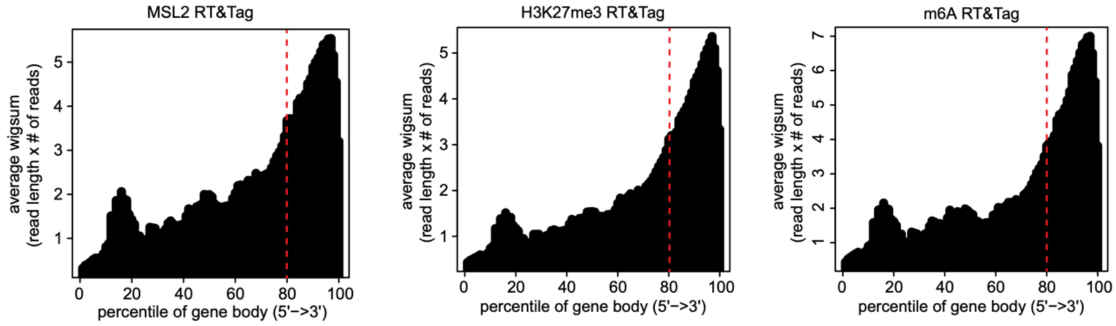

Figure I. Density plots showing the distribution of aligned MSL2 (left, n=4) H3K27me3 (middle, n=5) and m6A (right, n=3) RT&Tag reads scaled over Drosophila gene bodies.

Supplementary Figure 1: The authors provide RNA read counts obtained from the simultaneous and

Open Access This file is licensed under a Creative Commons Attribution 4.0 International License, which permits use, sharing, adaptation, distribution and reproduction in any medium or format, as long as you give appropriate credit to the original author(s) and the source, provide a link to the Creative Commons license, and indicate if changes were made. In the cases where the authors are anonymous, such as is the case for the reports of anonymous peer reviewers, author attribution should be to 'Anonymous Referee' followed by a clear attribution to the source work. The images or other third party material in this file are included in the article's Creative Commons license, unless indicated otherwise in a credit line to the material. If material is not included in the article's Creative Commons license and your intended use is not permitted by statutory regulation or exceeds the permitted use, you will need to obtain permission directly from the copyright holder. To view a copy of this license, visit http://creativecommons.org/licenses/by/4.0/.

sequential reverse transcription experiments. The experiment is informative, but the authors did not discuss the results. Additionally, they could also compare the length of RNA molecules between different conditions.

We now include an explanation of our results obtained from the simultaneous and sequential reverse transcription experiments and provided a hypothesis as to why this may occur (pg 3, lines 79-85).

We also compared the length of RNA molecules captured between simultaneous (with or without biotinylated oligo(dT)) and sequential reverse transcription experiments. Under all conditions, the regions of the RNA molecule captured by RT&Tag showed a strong 3' bias. However, with simultaneous tagmentation and reverse transcription, the 3' bias was not as strong which suggests that a longer fragment of the RNA is captured by RT&Tag (see response Figure J). These data are now included in Supplementary Figure 1C and are referenced in the text (pg 3, line 85).

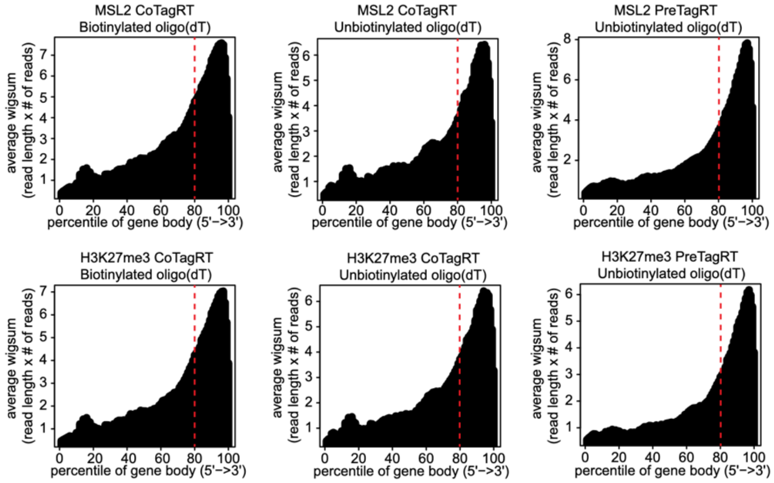

Figure J. Density plots showing the distribution of aligned MSL2 (top) and H3K27me3 (bottom) RT&Tag reads (n=2) scaled over Drosophila gene bodies for biotinylated oligo(dT) CoTagRT (left), unbiotinylated oligo(dT) CoTagRT (center), and unbiotinylated oligo(dT) preTagRT (right) RT&Tag variations.

Supplementary Figure 1A & B: The 'performance comparison' in control conditions (Co-Tag-RT and

Open Access This file is licensed under a Creative Commons Attribution 4.0 International License, which permits use, sharing, adaptation, distribution and reproduction in any medium or format, as long as you give appropriate credit to the original author(s) and the source, provide a link to the Creative Commons license, and indicate if changes were made. In the cases where the authors are anonymous, such as is the case for the reports of anonymous peer reviewers, author attribution should be to 'Anonymous Referee' followed by a clear attribution to the source work. The images or other third party material in this file are included in the article's Creative Commons license, unless indicated otherwise in a credit line to the material. If material is not included in the article's Creative Commons license and your intended use is not permitted by statutory regulation or exceeds the permitted use, you will need to obtain permission directly from the copyright holder. To view a copy of this license, visit http://creativecommons.org/licenses/by/4.0/.

Biotinylated oligo(dT)) for H3K4me27 RT&Tag is vastly different (~200 in A, compared to ~40 in B). Did they execute the control experiments similarly?

We have added clarifications regarding Supplementary Figure 1A & B in the panel labels and in the figure legends. Both experiments in Supplementary Figure 1A were performed as Co-TagRT, whereas both experiments in Supplementary Figure 1B were performed using unbiotinylated oligo(dT).

Figure 2C: Please classify captured RNA molecules from all three experiments: the H3K27me, me6A, and MSL2 associated transcripts.

We have performed the analysis in Figure 2D for H3K27me3 and m6A RT&Tag signal and included it in a new Supplementary Figure (now Sup Figure 3A) and referenced it in the text (pg 3, line 115). We found that m6A RT&Tag resulted in the highest proportion of exonic reads followed by MSL2 and then H3K27me3. The reverse pattern was observed with intronic reads whereby H3K27me3 RT&Tag resulted in the most intronic reads (see response Figure K). These results are in line with our observation that H3K27me3 RT&Tag picks up recently transcribed transcripts from chromatin regions with H3K27me3 marks and that these transcripts have not yet had a chance to undergo splicing.

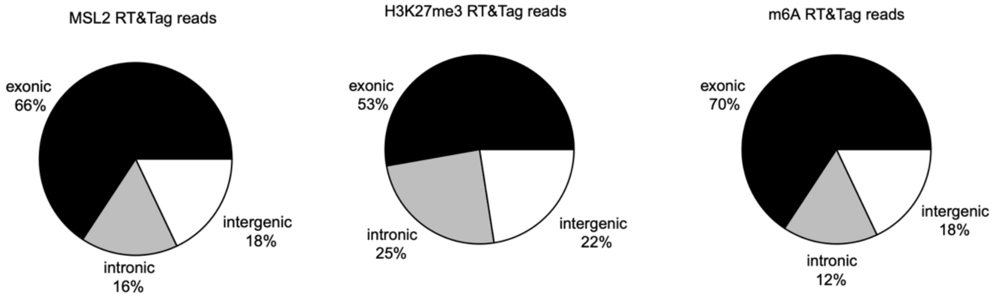

Figure K. Pie chart showing the proportion of MSL2 (left, n=4), H3K27me3 (center, n=5) and m6A (right, n=3) RT&Tag reads aligning to regions classified as either exonic, intronic, or intergenic.

Figure 3F: Please include H3K4me1 associated RT&Tag signal and overlapping regions with H3K27me, which recognize bivalent regulatory regions at distal promoter and enhancer sites.

We believe that the reviewer is inquiring about regions containing both H3K4me3 and H3K27me3 marks which have been termed as "bivalent" regions[15]. Although this phenomenon has been reported in

Open Access This file is licensed under a Creative Commons Attribution 4.0 International License, which permits use, sharing, adaptation, distribution and reproduction in any medium or format, as long as you give appropriate credit to the original author(s) and the source, provide a link to the Creative Commons license, and indicate if changes were made. In the cases where the authors are anonymous, such as is the case for the reports of anonymous peer reviewers, author attribution should be to 'Anonymous Referee' followed by a clear attribution to the source work. The images or other third party material in this file are included in the article's Creative Commons license, unless indicated otherwise in a credit line to the material. If material is not included in the article's Creative Commons license and your intended use is not permitted by statutory regulation or exceeds the permitted use, you will need to obtain permission directly from the copyright holder. To view a copy of this license, visit http://creativecommons.org/licenses/by/4.0/.

mammalian systems, minimal overlap between H3K4me3 and H3K27me3 has been observed in *Drosophila* ([8-10] and Figure 3H). Hence, we do not see the proposed experiment to add value to the manuscript.

Supplementary Figure 3: Since the MSL4 CUT&Tag signal seems highly variable (scale is 0 to 150 compared to 0 to 1000) and also H4K16ac seem comparably similar in both browser traces, I suggest providing quartile comparisons of RNA reads at MSL4 'enriched' and 'nonenriched' sites and removing H4K16Ac track, entirely.

MSL2 as part of the Drosophila Dosage Compensation Complex binds to compact high-affinity sites (HASs) along the X-chromosome. In contrast, the H4K16ac histone modification is spread out over several kilobases around HAS sites[16]. Hence, MSL2 CUT&Tag signal tends to be concentrated at a select number of sites while the H4K16ac CUT&Tag signal is diffuse. This is likely the reason as to why the MSL2 CUT&Tag signal is highly variable between the two example regions in Supplementary Figure 4 while H4K16ac isn't. We don't necessarily see this to be a problem as it is representative of a normal biological phenomenon.

Figure 5H: Since genes with lower steady-state expression tend to exhibit 'longer' RNAPII pausing, it makes sense to calculate the pausing index at m6A genes for two separate groups of highly expressed and lowly expressed genes Please see:

The reviewer brings up a valid point. We have broken down our list of m6A-enriched genes into quartiles based on their expression levels and assessed RNA PolII pausing. These data are now included in Supplementary Figure 9F and are referenced in the text (pg 7, lines 261-263). No significant differences in RNA PolII pausing were observed between the quartile groups (see response Figure L). This is likely because most m6A-enriched genes tend to have low expression levels relative to the highly expressed m6A-depleted genes (Figure 5B).

Open Access This file is licensed under a Creative Commons Attribution 4.0 International License, which permits use, sharing, adaptation, distribution and reproduction in any medium or format, as long as you give appropriate credit to the original author(s) and the source, provide a link to the Creative Commons license, and indicate if changes were made. In the cases where the authors are anonymous, such as is the case for the reports of anonymous peer reviewers, author attribution should be to 'Anonymous Referee' followed by a clear attribution to the source work. The images or other third party material in this file are included in the article's Creative Commons license, unless indicated otherwise in a credit line to the material. If material is not included in the article's Creative Commons license and your intended use is not permitted by statutory regulation or exceeds the permitted use, you will need to obtain permission directly from the copyright holder. To view a copy of this license, visit http://creativecommons.org/licenses/by/4.0/.

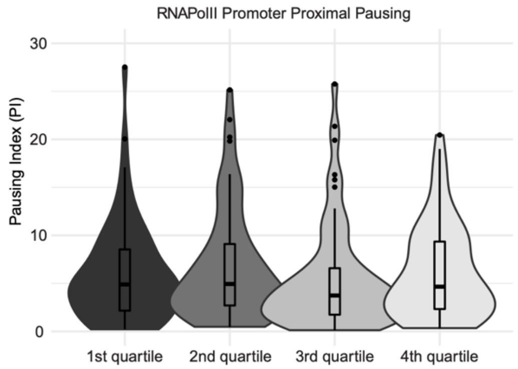

Figure L. Violin plots displaying the promoter proximal pausing index (PI) m6A-enriched transcripts broken down into quartiles based on their RNA-seq expression levels. PI was calculated by dividing the promoter (+/- 250 bp around the TSS) RNAPolII CUT&Tag signal over the gene body RNAPolII CUT&Tag signal.

Reviewer #3:
Remarks to the Author:
In this manuscript, Henikoff and colleagues present a new method called RT&TAG. This approach extends their widely used CUT&TAG technology (which maps DNA sites in proximity to specific proteins) to enable mapping of RNAs that are in proximity to a specific protein of interest in the nucleus. They show that this approach is able to recover the well-characterized dosage compensation ncRNA (roX2) when exploring the MSL complex along with other X-linked genes. They similarly show two other proof-of-concept demonstrations related to m6A methylation and polycomb associated RNAs.

Overall, the authors provide a compelling technical demonstration that the RT&TAG approach is accurate and works well in defining RNAs that are in proximity to their targeted proteins. I think this is likely to be a valuable method for the RNA and chromatin communities and am strongly in favor of its publication in Nature Methods. However, I do have concerns related to how several key points are framed and the interpretation of RT&TAG results that I believe are critical for the authors to address in the writing of the manuscript prior to publication.

We thank Reviewer 3 for this positive overall assessment of our method.

I think it is important for the authors to more clearly explain what exactly they are measuring and, equally importantly, what they are NOT measuring. The gold-standard for measuring RNA-protein interactions is UV-based CLIP. This approach uses highly stringent crosslinking (UV which only forms covalent crosslinks between direct RNA and protein contacts that occur within “zero distance) along with highly stringent

Open Access This file is licensed under a Creative Commons Attribution 4.0 International License, which permits use, sharing, adaptation, distribution and reproduction in any medium or format, as long as you give appropriate credit to the original author(s) and the source, provide a link to the Creative Commons license, and indicate if changes were made. In the cases where the authors are anonymous, such as is the case for the reports of anonymous peer reviewers, author attribution should be to 'Anonymous Referee' followed by a clear attribution to the source work. The images or other third party material in this file are included in the article’s Creative Commons license, unless indicated otherwise in a credit line to the material. If material is not included in the article’s Creative Commons license and your intended use is not permitted by statutory regulation or exceeds the permitted use, you will need to obtain permission directly from the copyright holder. To view a copy of this license, visit http://creativecommons.org/licenses/by/4.0/.

purification methods (high-salt washes and separation and purification through a denaturing SDS-PAGE gel) to map direct RNA-protein contacts. I think it is important for the authors to clearly point out that RT&TAG is NOT limited to detection of direct RNA-protein interactions. As currently written (especially when comparing the ~150 ENCODE proteins mapped by CLIP in the discussion) one could read this paper as claiming that it is a replacement for these approaches which it is clearly not.
We thank the reviewer for this suggestion. To clarify that RT&Tag is not limited to detection of RNA-protein interactions, we have now modified our schematic in Figure 1B and have revised the introduction (pg 2, lines 35-46) and discussion (pg 7, lines 270-276) accordingly.

Similarly, the authors should clearly note that what they are measuring are RNAs that are in proximity to a protein of interest not necessarily those that directly or even indirectly bind or associate with the protein. This is a distinction that leads to a major source of confusion in the RNA-protein field and therefore is critical to explicitly discuss. The distinction is that nascent RNAs are in proximity to MSL or polycomb but do not necessarily bind to them either directly or indirectly. To make this more concrete, imagine a protein that is associated with transcribed DNA (say H3K36me3), you would presumably detect RNAs with it using RT&TAG because of its proximity to nascent transcripts but this does NOT imply that H3K36me3 binds to RNA. This is by no means limited to RT&TAG (RIP-Seq also has this issue) but this distinction is important and often confusing.

The reviewer brings up a valid point which we now address in the introduction (pg 2, lines 57-58), results (pg 4, lines 153-159) and discussion (pg 7, lines 270-276) to highlight that RT&Tag is a proximity labeling tool that captures RNA within the vicinity of where Tn5 is tethered.

Finally, the authors argue that this approach specifically maps chromatin associated RNPs. It is possible that I missed this key technical point, but from my understanding of the method there is nothing that limits the associations detected to those that occur on chromatin. If I understand correctly, any RNA that is in proximity to their protein in the nucleus would be tagged whether on chromatin or nucleoplasm. If my understanding is correct, this is certainly not a problem for the method but the authors should adjust their wording to avoid implying chromatin specific mapping. If I am misunderstanding the method, the authors should clarify their description in section 1 to more explicitly explain why this approach is limited to chromatin and not nucleoplasmic associations.

The reviewer is correct in pointing out that RT&Tag is not limited to chromatin associated RNPs. We have now revised the manuscript to make this point more explicit. In our schematic in Figure 1B, we show that RT&Tag can be broadly used for capturing RNA-protein interactions. We also revised the discussion (pg 8, lines 324-327) to make it explicit that RT&Tag has potential applications that are not limited to chromatin.

Open Access This file is licensed under a Creative Commons Attribution 4.0 International License, which permits use, sharing, adaptation, distribution and reproduction in any medium or format, as long as you give appropriate credit to the original author(s) and the source, provide a link to the Creative Commons license, and indicate if changes were made. In the cases where the authors are anonymous, such as is the case for the reports of anonymous peer reviewers, author attribution should be to 'Anonymous Referee' followed by a clear attribution to the source work. The images or other third party material in this file are included in the article's Creative Commons license, unless indicated otherwise in a credit line to the material. If material is not included in the article's Creative Commons license and your intended use is not permitted by statutory regulation or exceeds the permitted use, you will need to obtain permission directly from the copyright holder. To view a copy of this license, visit http://creativecommons.org/licenses/by/4.0/.

Citations

1 Zarnegar, B. J. *et al.* irCLIP platform for efficient characterization of protein-RNA interactions. *Nat Methods* **13**, 489-492, doi:10.1038/nmeth.3840 (2016).

2 Su, R. *et al.* Global profiling of RNA-binding protein target sites by LACE-seq. *Nat Cell Biol* **23**, 664-675, doi:10.1038/s41556-021-00696-9 (2021).

3 Kaya-Okur, H. S. *et al.* CUT&Tag for efficient epigenomic profiling of small samples and single cells. *Nat Commun* **10**, 1930, doi:10.1038/s41467-019-09982-5 (2019).

4 Zhu, C. *et al.* Joint profiling of histone modifications and transcriptome in single cells from mouse brain. *Nat Methods* **18**, 283-292, doi:10.1038/s41592-021-01060-3 (2021).

5 Almeida, M., Bowness, J. S. & Brockdorff, N. The many faces of Polycomb regulation by RNA. *Curr Opin Genet Dev* **61**, 53-61, doi:10.1016/j.gde.2020.02.023 (2020).

6 Fang, J. *et al.* PIRCh-seq: functional classification of non-coding RNAs associated with distinct histone modifications. *Genome Biol* **20**, 292, doi:10.1186/s13059-019-1880-3 (2019).

7 Lence, T. *et al.* m(6)A modulates neuronal functions and sex determination in Drosophila. *Nature* **540**, 242-247, doi:10.1038/nature20568 (2016).

8 Kharchenko, P. V. *et al.* Comprehensive analysis of the chromatin landscape in Drosophila melanogaster. *Nature* **471**, 480-485, doi:10.1038/nature09725 (2011).

9 Gan, Q. *et al.* Monovalent and unpoised status of most genes in undifferentiated cell-enriched Drosophila testis. *Genome Biol* **11**, R42, doi:10.1186/gb-2010-11-4-r42 (2010).

10 Schertel, C. *et al.* A large-scale, in vivo transcription factor screen defines bivalent chromatin as a key property of regulatory factors mediating Drosophila wing development. *Genome Res* **25**, 514-523, doi:10.1101/gr.181305.114 (2015).

11 Chen, K. *et al.* A global change in RNA polymerase II pausing during the Drosophila midblastula transition. *Elife* **2**, e00861, doi:10.7554/eLife.00861 (2013).

12 Akhtar, J. *et al.* m(6)A RNA methylation regulates promoter- proximal pausing of RNA polymerase II. *Mol Cell* **81**, 3356-3367 e3356, doi:10.1016/j.molcel.2021.06.023 (2021).

13 Xu, W. *et al.* Dynamic control of chromatin-associated m(6)A methylation regulates nascent RNA synthesis. *Mol Cell*, doi:10.1016/j.molcel.2022.02.006 (2022).

14 Slobodin, B. *et al.* Transcription Impacts the Efficiency of mRNA Translation via Co-transcriptional N6-adenosine Methylation. *Cell* **169**, 326-337 e312, doi:10.1016/j.cell.2017.03.031 (2017).

15 Bernstein, B. E. *et al.* A bivalent chromatin structure marks key developmental genes in embryonic stem cells. *Cell* **125**, 315-326, doi:10.1016/j.cell.2006.02.041 (2006).

Open Access This file is licensed under a Creative Commons Attribution 4.0 International License, which permits use, sharing, adaptation, distribution and reproduction in any medium or format, as long as you give appropriate credit to the original author(s) and the source, provide a link to the Creative Commons license, and indicate if changes were made. In the cases where the authors are anonymous, such as is the case for the reports of anonymous peer reviewers, author attribution should be to 'Anonymous Referee' followed by a clear attribution to the source work. The images or other third party material in this file are included in the article's Creative Commons license, unless indicated otherwise in a credit line to the material. If material is not included in the article's Creative Commons license and your intended use is not permitted by statutory regulation or exceeds the permitted use, you will need to obtain permission directly from the copyright holder. To view a copy of this license, visit http://creativecommons.org/licenses/by/4.0/.

16 Conrad, T. & Akhtar, A. Dosage compensation in Drosophila melanogaster: epigenetic fine-tuning of chromosome-wide transcription. *Nat Rev Genet* **13**, 123-134, doi:10.1038/nrg3124 (2012).

Decision Letter, first revision:

Subject: AIP Decision on Manuscript NMETH-A48191A
Message:

Our ref: NMETH-A48191A

12th Jul 2022

Dear Steve,

Thank you for your letter detailing how you would respond to the reviewer#1's concerns regarding your revised Article, "Profiling RNA at chromatin targets in situ by antibody-targeted tagmentation". After careful consideration, we have decided that we would be happy in principle to publish it in Nature Methods, pending minor revisions to satisfy the referees' final requests as outlined in your response letter and to comply with our editorial and formatting guidelines.

We are now performing detailed checks on your paper and will send you a checklist detailing our editorial and formatting requirements in about a week. Please do not upload the final materials and make any revisions until you receive this additional information from us.

TRANSPARENT PEER REVIEW
Nature Methods offers a transparent peer review option for new original research manuscripts submitted from 17th February 2021. We encourage increased transparency in peer review by publishing the reviewer comments, author rebuttal letters and editorial decision letters if the authors agree. Such peer review material is made available as a supplementary peer review file. Please state in the cover letter ‘I wish to participate in transparent peer review’ if you want to opt in, or ‘I do not wish to participate in transparent peer review’ if you don’t. Failure to state your preference will result in delays in accepting your manuscript for publication.
Please note: we allow redactions to authors’ rebuttal and reviewer comments in the interest of confidentiality. If you are concerned about the release of confidential data, please let us know specifically what information you would like to have removed. Please note that we cannot incorporate

Open Access This file is licensed under a Creative Commons Attribution 4.0 International License, which permits use, sharing, adaptation, distribution and reproduction in any medium or format, as long as you give appropriate credit to the original author(s) and the source, provide a link to the Creative Commons license, and indicate if changes were made. In the cases where the authors are anonymous, such as is the case for the reports of anonymous peer reviewers, author attribution should be to 'Anonymous Referee' followed by a clear attribution to the source work. The images or other third party material in this file are included in the article’s Creative Commons license, unless indicated otherwise in a credit line to the material. If material is not included in the article’s Creative Commons license and your intended use is not permitted by statutory regulation or exceeds the permitted use, you will need to obtain permission directly from the copyright holder. To view a copy of this license, visit http://creativecommons.org/licenses/by/4.0/.

redactions for any other reasons. Reviewer names will be published in the peer review files if the reviewer signed the comments to authors, or if reviewers explicitly agree to release their name. For more information, please refer to our FAQ page.

Thank you again for your interest in Nature Methods Please do not hesitate to contact me if you have any questions.

Best regards,
Lei

Lei Tang, Ph.D.
Senior Editor
Nature Methods

ORCID
IMPORTANT: Non-corresponding authors do not have to link their ORCIDs but are encouraged to do so. Please note that it will not be possible to add/modify ORCIDs at proof. Thus, please let your co-authors know that if they wish to have their ORCID added to the paper they must follow the procedure described in the following link prior to acceptance:
https://www.springernature.com/gp/researchers/orcid/orcid-for-nature-research

Reviewer #1 (Remarks to the Author):

I recommend that this manuscript be rejected without further revision. Most of my concerns in this revision have not been satisfactorily addressed by experiments except for plain clarifications. Overall, the authors have insufficiently demonstrated the performance of RT&Tag in comparison with existing methods in this revision as both review #2 and I have pointed out. Major shortcomings in the first-version manuscript are still present. Most importantly, the robustness of RT&Tag and biological findings were not fully validated by any experiment at all, representing tremendous caveats in this method.
Specific comments:
1. To benchmark the performance of such kind of new epigenetic method, it would be important to perform experiments on varying cell numbers based on widely-used samples (not just drosophila cells) to make a full and straightforward comparison with other methods. In this revision, the authors did not perform RT&Tag experiments in mammalian cells/tissues to compare the data quality with golden

Open Access This file is licensed under a Creative Commons Attribution 4.0 International License, which permits use, sharing, adaptation, distribution and reproduction in any medium or format, as long as you give appropriate credit to the original author(s) and the source, provide a link to the Creative Commons license, and indicate if changes were made. In the cases where the authors are anonymous, such as is the case for the reports of anonymous peer reviewers, author attribution should be to 'Anonymous Referee' followed by a clear attribution to the source work. The images or other third party material in this file are included in the article's Creative Commons license, unless indicated otherwise in a credit line to the material. If material is not included in the article's Creative Commons license and your intended use is not permitted by statutory regulation or exceeds the permitted use, you will need to obtain permission directly from the copyright holder. To view a copy of this license, visit http://creativecommons.org/licenses/by/4.0/.

standard datasets, and therefore I still do not believe that RT&Tag achieves significant improvement over existing methods.
2. The sensitivity of the current method seems to be low due to the limited transcripts detected, and (perhaps as a result) most of analyses are intentionally directed toward nominating the CUT&Tag signals at RT&Tag enriched/non-enriched regions, but not directly showing the abundance of RT&Tag transcripts.
3. In general, many results are not clearly presented. For example, experimental results such as gel pictures of different conditions in the protocol optimization are lacking.
4. From revised Figure A, such a low number of differentially enriched transcripts between H3K27me3 RT&Tag and m6A RT&Tag indicate that RT&Tag have a detection bias toward high-abundant transcripts with low sensitivity of transcripts related to biological processes.
5. I am not convinced by the authors' reply in response Figure E when it comes to the rationale of performing H3K27me3 RT&Tag as a proof-of-concept for a new method. To demonstrate the wide application in biological questions, one should at least demonstrate how RT&Tag is used to dissect the relationship between multiple histone modifications (H3K36me3, gene body; H3K4me1 and H3K27ac, enhancer marks) and associated transcriptions.
6. 'The only available PIRCh-seq dataset is from the original PIRCh-seq manuscript which was done in mouse and ours in Drosophila, so a direct comparison is not feasible' —— this is unconvincing to me. Even direct comparison is hard among different datasets, one can at least analyze those data using the same pipeline and statistic index for precision evaluation. In some cases, it would be essential to perform RT&Tag in mouse and generate comparable datasets.
7. Furthermore, due to the lack of RT&Tag experiments in mouse for comparison, it is unclear whether RT&Tag method is sensitive and effective in mammalian cells.
8. There is no experimental validation of the differentially enriched transcripts in vivo. It makes it impossible to assess whether the signals captured by RT&Tag are true or just from technical noises.
9. I disagree with the viewpoint 'For a methods paper, we think that it is important to show that results obtained using the method are consistent with previous work, as pure novelty would lead to the suspicion that one is detecting an artifact'. It is a key to place emphasis on the effectiveness and robustness of the method in a method paper, but also necessary to tell the readers what can be uniquely found with this new method, otherwise unattainable for previous studies in other methods.
10. The analyses on RT&Tag data in this manuscript largely relied on independent CUT&Tag data on corresponding marks, making it obscure to assess the power of RT&Tag independently.
11. When exploring the factors involved in regulating m6A enrichment, why did the authors decide to look at Hsp70 gene here in the first place. In this context, it might be of interest to examine candidate genes participating in RNAPolII pausing and then focus on specific genes.

Open Access This file is licensed under a Creative Commons Attribution 4.0 International License, which permits use, sharing, adaptation, distribution and reproduction in any medium or format, as long as you give appropriate credit to the original author(s) and the source, provide a link to the Creative Commons license, and indicate if changes were made. In the cases where the authors are anonymous, such as is the case for the reports of anonymous peer reviewers, author attribution should be to 'Anonymous Referee' followed by a clear attribution to the source work. The images or other third party material in this file are included in the article's Creative Commons license, unless indicated otherwise in a credit line to the material. If material is not included in the article's Creative Commons license and your intended use is not permitted by statutory regulation or exceeds the permitted use, you will need to obtain permission directly from the copyright holder. To view a copy of this license, visit http://creativecommons.org/licenses/by/4.0/.

12. If indeed the current method would be capable of generating high-quality data reflecting the enrichment of RNA molecules, we are expecting to see RT&Tag could be achieved at single-cell or close resolution for detecting RNA-protein, RNA-chromatin or RNA modification. However, it seems impossible, on the basis of the bad performance of RT&Tag for 5k cells in this revision.
13. The authors made a table to compare RT&Tag to immunoprecipitation-based methods from the aspects of time and cost in this revision, which is far from enough and rather circumferential. The community would be more interested in how RT&tag outperforms current methods in data quality. The global evaluation of RT&Tag with other methods in data quality is lacking here.
14. From what I can tell, this update seems to simply improve the writing and plain clarification, and does not represent a departure from other current methods.
15. In the current revision, the analyses shown in the whole manuscript merely focus on the identification of enriched transcripts at chromatin targets. The authors do not provide an analytical framework to deeply mine the data. The function of these transcripts is not convincingly explored as well.
16. The authors argue that simultaneously profiling both m6A and RNAPolII is outside the scope of this current manuscript. However, lack of joint RNAPolII molecule information in the current form of this method always requires separate RNAPolII experiments, making it impossible to be used in some scarce samples or even single cells.
Therefore, in addition to the insufficiency in demonstration of the robustness of this method, the novelty of this method and biological insights presented here are not a sufficiently high standard for me to recommend publication by Nature Methods.

Reviewer #2 (Remarks to the Author):

The authors have addressed most of my original concerns. The introduction is improved with more details about the method and new citations are added. There are also improvements to the figures, with more in-depth analysis for RNA classification enrichment and methodology performance. The new methodology will be useful to the epigenomics community and I can now recommend publication once a few remaining minor concerns are addressed.

1. Supplementary Fig. 6B and C: to assess the reproducibility in performance of the protocol, they applied various cell numbers, each in two technical replicates, and measured the genome-wide RNA enrichment for H3K27me3-tagged regions. The paper's reproducibility measurement is an excellent

Open Access This file is licensed under a Creative Commons Attribution 4.0 International License, which permits use, sharing, adaptation, distribution and reproduction in any medium or format, as long as you give appropriate credit to the original author(s) and the source, provide a link to the Creative Commons license, and indicate if changes were made. In the cases where the authors are anonymous, such as is the case for the reports of anonymous peer reviewers, author attribution should be to 'Anonymous Referee' followed by a clear attribution to the source work. The images or other third party material in this file are included in the article's Creative Commons license, unless indicated otherwise in a credit line to the material. If material is not included in the article's Creative Commons license and your intended use is not permitted by statutory regulation or exceeds the permitted use, you will need to obtain permission directly from the copyright holder. To view a copy of this license, visit http://creativecommons.org/licenses/by/4.0/.

improvement, but the error bars reveal large variability, and the n is just 2. With a sample size of just 2, how were error bars determined?
2. Another strange result from this experiment is that although the reproducibility of normalized signal among three libraries is good (Supplementary Fig. 6B-top), the reads for each RNA (CPM) between the libraries are puzzling. The CPM drops when the cell number is higher. The authors should explain this.
3. Surprisingly, in Supplementary Fig. 6B-bottom, the genome browser screen shows the reverse pattern of what they show in the bar plot. Please explain?

-- Jeannie Lee

Reviewer #3 (Remarks to the Author):

My main concerns with the initial manuscript were about the nature of the associations detected. In the revised manuscript, the authors did a nice job clearly explaining what they are measuring and have addressed my comments. I remain enthusiastic about this method and would be happy to see this published in Nature Methods.

Author Rebuttal, first revision:

Reviewer #1:

Remarks to the Author:

I recommend that this manuscript be rejected without further revision. Most of my concerns in this revision have not been satisfactorily addressed by experiments except for plain clarifications. Overall, the authors have insufficiently demonstrated the performance of RT&Tag in comparison with existing methods in this revision as both review #2 and I have pointed out. Major shortcomings in the first-version manuscript are still present. Most importantly, the robustness of RT&Tag and biological findings were not fully validated by any experiment at all, representing tremendous caveats in this method.

Specific comments:

1. To benchmark the performance of such kind of new epigenetic method, it would be important to perform experiments on varying cell numbers based on widely-used samples (not just drosophila cells) to make a full and straightforward comparison with other methods.

Open Access This file is licensed under a Creative Commons Attribution 4.0 International License, which permits use, sharing, adaptation, distribution and reproduction in any medium or format, as long as you give appropriate credit to the original author(s) and the source, provide a link to the Creative Commons license, and indicate if changes were made. In the cases where the authors are anonymous, such as is the case for the reports of anonymous peer reviewers, author attribution should be to 'Anonymous Referee' followed by a clear attribution to the source work. The images or other third party material in this file are included in the article's Creative Commons license, unless indicated otherwise in a credit line to the material. If material is not included in the article's Creative Commons license and your intended use is not permitted by statutory regulation or exceeds the permitted use, you will need to obtain permission directly from the copyright holder. To view a copy of this license, visit http://creativecommons.org/licenses/by/4.0/.

As per Reviewer 1's initial request, we performed RT&Tag using varying cell numbers and provided these data in Supplementary Figures 6 and 8. Drosophila S2 cells have been widely used (>1600 Pubmed citations) as a model system for decades for studies such as ours. The molecular biology of heat shock was first elucidated in Drosophila S2 cells (McKenzie, *et al.*, PMID: 805422, 1975), and studies of the heat shock response in S2 cells led to the first description of chromatin immunoprecipitation (Gilmour and Lis, PMID: 3018544, 1985), and to the discovery of RNA Polymerase II pausing, also in S2 cells (Gilmour and Lis PMID: 3099167, 1986).

In this revision, the authors did not perform RT&Tag experiments in mammalian cells/tissues to compare the data quality with golden standard datasets, and therefore I still do not believe that RT&Tag achieves significant improvement over existing methods.

None of the three reviewers requested that we perform RT&Tag experiments in mammalian cells or tissues. In the Supplementary Figure 5 of the revised manuscript, we compared our MSL2 RT&Tag data to a standard method in the field, RIP-seq. We showed that RT&Tag achieved comparable transcript enrichment but using 500-fold fewer S2 cells and one-fourth the sequencing reads. We believe that this warrants a significant improvement over the existing methods.

2. The sensitivity of the current method seems to be low due to the limited transcripts detected, and (perhaps as a result) most of analyses are intentionally directed toward nominating the CUT&Tag signals at RT&Tag enriched/non-enriched regions, but not directly showing the abundance of RT&Tag transcripts.

There was no intention of leaving out data regarding transcript abundance, nor were such transcript abundance data omitted. The abundance of H3K27me3- and m6A-enriched transcripts was presented in the manuscript as counts per million (CPM) measured using RNA-seq in Figure 3E and Figure 5B, respectively.

3. In general, many results are not clearly presented. For example, experimental results such as gel pictures of different conditions in the protocol optimization are lacking.

Gel pictures are crude intermediate bulk representations of length profiles. They may be useful for users of the method to tell whether a successful Illumina sequencing library was generated and whether that library is worth sequencing. For that reason, we presented a representative gel picture of a successful library in the manuscript (Figure 2B). We do not see the utility of including gel pictures

Open Access This file is licensed under a Creative Commons Attribution 4.0 International License, which permits use, sharing, adaptation, distribution and reproduction in any medium or format, as long as you give appropriate credit to the original author(s) and the source, provide a link to the Creative Commons license, and indicate if changes were made. In the cases where the authors are anonymous, such as is the case for the reports of anonymous peer reviewers, author attribution should be to 'Anonymous Referee' followed by a clear attribution to the source work. The images or other third party material in this file are included in the article's Creative Commons license, unless indicated otherwise in a credit line to the material. If material is not included in the article's Creative Commons license and your intended use is not permitted by statutory regulation or exceeds the permitted use, you will need to obtain permission directly from the copyright holder. To view a copy of this license, visit http://creativecommons.org/licenses/by/4.0/.

for all optimization experiments in Supplementary Figure S1 as direct measure of transcript enrichment is far more telling of RT&Tag performance.

4. From revised Figure A, such a low number of differentially enriched transcripts between H3K27me3 RT&Tag and m6A RT&Tag indicate that RT&Tag have a detection bias toward high-abundant transcripts with low sensitivity of transcripts related to biological processes.

We do not agree that RT&Tag has a detection bias towards high-abundance transcripts as it was able to detect enriched transcripts over a wide dynamic range. Based on RNA-seq, H3K27me3 enriched transcript are lowly expressed with a median of 16.6 CPM with transcripts from the hox cluster being present at <1 CPM. M6A enriched transcripts are moderately abundant with a median of 192 CPM while the MSL2 enriched *roX2* transcript is at 251 CPM. We also did not observe non-specific enrichment of highly abundant transcripts such as ribosomal protein genes (>3500 CPM) for any of our tested conditions.

5. I am not convinced by the authors' reply in response Figure E when it comes to the rationale of performing H3K27me3 RT&Tag as a proof-of-concept for a new method.

H3K27me3 RT&Tag was performed based on an extensive literature implicating RNA in heterochromatin regulation (*e.g.* Zhao *et al.,* PMID:18974356, 2008). The GO analysis performed in Response Figure E was as per recommendation of Reviewer #1 and is unrelated to our rationale for performing H3K27me3 RT&Tag.

To demonstrate the wide application in biological questions, one should at least demonstrate how RT&Tag is used to dissect the relationship between multiple histone modifications (H3K36me3, gene body; H3K4me1 and H3K27ac, enhancer marks) and associated transcriptions.

We disagree with the Reviewer on this point. There is a vast amount of literature regarding the role of RNA in regulating H3K27me3-marked heterochromatin, and the functional targets are well documented. Profiling RNA transcripts localized to heterochromatin is an important biological question and is of great interest in the broader epigenetics field. We do not expect that profiling H3K36me3 will yield fruitful results as this mark is simply associated with highly active transcription and we would not expect to find anything associated with it other than nascent transcripts. As for

Open Access This file is licensed under a Creative Commons Attribution 4.0 International License, which permits use, sharing, adaptation, distribution and reproduction in any medium or format, as long as you give appropriate credit to the original author(s) and the source, provide a link to the Creative Commons license, and indicate if changes were made. In the cases where the authors are anonymous, such as is the case for the reports of anonymous peer reviewers, author attribution should be to 'Anonymous Referee' followed by a clear attribution to the source work. The images or other third party material in this file are included in the article's Creative Commons license, unless indicated otherwise in a credit line to the material. If material is not included in the article's Creative Commons license and your intended use is not permitted by statutory regulation or exceeds the permitted use, you will need to obtain permission directly from the copyright holder. To view a copy of this license, visit http://creativecommons.org/licenses/by/4.0/.

H3K4me1 and H3K27ac marks, we would expect to find enhancer RNAs which are predominantly non-polyadenylated and would not be picked up by RT&Tag.

6. 'The only available PIRCh-seq dataset is from the original PIRCh-seq manuscript which was done in mouse and ours in Drosophila, so a direct comparison is not feasible' —— this is unconvincing to me. Even direct comparison is hard among different datasets, one can at least analyze those data using the same pipeline and statistic index for precision evaluation. In some cases, it would be essential to perform RT&Tag in mouse and generate comparable datasets.

In the rebuttal letter Figure C, we had performed a direct comparison of the number of noncoding transcripts found to be associated with H3K27me3 using RT&Tag and PIRCh-seq. Specifically, we found that H3K27me3 RT&Tag detects more noncoding RNAs associated with H3K27me3 than PIRCh-seq (164 vs <70), with 50 times fewer cells and 7 times fewer sequencing reads.

7. Furthermore, due to the lack of RT&Tag experiments in mouse for comparison, it is unclear whether RT&Tag method is sensitive and effective in mammalian cells.

As stated in response to comment #1, Drosophila is a widely used model that is especially appropriate for evaluating chromatin-based genomics methods, including the very first chromatin immunoprecipitation study (Gilmour and Lis, PMID: 3018544, 1985). Furthermore, CUT&Tag has been applied to many different model and non-model organisms, with >500 citations to our 2019 *Nature Communications* paper (Kaya-Okur, *et al.,* PMID: 31036827) that first described the method. Given that RT&Tag is based on CUT&Tag, we do not expect there to be issues translating RT&Tag to other organisms and cell types.

8. There is no experimental validation of the differentially enriched transcripts in vivo. It makes it impossible to assess whether the signals captured by RT&Tag are true or just from technical noises.

This comment is unwarranted as we have taken multiple steps to validate our findings:

1) **First, as a proof-of-concept, we recapitulated the interaction between MSL2 and roX2. This interaction is well established and is frequently used for validation in technique development (for example ChAR-seq, PMID: 29648534).**
2) **We performed MSL2 CUT&Tag to show that other transcripts identified using MSL2 RT&Tag were due to MSL2 binding near their sites of transcription.**

Open Access This file is licensed under a Creative Commons Attribution 4.0 International License, which permits use, sharing, adaptation, distribution and reproduction in any medium or format, as long as you give appropriate credit to the original author(s) and the source, provide a link to the Creative Commons license, and indicate if changes were made. In the cases where the authors are anonymous, such as is the case for the reports of anonymous peer reviewers, author attribution should be to 'Anonymous Referee' followed by a clear attribution to the source work. The images or other third party material in this file are included in the article's Creative Commons license, unless indicated otherwise in a credit line to the material. If material is not included in the article's Creative Commons license and your intended use is not permitted by statutory regulation or exceeds the permitted use, you will need to obtain permission directly from the copyright holder. To view a copy of this license, visit http://creativecommons.org/licenses/by/4.0/.

3) **We showed that H3K27me3 RT&Tag identified the Hox genes, which are known to be within H3K27me3 marked Polycomb domains.**
4) **We experimentally validated our m6A-enriched transcripts by knocking down METTL3 (the writer of m6A) and showing these transcripts to have decreased enrichment for m6A.**

9. I disagree with the viewpoint 'For a methods paper, we think that it is important to show that results obtained using the method are consistent with previous work, as pure novelty would lead to the suspicion that one is detecting an artifact'. It is a key to place emphasis on the effectiveness and robustness of the method in a method paper, but also necessary to tell the readers what can be uniquely found with this new method, otherwise unattainable for previous studies in other methods.

Contrary to Reviewer #1's assertion, RT&Tag provides new insights into diverse biological phenomena of interest because of its unique features. For example:

1) **RT&Tag captured proximity interactions unlike immunoprecipitation-based methods. This point was made clear in the comparison of our MSL2 RT&Tag with published RIP-seq data (Supplementary Figure 5).**
2) **RT&Tag captured *in vivo* interactions without crosslinking, unlike current immunoprecipitation-based methods, which require extensive cross-linking that masks many epitopes from antibody binding.**
3) **RT&Tag detected small amounts of nascent transcripts at H3K27me3-marked heterochromatin which previous methods failed to detect.**
4) **RT&Tag identified 208 transcripts that are associated with H3K27me3-marked heterochromatin but are transcribed outside of it. This list could be of interest to the epigenetics community.**
5) **RT&Tag demonstrated that unlike in mammals, m6A is not involved in Hsp70 regulation during heat shock in Drosophila.**
6) **RT&Tag uncovered a surprising discrepancy between promoter METTL3 binding and deposition of m6A to METTL3 promoter-bound genes.**
7) **RT&Tag confirmed and expanded upon the interplay between m6A and RNAPolII pausing. In our manuscript we show that RNAPolII pausing is associated with the deposition of m6A and not simply METTL3 binding.**

10. The analyses on RT&Tag data in this manuscript largely relied on independent CUT&Tag data on corresponding marks, making it obscure to assess the power of RT&Tag independently.

Open Access This file is licensed under a Creative Commons Attribution 4.0 International License, which permits use, sharing, adaptation, distribution and reproduction in any medium or format, as long as you give appropriate credit to the original author(s) and the source, provide a link to the Creative Commons license, and indicate if changes were made. In the cases where the authors are anonymous, such as is the case for the reports of anonymous peer reviewers, author attribution should be to 'Anonymous Referee' followed by a clear attribution to the source work. The images or other third party material in this file are included in the article's Creative Commons license, unless indicated otherwise in a credit line to the material. If material is not included in the article's Creative Commons license and your intended use is not permitted by statutory regulation or exceeds the permitted use, you will need to obtain permission directly from the copyright holder. To view a copy of this license, visit http://creativecommons.org/licenses/by/4.0/.

The described genomic targets of MSL2 and H3K27me3 agree between ChIP and CUT&Tag methods. Thus, we do not consider this point to be a limitation of RT&Tag. The purpose of RT&Tag is to identify transcripts that are in proximity of an epitope of interest, which it achieves. CUT&Tag was then used to characterize the chromatin features of RT&Tag enriched transcripts over their gene bodies or around their transcriptional start sites. The two techniques serve different purposes with RT&Tag being to identify transcripts and CUT&Tag for additional characterization.

11. When exploring the factors involved in regulating m6A enrichment, why did the authors decide to look at Hsp70 gene here in the first place. In this context, it might be of interest to examine candidate genes participating in RNAPolII pausing and then focus on specific genes.

The phenomenon of RNAPolII pausing was first discovered at the *Hsp70* genes in Drosophila cell culture (PMID: 3136931). The heat shock response in Drosophila is a well-characterized phenomenon that our lab has used in the past for studying chromatin- and transcription-based mechanisms (*e.g.* Teves & Henikoff, PMID: 22085965, 2011), which made it an attractive system to apply our current method to. We specifically looked at the *Hsp70* genes as they experience an overwhelming increase in RNAPolII binding during heat shock. Given that RNAPolII binding is correlated with METTL3 binding, we wanted to see whether this METTL3 recruitment would result in deposition of m6A on the Hsp70 transcripts. To our surprise it did not, suggesting that other factors such as METTL3 binding dynamics might be at play. We documented the genome-wide association between RNAPolII pausing and m6A transcript enrichment on lines 254-265 in the revised manuscript.

12. If indeed the current method would be capable of generating high-quality data reflecting the enrichment of RNA molecules, we are expecting to see RT&Tag could be achieved at single-cell or close resolution for detecting RNA-protein, RNA-chromatin or RNA modification. However, it seems impossible, on the basis of the bad performance of RT&Tag for 5k cells in this revision.

RT&Tag compares favorably to other methods. The reviewer has cited LACE-seq as working in single-cells, but close examination reveals LACE-seq data to be of very poor quality when working with fewer than 1000 HeLa cells (Response Figure A below). At best, the quality of LACE-seq data with 1000 HeLa cells is comparable to that of RT&Tag with 5000 S2 cells. Performance is comparable between the two methods, despite the fact that Drosophila cells are much smaller than HeLa cells and contain less RNA, and that RT&Tag is limited to chromatin-proximal RNAs, whereas all nuclear and cytoplasmic RNAs are interrogated by LACE-seq.

Open Access This file is licensed under a Creative Commons Attribution 4.0 International License, which permits use, sharing, adaptation, distribution and reproduction in any medium or format, as long as you give appropriate credit to the original author(s) and the source, provide a link to the Creative Commons license, and indicate if changes were made. In the cases where the authors are anonymous, such as is the case for the reports of anonymous peer reviewers, author attribution should be to 'Anonymous Referee' followed by a clear attribution to the source work. The images or other third party material in this file are included in the article's Creative Commons license, unless indicated otherwise in a credit line to the material. If material is not included in the article's Creative Commons license and your intended use is not permitted by statutory regulation or exceeds the permitted use, you will need to obtain permission directly from the copyright holder. To view a copy of this license, visit http://creativecommons.org/licenses/by/4.0/.

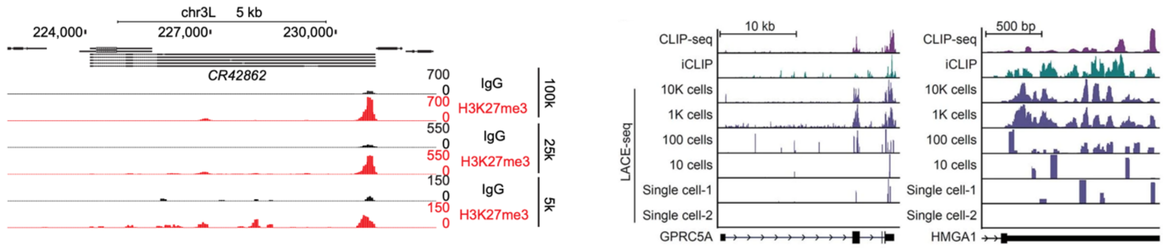

Response Figure A. RT&Tag data showing the distribution of IgG and H3K27me3 RT&Tag signal generated using either 100k, 25k, or 5k Drosophila S2 nuclei (left, Supplementary Figure 6B). LACE-seq data showing the distribution of reads using varying number of HeLa cells (right, PMID: 34108658, Supplementary Figure 1H).

13. The authors made a table to compare RT&Tag to immunoprecipitation-based methods from the aspects of time and cost in this revision, which is far from enough and rather circumferential. The community would be more interested in how RT&tag outperforms current methods in data quality. The global evaluation of RT&Tag with other methods in data quality is lacking here.

The table comparing RT&Tag to immunoprecipitation-based methods was added to the manuscript based on a comment from Reviewer #2, who suggested that such a table would be of high interest to the community. The categories in the table (Input material, sequencing reads, time and cost) were exactly the ones suggested by Reviewer #2. We had also added the global evaluation of RT&Tag as per Reviewer 1's request (Supplementary Table 7). However, other comparisons to current methods were not requested during initial revisions, and we don't think it adds value to what was already presented in the revised manuscript.

14. From what I can tell, this update seems to simply improve the writing and plain clarification, and does not represent a departure from other current methods.

This assertion is not true. We had added the following new data and analyses :

- **Performed new experiments to show the performance of RT&Tag with decreasing number of nuclei.**
- **Performed a new analysis comparing MSL2 RT&Tag and published RIP-seq data.**
- **Performed a new analysis showing the distance between MSL2 CUT&Tag binding peaks and the transcripts picked up by MSL2 RT&Tag.**
- **Performed a new analysis comparing RT&Tag gene coverage for reverse transcription condition optimization.**
- **Performed a new Gene Ontology (GO) analysis showing the significant GO categories of H3K27me3 RT&Tag enriched transcripts.**

Open Access This file is licensed under a Creative Commons Attribution 4.0 International License, which permits use, sharing, adaptation, distribution and reproduction in any medium or format, as long as you give appropriate credit to the original author(s) and the source, provide a link to the Creative Commons license, and indicate if changes were made. In the cases where the authors are anonymous, such as is the case for the reports of anonymous peer reviewers, author attribution should be to 'Anonymous Referee' followed by a clear attribution to the source work. The images or other third party material in this file are included in the article's Creative Commons license, unless indicated otherwise in a credit line to the material. If material is not included in the article's Creative Commons license and your intended use is not permitted by statutory regulation or exceeds the permitted use, you will need to obtain permission directly from the copyright holder. To view a copy of this license, visit http://creativecommons.org/licenses/by/4.0/.

- **Performed a new analysis calculating the RNAPolII Pausing Index of m6A-enriched transcripts depending on their transcript abundance.**

15. In the current revision, the analyses shown in the whole manuscript merely focus on the identification of enriched transcripts at chromatin targets. The authors do not provide an analytical framework to deeply mine the data.

We do deeply mine the data. After identifying H3K27me3 enriched transcripts, we have characterized them based on transcript abundance, pathway analysis, and histone mark levels over their gene bodies. We then sorted these transcripts into those that are likely recently transcribed from heterochromatin and those that are transcribed outside of heterochromatin and are potentially actively retained on chromatin. We believe that this analytical framework will be the most useful for users wanting to profile chromatin-associated transcripts.

The function of these transcripts is not convincingly explored as well.

Using MSL2-targeted RT&Tag we identified the roX2 ncRNA; the function of this RNA is already established, which is why we used it as true positive to evaluate RT&Tag and compare it to PIRCh-seq. The additional RNAs we detected are nascent transcripts from the X chromosome. The functions of these transcripts are irrelevant – what is relevant is that we are measurably detecting the chromosomal process of dosage compensation that the MSL2 complex directs. Similarly, with H3K27me3-targeted RT&Tag, we detected nascent transcripts from the Hox genes, revealing that chromatin repression is incomplete. Thus, RT&Tag uncovers important aspects of both upregulated gene expression and gene silencing.

16. The authors argue that simultaneously profiling both m6A and RNAPolII is outside the scope of this current manuscript. However, lack of joint RNAPolII molecule information in the current form of this method always requires separate RNAPolII experiments, making it impossible to be used in some scarce samples or even single cells.

We agree that with the current method, m6A and RNAPolII signal cannot be usefully measured in a single cell using current technologies. However, the nuclei preparation steps of RT&Tag and CUT&Tag are nearly identical, meaning that the same batch of cells can be used simultaneously for the two assays. Given the similarity in the workflow of RT&Tag and CUT&Tag, the two assays could then be easily performed side-by-side without having to do two separate experiments. In fact, this is how the heat shock experiments in Figure 5D and E were performed. For these reasons we do not see the inability to perform RT&Tag and CUT&Tag together in the same cells to be a limitation of RT&Tag.

Open Access This file is licensed under a Creative Commons Attribution 4.0 International License, which permits use, sharing, adaptation, distribution and reproduction in any medium or format, as long as you give appropriate credit to the original author(s) and the source, provide a link to the Creative Commons license, and indicate if changes were made. In the cases where the authors are anonymous, such as is the case for the reports of anonymous peer reviewers, author attribution should be to 'Anonymous Referee' followed by a clear attribution to the source work. The images or other third party material in this file are included in the article's Creative Commons license, unless indicated otherwise in a credit line to the material. If material is not included in the article's Creative Commons license and your intended use is not permitted by statutory regulation or exceeds the permitted use, you will need to obtain permission directly from the copyright holder. To view a copy of this license, visit http://creativecommons.org/licenses/by/4.0/.

Therefore, in addition to the insufficiency in demonstration of the robustness of this method, the novelty of this method and biological insights presented here are not a sufficiently high standard for me to recommend publication by Nature Methods.

Our responses above clarify how we have demonstrated the performance of the method, and highlight the biological insights we have made.

Reviewer #2:

Remarks to the Author:

The authors have addressed most of my original concerns. The introduction is improved with more details about the method and new citations are added. There are also improvements to the figures, with more in-depth analysis for RNA classification enrichment and methodology performance. The new methodology will be useful to the epigenomics community and I can now recommend publication once a few remaining minor concerns are addressed.

1. Supplementary Fig. 6B and C: to assess the reproducibility in performance of the protocol, they applied various cell numbers, each in two technical replicates, and measured the genome-wide RNA enrichment for H3K27me3-tagged regions. The paper's reproducibility measurement is an excellent improvement, but the error bars reveal large variability, and the n is just 2. With a sample size of just 2, how were error bars determined?

We thank the reviewer in pointing this out and we apologize for the inappropriate use of error bars. We should have shown just the individual data points without including the standard deviation.

2. Another strange result from this experiment is that although the reproducibility of normalized signal among three libraries is good (Supplementary Fig. 6B-top), the reads for each RNA (CPM) between the libraries are puzzling. The CPM drops when the cell number is higher. The authors should explain this.

We believe that the reviewer is referring to Supplementary Figure 6C showing IgG and H3K27me3 RT&Tag counts for the Hox genes. In this experiment, we noted a disproportionate decrease in the library size of IgG RT&Tag sample when using fewer cells. We interpret this as a decrease in background signal. Lower background result in more counts for the Hox genes as a proportion of total

Open Access This file is licensed under a Creative Commons Attribution 4.0 International License, which permits use, sharing, adaptation, distribution and reproduction in any medium or format, as long as you give appropriate credit to the original author(s) and the source, provide a link to the Creative Commons license, and indicate if changes were made. In the cases where the authors are anonymous, such as is the case for the reports of anonymous peer reviewers, author attribution should be to 'Anonymous Referee' followed by a clear attribution to the source work. The images or other third party material in this file are included in the article's Creative Commons license, unless indicated otherwise in a credit line to the material. If material is not included in the article's Creative Commons license and your intended use is not permitted by statutory regulation or exceeds the permitted use, you will need to obtain permission directly from the copyright holder. To view a copy of this license, visit http://creativecommons.org/licenses/by/4.0/.

H3K27me3 RT&Tag libraries. Given that Hox genes are transcribed at a low level, their CPMs are highly sensitive to changes in background levels.

3. Surprisingly, in Supplementary Fig. 6B-bottom, the genome browser screen shows the reverse pattern of what they show in the bar plot. Please explain?

We are reluctant to try to interpret this difference, because the data presented in Supplementary Figure 6B and Supplementary Figure 6C represent different measurements that are normalized using different tools. The bar plots in Supplementary Figure 6C display read counts normalized using DESeq2 and are a count of total reads aligning over the gene bodies of each Hox gene. The genome browser tracks in Supplementary Figure 6B are plotted using CPM normalized bigwig files generated using the bamCoverage function in deeptools. This normalization method focuses on the number of reads per bin (or a window of the genome of a defined size). Hence, we think this apparent discrepancy may be due to differences in library size normalization between the two approaches. The conclusion from both strategies is that RT&Tag signals are easily distinguished from background.

Reviewer #3:

Remarks to the Author:

My main concerns with the initial manuscript were about the nature of the associations detected. In the revised manuscript, the authors did a nice job clearly explaining what they are measuring and have addressed my comments. I remain enthusiastic about this method and would be happy to see this published in Nature Methods.

We thank the reviewer for their comments and enthusiasm.

Final Decision Letter:

Subject: Decision on Nature Methods submission NMETH-A48191B
Message:

16th Aug 2022

Open Access This file is licensed under a Creative Commons Attribution 4.0 International License, which permits use, sharing, adaptation, distribution and reproduction in any medium or format, as long as you give appropriate credit to the original author(s) and the source, provide a link to the Creative Commons license, and indicate if changes were made. In the cases where the authors are anonymous, such as is the case for the reports of anonymous peer reviewers, author attribution should be to 'Anonymous Referee' followed by a clear attribution to the source work. The images or other third party material in this file are included in the article’s Creative Commons license, unless indicated otherwise in a credit line to the material. If material is not included in the article’s Creative Commons license and your intended use is not permitted by statutory regulation or exceeds the permitted use, you will need to obtain permission directly from the copyright holder. To view a copy of this license, visit http://creativecommons.org/licenses/by/4.0/.

nature portfolio

Dear Dr Henikoff,

I am pleased to inform you that your Article, "Profiling RNA at chromatin targets in situ by antibody-targeted tagmentation", has now been accepted for publication in Nature Methods. Your paper is tentatively scheduled for publication in our Oct print issue, and will be published online prior to that. The received and accepted dates will be 24th Jan 2022 and 16th Aug 2022. This note is intended to let you know what to expect from us over the next month or so, and to let you know where to address any further questions.

Acceptance is conditional on the data in the manuscript not being published elsewhere, or announced in the print or electronic media, until the embargo/publication date. These restrictions are not intended to deter you from presenting your data at academic meetings and conferences, but any enquiries from the media about papers not yet scheduled for publication should be referred to us.

In approximately 10 business days you will receive an email with a link to choose the appropriate publishing options for your paper and our Author Services team will be in touch regarding any additional information that may be required.

Please note that *Nature Methods* is a Transformative Journal (TJ). Authors may publish their research with us through the traditional subscription access route or make their paper immediately open access through payment of an article-processing charge (APC). Authors will not be required to make a final decision about access to their article until it has been accepted. Find out more about Transformative Journals

Authors may need to take specific actions to achieve compliance with funder and institutional open access mandates. If your research is supported by a funder that requires immediate open access (e.g. according to Plan S principles) then you should select the gold OA route, and we will direct you to the compliant route where possible. For authors selecting the subscription publication route, the journal's standard licensing terms will need to be accepted, including self-archiving policies. Those licensing terms will supersede any other terms that the author or any third party may assert apply to any version of the manuscript.

You will not receive your proofs until the publishing agreement has been received through our system.

If you have any questions about our publishing options, costs, Open Access requirements, or our legal forms, please contact ASJournals@springernature.com

Open Access This file is licensed under a Creative Commons Attribution 4.0 International License, which permits use, sharing, adaptation, distribution and reproduction in any medium or format, as long as you give appropriate credit to the original author(s) and the source, provide a link to the Creative Commons license, and indicate if changes were made. In the cases where the authors are anonymous, such as is the case for the reports of anonymous peer reviewers, author attribution should be to 'Anonymous Referee' followed by a clear attribution to the source work. The images or other third party material in this file are included in the article's Creative Commons license, unless indicated otherwise in a credit line to the material. If material is not included in the article's Creative Commons license and your intended use is not permitted by statutory regulation or exceeds the permitted use, you will need to obtain permission directly from the copyright holder. To view a copy of this license, visit http://creativecommons.org/licenses/by/4.0/.

Your paper will now be copyedited to ensure that it conforms to Nature Methods style. Once proofs are generated, they will be sent to you electronically and you will be asked to send a corrected version within 24 hours. It is extremely important that you let us know now whether you will be difficult to contact over the next month. If this is the case, we ask that you send us the contact information (email) of someone who will be able to check the proofs and deal with any last-minute problems.

If, when you receive your proof, you cannot meet the deadline, please inform us at rjsproduction@springernature.com immediately.

Once your manuscript is typeset and you have completed the appropriate grant of rights, you will receive a link to your electronic proof via email with a request to make any corrections within 48 hours. If, when you receive your proof, you cannot meet this deadline, please inform us at rjsproduction@springernature.com immediately.

Once your paper has been scheduled for online publication, the Nature press office will be in touch to confirm the details.

If you have posted a preprint on any preprint server, please ensure that the preprint details are updated with a publication reference, including the DOI and a URL to the published version of the article on the journal website.

Once your paper has been scheduled for online publication, the Nature press office will be in touch to confirm the details.

Content is published online weekly on Mondays and Thursdays, and the embargo is set at 16:00 London time (GMT)/11:00 am US Eastern time (EST) on the day of publication. If you need to know the exact publication date or when the news embargo will be lifted, please contact our press office after you have submitted your proof corrections. Now is the time to inform your Public Relations or Press Office about your paper, as they might be interested in promoting its publication. This will allow them time to prepare an accurate and satisfactory press release. Include your manuscript tracking number NMETH-A48191B and the name of the journal, which they will need when they contact our office.

About one week before your paper is published online, we shall be distributing a press release to news organizations worldwide, which may include details of your work. We are happy for your institution or funding agency to prepare its own press release, but it must mention the embargo date and Nature

Open Access This file is licensed under a Creative Commons Attribution 4.0 International License, which permits use, sharing, adaptation, distribution and reproduction in any medium or format, as long as you give appropriate credit to the original author(s) and the source, provide a link to the Creative Commons license, and indicate if changes were made. In the cases where the authors are anonymous, such as is the case for the reports of anonymous peer reviewers, author attribution should be to 'Anonymous Referee' followed by a clear attribution to the source work. The images or other third party material in this file are included in the article's Creative Commons license, unless indicated otherwise in a credit line to the material. If material is not included in the article's Creative Commons license and your intended use is not permitted by statutory regulation or exceeds the permitted use, you will need to obtain permission directly from the copyright holder. To view a copy of this license, visit http://creativecommons.org/licenses/by/4.0/.

Methods. Our Press Office will contact you closer to the time of publication, but if you or your Press Office have any inquiries in the meantime, please contact press@nature.com.

To assist our authors in disseminating their research to the broader community, our SharedIt initiative provides you with a unique shareable link that will allow anyone (with or without a subscription) to read the published article. Recipients of the link with a subscription will also be able to download and print the PDF.
As soon as your article is published, you will receive an automated email with your shareable link.

You can now use a single sign-on for all your accounts, view the status of all your manuscript submissions and reviews, access usage statistics for your published articles and download a record of your refereeing activity for the Nature journals.

Nature Portfolio journals encourage authors to share their step-by-step experimental protocols on a protocol sharing platform of their choice. Nature Portfolio 's Protocol Exchange is a free-to-use and open resource for protocols; protocols deposited in Protocol Exchange are citable and can be linked from the published article. More details can found at www.nature.com/protocolexchange/about.

Please note that you and any of your coauthors will be able to order reprints and single copies of the issue containing your article through Nature Portfolio 's reprint website, which is located at http://www.nature.com/reprints/author-reprints.html. If there are any questions about reprints please send an email to author-reprints@nature.com and someone will assist you.

Please feel free to contact me if you have questions about any of these points.

Best regards,
Lei

Lei Tang, Ph.D.
Senior Editor
Nature Methods

Open Access This file is licensed under a Creative Commons Attribution 4.0 International License, which permits use, sharing, adaptation, distribution and reproduction in any medium or format, as long as you give appropriate credit to the original author(s) and the source, provide a link to the Creative Commons license, and indicate if changes were made. In the cases where the authors are anonymous, such as is the case for the reports of anonymous peer reviewers, author attribution should be to 'Anonymous Referee' followed by a clear attribution to the source work. The images or other third party material in this file are included in the article's Creative Commons license, unless indicated otherwise in a credit line to the material. If material is not included in the article's Creative Commons license and your intended use is not permitted by statutory regulation or exceeds the permitted use, you will need to obtain permission directly from the copyright holder. To view a copy of this license, visit http://creativecommons.org/licenses/by/4.0/.
